# Synergistic regulatory mechanisms in glycolysis revealed by pathway transplantation

**DOI:** 10.1128/mbio.00219-25

**Published:** 2025-12-05

**Authors:** Ewout Knibbe, Francine J. Boonekamp, Rachel Stuij, Philipp Savakis, Koen A. J. Pelsma, Liset Jansen, Carmen-Lisset Flores, Bas Teusink, Pascale Daran-Lapujade

**Affiliations:** 1 Department of Biotechnology, Delft University of Technology 201231, Delft, the Netherlands; 2 Systems Biology Lab, Amsterdam Institute of Molecular and Life Sciences, Vrije Universiteit Amsterdam, Amsterdam, the Netherlands; 3 Department of Experimental Models of Human disease, Instituto de Investigaciones Biomédicas “Alberto Sols”, Madrid, Spain; Chalmers University of Technology, Göteborg, Sweden

**Keywords:** pathway swapping, yeast, glycolysis, metabolic regulation, bistability, sugar transition

## Abstract

Protein allostery, present in all three domains of life, is key to the regulation of metabolism by allowing fast and precise control of catalysis in response to cellular demands. While metabolic pathways are frequently equipped with multiple allosterically regulated catalytic steps, experimental studies often focus on a single step, failing to capture how regulations exerted at multiple steps interact with each other for tuning pathways. Using the nearly ubiquitous Embden-Meyerhof-Parnas pathway of glycolysis as a paradigm, the present study unveils a remarkable regulatory synergy between multiple allosteric proteins of a metabolic pathway and demonstrates its impact on cell survival in dynamic environments. By using complete pathway complementation, as well as single-gene complementation, the essential regulatory steps were identified to be glucokinase, phosphofructokinase, and pyruvate kinase. Expression of these enzymes together, even in the context of the *Saccharomyces cerevisiae* pathway, led to imbalances in glycolysis that could only be overcome by lowering the glucokinase activity. Integrating these results with kinetic modeling and microfluidics experiments, the present work reveals the key synergistic role played by allosteric regulations in preventing glycolytic imbalance in the model eukaryote *Saccharomyces cerevisiae* and highlights the power of synthetic biology in addressing long-standing questions in systems biology.

**IMPORTANCE:**

All forms of life are equipped with intricate molecular mechanisms that tune their cellular responses to external and internal cues. These mechanisms are key to cells’ survival in natural environments and important for the performance of bioprocesses, which are characterized by variable environments (e.g., nutrient availability). One of these molecular mechanisms, protein allostery, enables rapid fine-tuning of the rate of cellular processes by modulating protein activity in response to metabolites *in vivo*. Using the industrial yeast and model eukaryote *Saccharomyces cerevisiae* as a paradigm, the present work reveals that, in the major route for sugar utilization known as glycolysis, three distinct allosteric regulations are critical to yeast cell survival when transitioning between carbon sources. These three regulations, while not required for pathway operation *per se*, allow efficient and balanced pathway operation under dynamic conditions. These findings, therefore, reveal a new aspect of yeast glycolysis, one of the best-studied metabolic pathways.

## INTRODUCTION

Protein allostery, present in all three domains of life, is key to the regulation of metabolism by allowing fast and precise control of catalysis in response to cellular demands. In the past 3 decades, the molecular details that govern this precise control of enzyme function have been explored for a diverse range of proteins. In parallel, mutation studies have explored the impact of protein allostery on pathway regulation. These studies typically investigate single catalytic steps, while metabolic pathways often encompass multiple steps subject to allosteric regulation. Understandably, focusing on a single allosterically regulated step prevents fully capturing interactions occurring between multiple allosterically regulated reactions and their impact on the flux in the metabolic pathway.

**Fig 1:**
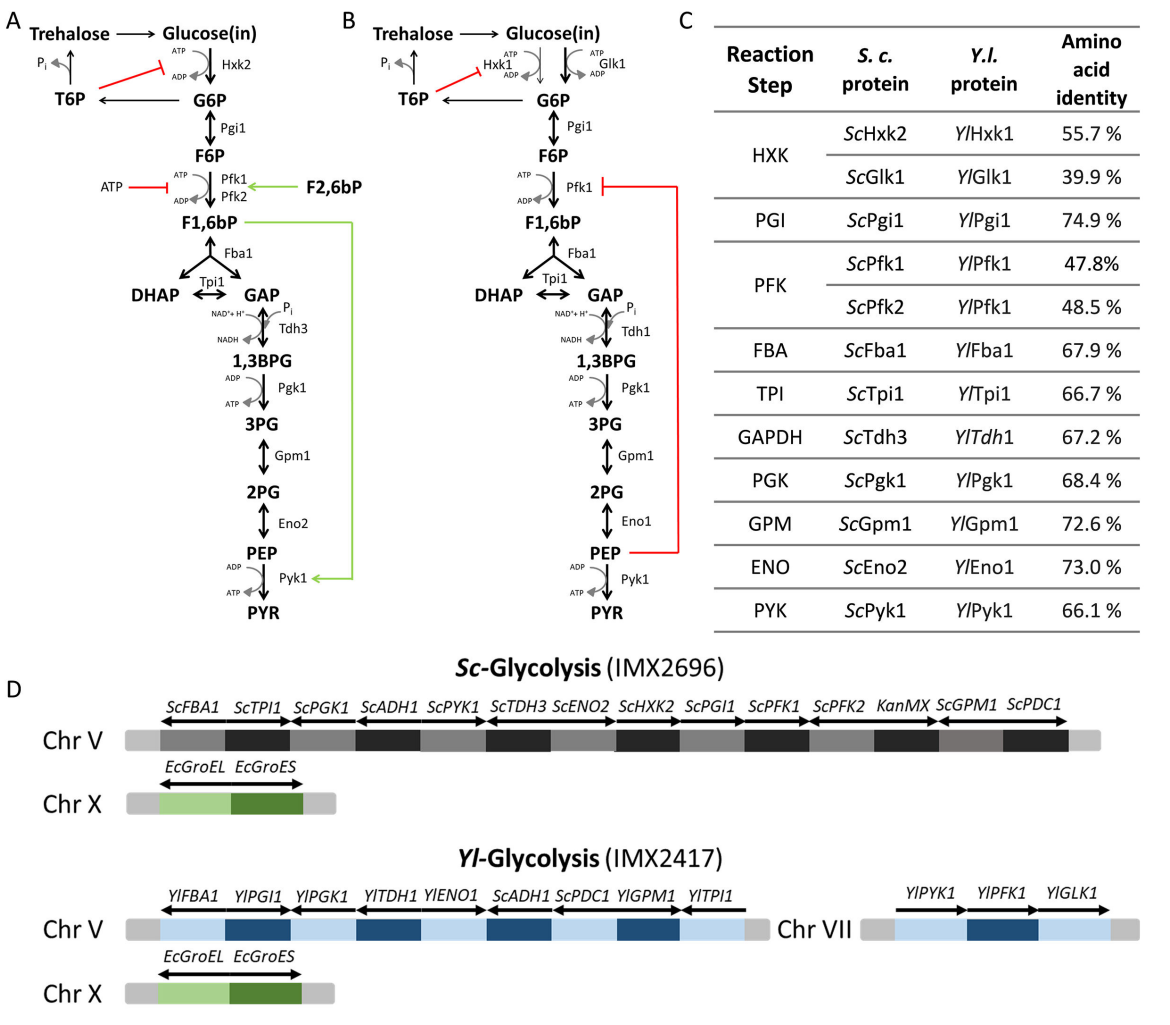
Schematic overview of the allosteric regulation of glycolysis in *S. cerevisiae* and *Yarrowia lipolytica*. (**A**) Glycolytic pathway in *S. cerevisiae*; only the major isoenzymes are shown. (**B**) Glycolysis in *Y. lipolytica* with all isoenzymes shown. In panels** A and B**, red lines indicate inhibition, and green arrows indicate activation. G6P, glucose-6-phosphate; F6P, fructose-6-phosphate; DHAP, dihydroxyacetone phosphate; GAP, glyceraldehyde-3-phosphate; 1,3BPG, 1,3-bisphosphoglycerate; 3PG, 3-phosphoglycerate; 2PG, 2-phosphoglycerate; PEP, phosphoenolpyruvate; PYR, pyruvate. (**C**) Percentage identity between *S. cerevisiae* major glycolytic isoenzymes and their *Y. lipolytica* counterparts. PGI, phosphoglucose isomerase; FBA, fructose-bisphosphate aldolase; TPI, triosephosphate isomerase; GAPDH, glyceraldehyde-3P dehydrogenase; PGK, phosphoglycerate kinase; GPM, phosphoglycerate mutase; ENO, enolase; PDC, pyruvate decarboxylase; ADH, alcohol dehydrogenase. (**D**) Schematic overview of the genetic loci containing glycolytic and chaperone genes in the control *Sc-*Glycolysis strain and the fully swapped *Yl-*Glycolysis strain.

Glycolysis is an excellent paradigm for multistep allosteric regulation. The main route for sugar utilization across all kingdoms of life, glycolysis is an important biochemical pathway in industrial biotechnology (1) and is involved in several mammalian diseases, such as cancer (via the Warburg effect) and diabetes. Eukaryotes and several prokaryotes favor the Embden-Meyerhof-Parnas (EMP) pathway of glycolysis (Fig. 1A), a set of 10 biochemical reactions largely elucidated in the model and industrial yeast *Saccharomyces cerevisiae*. Catalyzing the conversion of hexoses to pyruvate, glycolysis plays a key role in energy conservation and in the supply of precursors for biomass formation. To meet cellular demands and adjust to varying carbon and energy supplies, cells constantly need to tune the glycolytic flux in response to their environmental dynamics. While the glycolytic biochemical conversions are highly conserved between different organisms, evolution in dynamic environments has equipped cells with a myriad of multi-layered regulatory mechanisms to control the flux, ranging from gene expression regulation to modulation of the *in vivo* activity of the glycolytic enzymes through post-translational protein modifications and binding of metabolites (also known as metabolic regulation) (2–4). Metabolic regulation of glycolytic enzymes is exerted via simple mass action (concentrations of substrates and products) or through allosteric activation and inhibition by specific effectors belonging to or closely related to glycolysis. The nature of these metabolic regulations is organism-dependent and even tissue-dependent in metazoans. While functions have been linked to some regulations, e.g., phosphofructokinase inhibition by ATP to sense cellular energy demand (5) and feedforward activation of pyruvate kinase to limit accumulation of lower glycolytic intermediates (6), these are difficult to confirm experimentally, and for many regulatory interactions, a functional understanding is lacking. While the EMP pathway of *S. cerevisiae* is undoubtedly one of the most studied and best-understood examples of glycolysis, the precise physiological role of several well-described metabolic regulations remains uncertain. Hexokinase (Hxk), the first step in glycolysis, is inhibited by trehalose-6-phosphate (T6P); phosphofructokinase (Pfk) is activated by fructose-2,6-bisphosphate (F2,6bP) and is highly sensitive to energy charge; and pyruvate kinase (Pyk) is sensitive to feedforward activation by fructose-1,6-bisphosphate (F1,6bP) (Fig. 1A). While some other metabolic effectors have been described, these main metabolic regulations are thought to help balance the production and consumption of ATP in a pathway in which a first energy investment is required (ATP hydrolyzed by Hxk and Pfk) before the pay-off phase in the lower part of glycolysis (6–8). However, to date, individual complementation of these three glycolytic steps (HXK, PFK, and PYK) with insensitive variants has failed to demonstrate the purported role of these metabolic regulations in maintaining a balanced glycolysis (9–13).

While individual gene complementation is a powerful approach for the functional characterization of proteins, it might fail to capture synergistic mechanisms involving multiple proteins in a specific pathway or function. This shortcoming can be tackled by multigene complementation approaches targeting multiple enzymes at once. However, multigene complementation of essential pathways such as glycolysis is technically very challenging and is hindered in eukaryotes by a high degree of genetic redundancy in central metabolism. To address these issues and enable facile modular complementation of the entire glycolytic pathway, we engineered *S. cerevisiae* for glycolysis swapping (using the Minimal Glycolysis [MG] and Switchable Yeast Glycolysis strains [14, 15]) and recently demonstrated the two-step full humanization of *S. cerevisiae* glycolysis using these platforms (16). Glycolysis swapping can be used to challenge *S. cerevisiae*’s native metabolic regulation, for instance, by transplanting glycolytic variants from organisms that have evolved in radically different environments. The oleaginous yeast *Yarrowia lipolytica* is a pre-whole-genome duplication, Crabtree-negative yeast and is phylogenetically very distant from the *Saccharomyces* genus (17). *Y. lipolytica* thrives in environments where glucose, *S. cerevisiae*’s favorite carbon source, is scarce, instead favoring glycerol and lipids as carbon sources. Although *Y. lipolytica* can grow on hexoses, it does not ferment these to ethanol, favoring their respiratory dissimilation. These nutritional preferences have understandably shaped the regulation of *Y. lipolytica*’s glycolysis differently from *S. cerevisiae*. All steps in *Y. lipolytica* glycolysis are catalyzed by a single isoenzyme, with the exception of the glucose phosphorylation step (Fig. 1B and C). *Y. lipolytica* relies mostly on a glucokinase (Glk), insensitive to any known allosteric regulators, while Hxk, sensitive to T6P inhibition and known to complement in *S. cerevisiae*, plays a minor role (18, 19). Furthermore, while Pfk is a hub of allosteric regulation in many organisms, including *S. cerevisiae*, the *Y. lipolytica* Pfk is only strongly inhibited by phosphoenolpyruvate. Despite these differences in metabolic regulation, *Y. lipolytica* Pfk has been shown to complement its *S. cerevisiae* counterpart when expressed in *S. cerevisiae* (12). Finally, *Y. lipolytica* pyruvate kinase is not affected by F1,6bP, the activator of *S. cerevisiae* Pyk1 (20). The *Y. lipolytica* glycolytic pathway therefore lacks most of the allosteric regulations found in the model yeast *S. cerevisiae*.

Using *S. cerevisiae* glycolysis as a paradigm, the present study leverages the potential of partial and full pathway swapping to identify the metabolic role of complex allosteric regulations.

## RESULTS

### General experimental approach for pathway transplantation

While *S. cerevisiae* has a set of 26 glycolytic enzymes, the presence of a single enzyme for 9 out of the 10 glycolytic steps in *Y. lipolytica* simplified the choice of genes to transplant. The complete set of 10 *Y. lipolytica* enzymes leading to the conversion of glucose to pyruvate was transplanted in *S. cerevisiae* using the SwYG strain, harboring a minimal set of glycolytic genes relocated to a single locus (14). The glucokinase (*Yl*Glk1) was chosen for expression in *S. cerevisiae* as part of the complete pathway, since this is considered the major isoenzyme in *Y. lipolytica* and because of its insensitivity to T6P regulation. The *Escherichia coli* folding chaperones GroEL and GroES were co-expressed to facilitate functional expression of *Y. lipolytica* enzymes (21). The constructed IMX2417 strain, called *Yl*-Glycolysis, was devoid of *S. cerevisiae* glycolytic genes and entirely relied on the *Y. lipolytica* glycolysis for glucose dissimilation (Fig. 1D). A reference strain, IMX2696 (named *Sc*-Glycolysis), expressing the native, minimized *S. cerevisiae* glycolytic pathway from the same chromosomal locus and expressing the GroEL/GroES chaperones, was also constructed. It is important to note that, to minimize the requirement for fast glycolytic flux and the risk of glycolytic imbalance, during all construction steps, strains expressing *Yarrowia* glycolytic genes were purposely grown on galactose or on a mixture of ethanol and glycerol, but not on glucose. Galactose conversion to the glycolytic intermediate glucose-6-phosphate (G6P) occurs via the Leloir pathway in yeast. The lower capacity of this pathway compared to hexokinase (converting glucose to G6P) enables growth of strains with regulatory deficiencies, such as *tps1* deletion strains (22, 23).

### *S. cerevisiae* with a *Y. lipolytica* glycolysis displays growth defects on glucose medium

**Fig 2:**
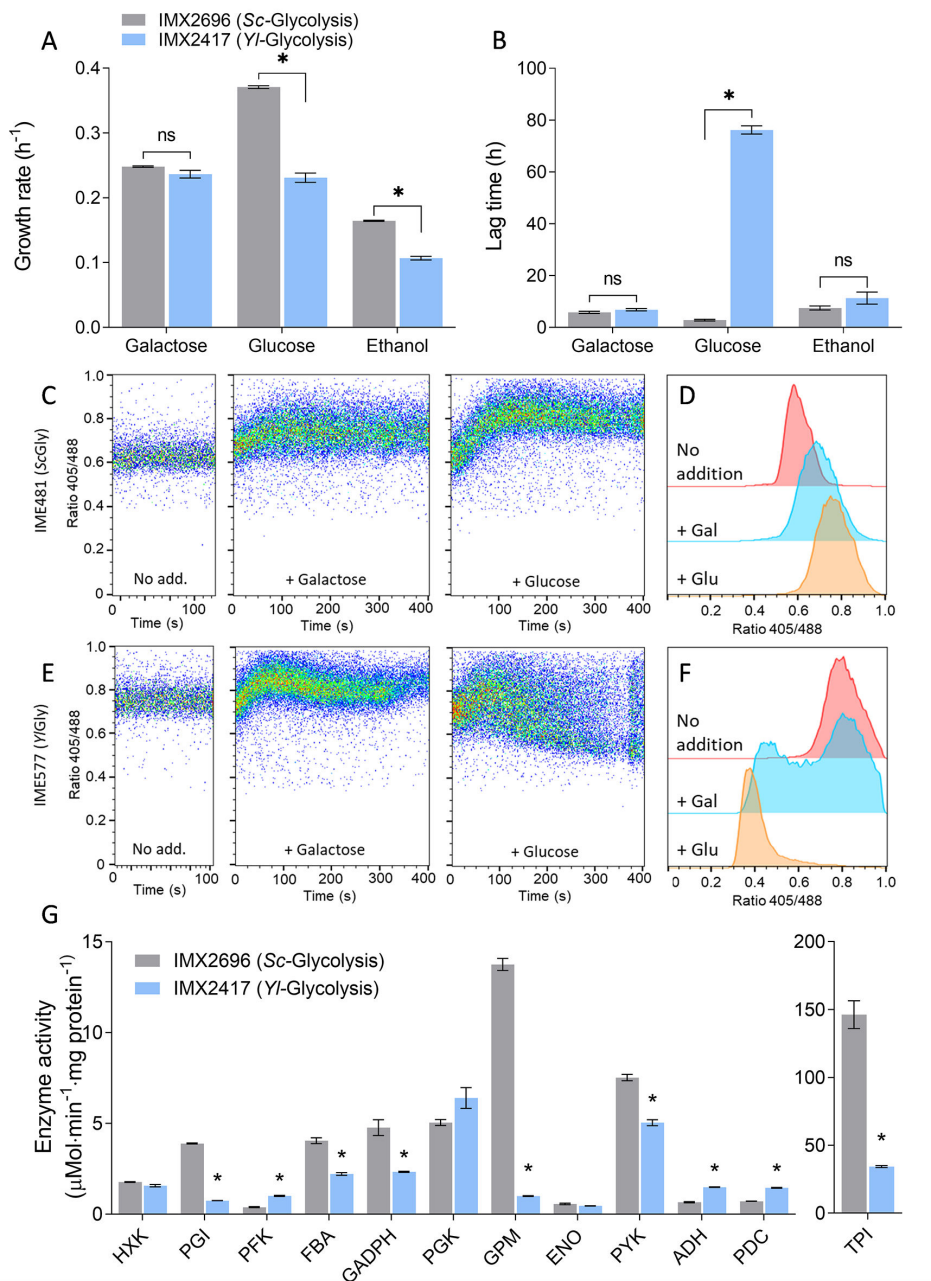
Characterization of a fully swapped glycolysis strain. (**A**) Growth rates measured in the growth profiler on galactose, glucose, and ethanol for the full *Yarrowia lipolytica* glycolysis strain (*Yl-*Glycolysis, IMX2417) and a control strain expressing the native *S. cerevisiae* glycolytic pathway from a single locus (*Sc-*Glycolysis, IMX2696). (**B**) Lag-phase duration upon transfer from SM-galactose to SM with either galactose, glucose, or ethanol. For panels A and B, the mean and SEM of triplicates are shown. * indicates significant difference (*t*-test, homoscedastic, unpaired, with *P*-value <0.05). (**C** and E) Dynamics of intracellular pH, measured as pHluorin signal ratio, in a strain with the native glycolytic pathway (**C**) and the *Yl-*Glycolysis strain (**E**), without and immediately after galactose or glucose addition. (**D** and **F**) pHluorin signal ratio in the population of the native glycolysis strain IME481 (**D**) and the *Yl-*Glycolysis strain (**F**) after incubation without carbon-source addition (red; after 56 and 62 min for panels **D** and **F**, respectively) or with galactose (blue; after 66 and 72 min) or glucose (orange; after 76 and 82 min). Duplicate pHluorin experiments are shown in Fig. S1 and S2. (**G**) *In vitro* enzyme activities of the glycolytic enzymes in the *Yl-*Glycolysis and *Sc-*Glycolysis strains grown on galactose. Mean and SEM of triplicates are shown. * indicates significant differences (*t*-test, homoscedastic, unpaired, with *P*-value <0.05).

Upon full pathway transplantation, growth on galactose in minimal, chemically defined medium was immediately observed for the *Yl*-Glycolysis strain IMX2417 (Fig. 2A and B), proving functional expression in *S. cerevisiae* of all *Yarrowia lipolytica* glycolytic enzymes besides glucokinase (*Yl*Glk1). Growth on ethanol was similarly observed immediately, although at a reduced rate. However, the same strain could not readily grow upon transfer to glucose medium and displayed slow but exponential growth only after 3 to 4 days of incubation (Fig. 2B). Intracellular pH (pH_i_) is a good indicator of the cellular metabolic status, considering that cells unable to conserve energy are incapable of maintaining pH homeostasis (22, 24, 25). Using pHluorin as a proxy for pH_i_ showed that addition of either glucose or galactose to a galactose-grown *Sc*-Glycolysis strain did not cause a decrease in pH_i_, but instead a slight increase, consistent with previous data of control strains (Fig. 2C and D; Fig. S1) (22, 24). A similar response was observed immediately after galactose addition to the *Yl-*Glycolysis strain (Fig. 2E), while addition of glucose to the medium caused a substantial and rapid drop in pH_i_, which lasted for a prolonged period of time (Fig. 2E and F; Fig. S2), matching the initial lack of growth observed on glucose for this strain. This response to glucose might have several causes, such as a non-functional *Y. lipolytica* glucokinase, an insufficient capacity of the *Y. lipolytica* glycolytic enzymes during growth on glucose, or a regulatory deficiency preventing glucose catabolism by otherwise functional glycolytic enzymes. To determine the functionality of *Y. lipolytica* glycolytic enzymes expressed in *S. cerevisiae*, *in vitro* assays were performed on the *Yl*-Glycolysis strain grown on galactose. All *Y. lipolytica* glycolytic enzymes, including glucokinase, showed activity in *in vitro* assays from cell extracts of galactose-grown cultures (Fig. 2G). Compared to the *Sc*-Glycolysis reference strain IMX2696, the *Y. lipolytica* phosphoglucose isomerase (PGI), triosephosphate isomerase (TPI), and phosphoglycerate mutase (GPM) showed substantially lower specific activities (19%, 23%, and 7% of the activity in the *Sc-*Glycolysis strain, respectively), while the kinases GLK, PFK, and PYK, which were expressed as codon-optimized genes, were highly active *in vitro* (89%, 259%, and 67% of *Sc-*Glycolysis strain activity).

The *Y. lipolytica* glycolytic enzymes were all expressed in *S. cerevisiae* and functional *in vitro*. Fast growth on galactose (0.24 ± 0.01 h^−1^ as compared to 0.25 ± 0.00 h^−1^ for the control *Sc-*Glycolysis strain, Fig. 2A) demonstrated that enzymes from phosphoglucose isomerase down to pyruvate kinase were active *in vivo* and able to carry the glycolytic flux, although the lower activities of some enzymes might be responsible for the lower growth rates measured in ethanol and glucose media. Growth on galactose but inability to grow upon transition to glucose of the *Yl-*Glycolysis strain, despite substantial *in vitro* activity of *Yl*Glk, suggested a defect in *Yl*Glk activity *in vivo* or a regulatory imbalance in the pathway. The appearance of growth after an extended lag phase upon galactose/glucose transition could be explained by the resolution of *Yl*Glk *in vivo* activity defects either by evolution or metabolic adaptation. Evolution entails the occurrence of mutations that endow a few cells with the ability to grow and take over the whole population in adverse conditions; this feature is heritable. Metabolic adaptation reflects population heterogeneity in terms of metabolic status and the ability of a very small fraction of the population with the appropriate metabolic status to grow and outcompete the rest of the population. Contrary to evolution, metabolic adaptation is not heritable and systematically results in a lag phase each time a new galactose/glucose transition is performed.

### Full pathway swapping reveals that glucose phosphorylation is a key regulatory node for glycolysis transplantation in *S. cerevisiae*

**Fig 3:**
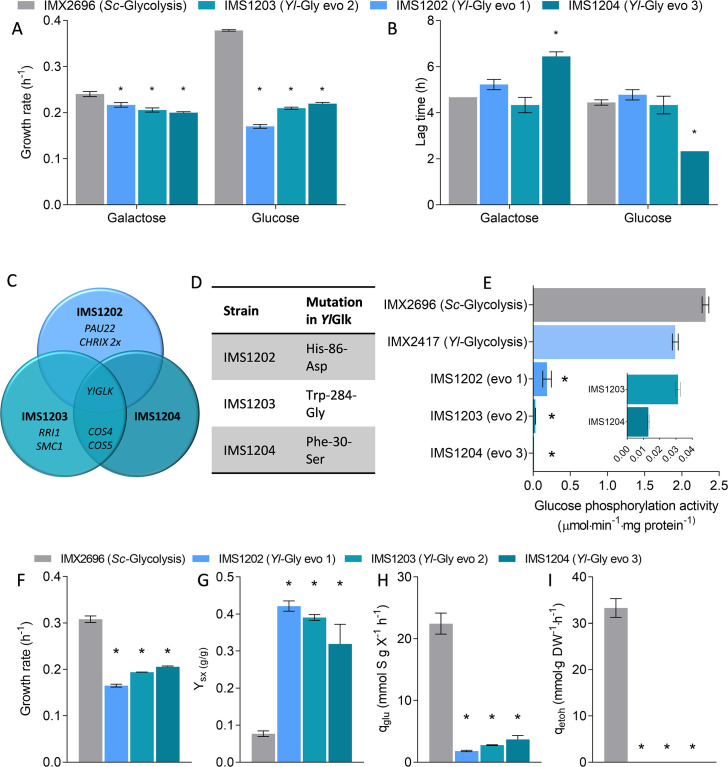
Characterization of glucose-grown isolates of the *Yl*-Glycolysis strain. (**A**) Growth rates on galactose and glucose of the *Sc-*Glycolysis reference strain and of three single-colony isolates obtained after serial transfers of the *Yl-*Glycolysis strain on glucose; data from biological triplicates. (**B**) Duration of lag phase upon galactose/galactose and galactose/glucose transition for the same strains as in panel A. (**C**) Genes with mutations in the three independent isolates of the glucose-grown *Yl-*Glycolysis strain; *CHRIX* 2x indicates duplication of chromosome 9. (**D**) Mutations in the *Y. lipolytica* glucokinase observed in each of the three isolates. (**E**) *In vitro* glucose phosphorylation activity in the *Yl-*Glycolysis strain and in the evolved isolates measured in duplicate. To enable comparison with the non-mutated *Yl-*Glycolysis strain IMX2417, all strains were grown in medium with galactose as the sole carbon source. (**F–I**) Physiological characterization of the evolved *Yl*-Glycolysis strains and control *Sc-*Glycolysis strain during growth on SM-glucose urea medium in duplicate shake flasks. Growth rate, estimated biomass yield (Y_SX_), specific glucose uptake rates (q_glu_), and specific ethanol production rates (q_etoh_) are shown. For all panels, * indicates significant differences from control (*t*-test, homoscedastic, unpaired, with *P*-value <0.05).

To identify whether the delayed growth on glucose of the *S. cerevisiae* strain expressing *Yarrowia* glycolysis resulted from evolution or adaptation, serial transfers of the *Yl-*Glycolysis strain in media alternating galactose and glucose as carbon sources were performed. The long lag phase disappeared in the second and following galactose/glucose transitions, revealing that the ability to grow on glucose of the *Yl-*Glycolysis strain was most probably of genetic origin (Fig. S3). Systematic mutations in *YlGLK1* were accordingly found in single colonies isolated from three independent glucose-grown cultures (Fig. 3D and Fig. S3B). All three isolates were capable of fast growth on glucose without a lag phase (strains IMS1202 to 1204, Fig. 3A and B). Whole-genome sequencing identified mutations leading to single, distinct amino acid substitutions in all isolates, scattered over the *Yl*Glk protein (Fig. S4). *In vitro* assays revealed a marked (10- to 146-fold) decrease in glucokinase activity for the three mutated *YlGLK* variants as compared to the native enzyme (Fig. 3E and Fig. S5), and an increase in the apparent *K*_m,glucose_ for two of them (Fig. S5A). More detailed physiological characterization of the evolved strains in shake flasks on glucose medium with urea as the nitrogen source showed reduced growth rates as compared to the *Sc-*Glycolysis strain (ca. twofold decrease, Fig. 3F). This decrease in growth rate corresponded to a fully respiratory metabolism, with a higher biomass yield, a strong decrease in glucose uptake rate, and the absence of ethanol production (Fig. 3G through I). The lower flux through glycolysis appeared to match the lower glucokinase activity in these strains, although for IMS1203 and IMS1204, the *in vitro* glucokinase activity was too low to sustain the *in vivo* glucose uptake rate (Fig. S6). This discrepancy suggested an underestimation of the *in vivo* glucokinase activity based on *in vitro* enzyme assays for the evolved strains. Nonetheless, the ability to grow readily on galactose but not on glucose of the *Yl-*Glycolysis strain, the systematic requirement for mutations in glucokinase for growth on glucose, the decreased *in vitro* glucokinase activity, and the low glucose uptake rate in independent isolates suggested that the activity of glucokinase, the first step in glycolysis during glucose utilization, might be too high for growth on glucose in the *Yl-*Glycolysis strain.

### The glycolytic context determines the physiological impact of glucokinase deregulation

**Fig 4:**
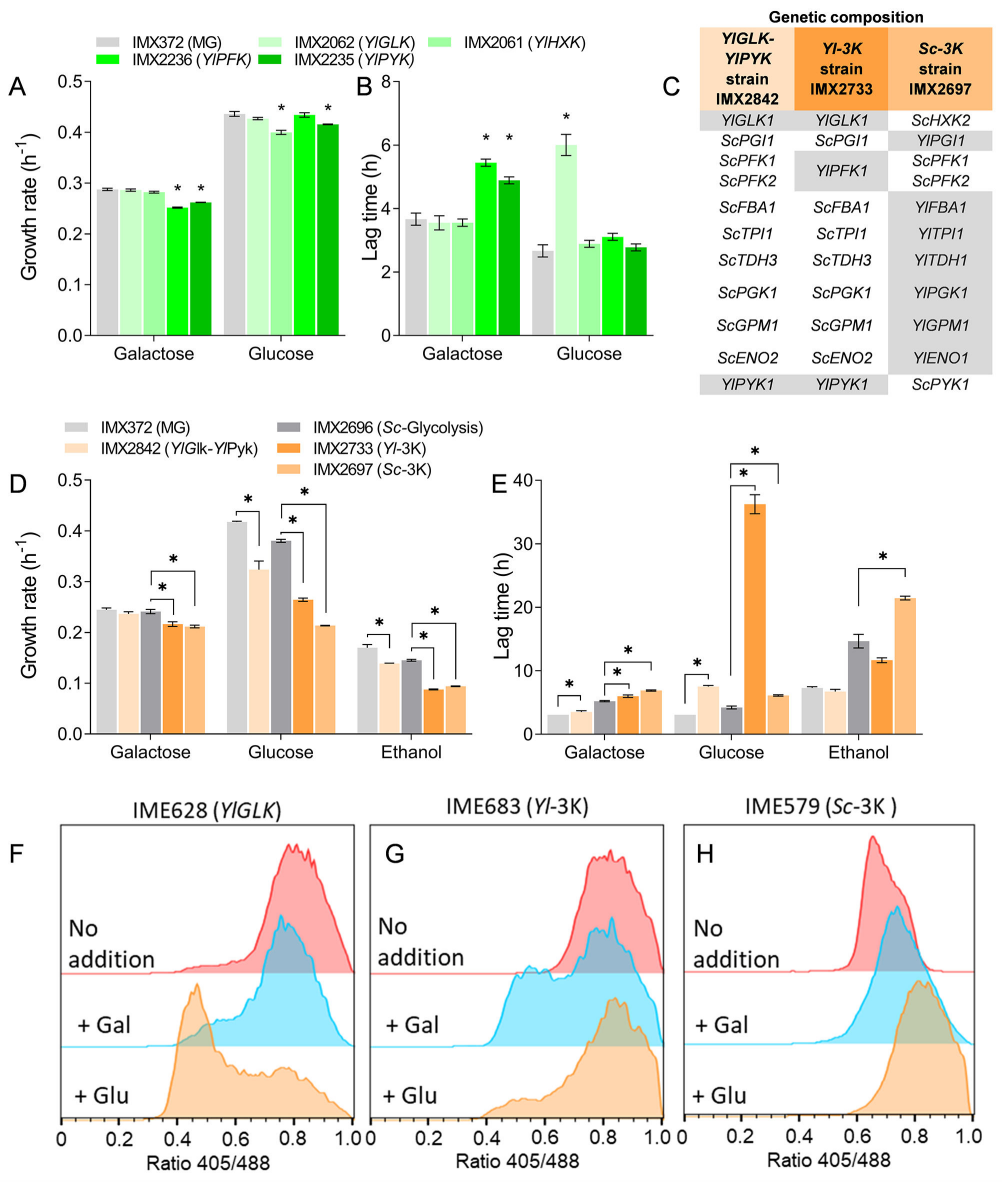
Response of complementation and “mosaic” glycolysis strains to glucose and galactose. (**A** and **B**) Growth rate and lag-phase duration of single *YlGLK*, *YlHXK, YlPFK,* and *YlPYK* complementation strains. Measured in biological triplicates; * indicates significant differences from the reference strain (*t*-test, homoscedastic, unpaired, with *P*-value <0.05). (**C**) Composition of the glycolysis of the *YlGLK-YlPYK, Yl-*3K, and *Sc-*3K strains. *Y. lipolytica* genes are indicated in gray. (**D** and **E**) Growth rate and lag-phase duration of the “mosaic” glycolysis strains *YlGLK-YlPYK, Yl*-3K, *Sc*-3K, and the control strains with native, minimized glycolysis IMX372 (MG) and IMX2696 (*Sc-*Glycolysis). Measured in biological triplicates; * indicates significant differences from the reference strain (*t*-test, homoscedastic, unpaired, with *P*-value <0.05). (**F–H**) pHluorin-based pH_i_ profiles of strains grown on galactose transferred to glucose and galactose. (**F**) *YlGLK* complementation strain, (**G**) *Yl-*3K strain, and (**H**) *Sc-*3K strain. Duplicate pHluorin experiments and time-course data are shown in Fig. S2 and S8.

Previous studies have shown that *S. cerevisiae* hexokinase can be successfully complemented by heterologous, “unregulated” variants insensitive to known effectors (9, 10, 16). The requirement for mutations in *YlGLK1* for growth on glucose of *S. cerevisiae* and their impact on the kinetic properties of glucokinase was therefore unexpected and required further investigation. The major difference with these earlier studies was the presence of the complete set of glycolytic enzymes from *Yarrowia lipolytica* in the present study. The ability of T6P-insensitive variants to complement *Sc*Hxk2 might therefore be context-dependent. To test this hypothesis, a single *YlGLK* complementation strain was constructed using the MG strain (15), a strain with a minimized set of glycolytic genes, specifically engineered to facilitate complementation studies. The *YlHXK* gene, encoding the *Y. lipolytica* hexokinase, which is strongly inhibited by T6P, was similarly tested for complementation. During galactose/glucose transitions, the *YlGLK* single complementation strain (IMX2062) largely responded as its parent strain MG with the complete set of *S. cerevisiae* enzymes, with an identical growth rate on glucose (Fig. 4A). The IMX2062 strain’s lag phase on glucose was slightly longer than that of MG (6.0 ± 0.6 hours vs 2.7 ± 0.3 hours, Fig. 4B) but was substantially shorter than the 3 to 4 days required for the *Yl-*Glycolysis strain. The *YlHXK* strain harboring the T6P-sensitive hexokinase did not show any measurable lag phase and only a very slight effect on growth rate. For both strains, characterization in shake flasks showed similar growth rates, and both strains produced ethanol when grown on glucose, only at a slightly lower rate (Fig. S7). The intracellular pH, however, showed a decrease upon glucose addition in the *YlGLK* complementation strain, matching the slightly longer adaptation time required for galactose/glucose transition (Fig. 4F), but the effect was not as severe as in the *Yl-*Glycolysis strain. Expression of *YlGLK1* therefore visibly but mildly affected the ability of *S. cerevisiae* to transition from galactose to glucose and did not result in mutation of *YlGLK1* upon repeated transfers between galactose and glucose (Fig. S3). Single complementation of the *S. cerevisiae* hexokinase with the T6P-insensitive *Yl*Glk1 was therefore not recapitulating the phenotype of the strain with a full *Y. lipolytica* glycolysis.

Next to hexokinase, phosphofructokinase and pyruvate kinase are considered as “pacemakers” in *S. cerevisiae* glycolysis and are subject to allosteric regulations that are absent in their *Y. lipolytica* orthologs. To check if these differential regulations affected *S. cerevisiae* physiology, more particularly during carbon source switches, single complementation strains were also constructed for *YlPFK* and *YlPYK*, resulting in strains IMX2236 and IMX2235, respectively. These complementation strains grew with rates close to the MG control strain on glucose (0.43 ± 0.007 and 0.42 ± 0.001 h^−1^ compared to 0.44 ± 0.008 h^−1^), and displayed neither lag phase nor pH_i_ decrease upon transition from galactose to glucose (Fig. 4A and B; Fig. S8). This absence of phenotype for individual complementation with insensitive isoenzymes was in line with earlier reports for phosphofructokinase (12, 13) and pyruvate kinase (16, 26). In line with these observations, a strain expressing the entire *Y. lipolytica* glycolysis gene set except the phosphofructokinase (strain IMX2734), encoded by the native *ScPFK1* and *ScPFK2* genes, responded very similarly to the *Yl*-Glycolysis strain with full *Y. lipolytica* glycolysis during growth on glucose, further supporting the notion that single enzymes were not responsible for the growth defect on glucose of the *Yl*-Glycolysis strain (Fig. S2 and S9).

It is important to stress that single complementation strains were constructed with the same glycolytic expression cassettes as the *Yl-*Glycolysis strain (see Table S2 for composition of the expression cassettes). Individual deregulation of hexokinase, phosphofructokinase, and pyruvate kinase was therefore not affecting the ability of *S. cerevisiae* to switch between galactose and glucose, revealing that functional expression of the *Y. lipolytica* enzymes was dependent on the glycolytic context.

### Simultaneous expression of deregulated glycolytic kinases reproduces the glycolytic imbalance phenotype

While individual expression of the *Y. lipolytica* key kinases did not affect *S. cerevisiae* phenotype, the coordinated regulation of these three kinases or a combination of two of them might still play an important role in metabolic adaptation to sugar transition. To test this hypothesis, several strains with “mosaic” glycolytic configurations were constructed. Strain IMX2842 (*Yl*Glk-*Yl*Pyk) had the native *S. cerevisiae* glycolysis except for the *Y. lipolytica* glucokinase and pyruvate kinase. Strain IMX2733 (*Yl-*3K strain) harbored the native *S. cerevisiae* glycolysis but with the three *Y*. *lipolytica* kinases (glucokinase, phosphofructokinase, and pyruvate kinase), while strain IMX2697 (*Sc-*3K strain) carried the *Y. lipolytica* glycolysis with the three *S*. *cerevisiae* kinases (Fig. 4C). The response to glucose exposure of these strains was radically different. The *Yl*Glk-*Yl*Pyk and *Sc-*3K strains showed phenotypes similar to the *S. cerevisiae* control strains with native glycolysis, albeit with a somewhat slower growth rate (78 ± 4% and 56 ± 0.2% of the control strain growth rate on glucose, Fig. 4D), possibly caused by lower activities of some of the *Y. lipolytica* enzymes in *S. cerevisiae*, as determined in the *Yl*-Glycolysis strain (Fig. 2G). Conversely, the *Yl-*3K strain showed a long lag phase reminiscent of the full *Yl-*Glycolysis strain, although the duration of this lag phase was clearly shorter (36 ± 3 hours compared to 76 ± 3 hours on average for the *Yl-*3K and *Yl-*Glycolysis strains, Fig. 4E). The pH_i_ response upon exposure to glucose was present but not as marked in the *Yl-*3K as in the *Yl-*Glycolysis strain (Fig. 4 and Fig. S2).

**Fig 5:**
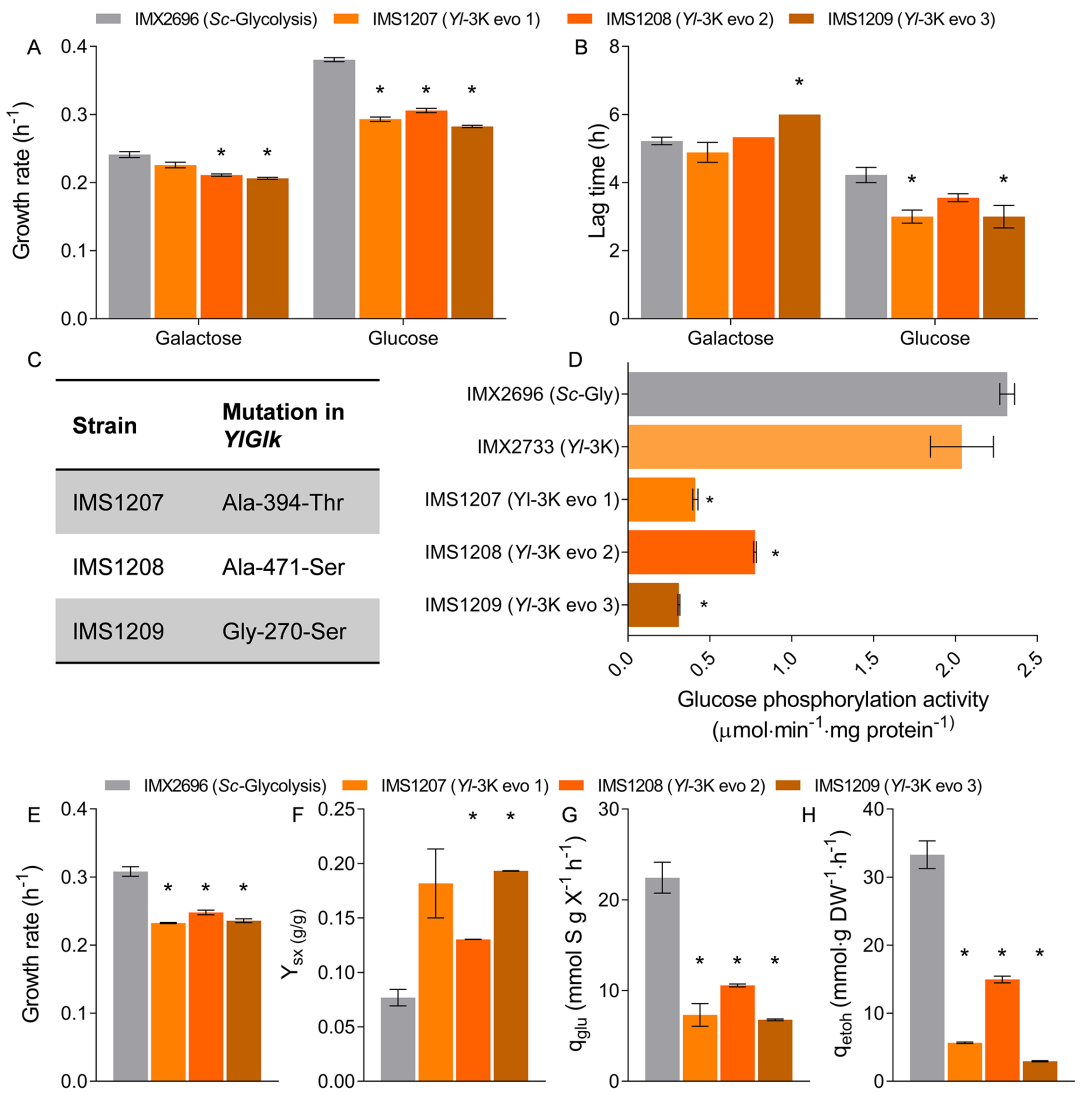
Glucose-grown isolates of a mosaic glycolysis strain and lag-phase dependency on glucose concentration. (**A** and **B**) Growth rate and lag-phase duration on glucose and galactose media of three independent isolates of the *Yl-*3K strain (IMX2733) that acquired the ability to grow on glucose; biological triplicates were measured. (**C**) Mutations in the *Yl* glucokinase found in each of the three isolates. (**D**) *In vitro* glucokinase activity of the *Yl-*3K strain and its evolved isolates determined from biological duplicate SM-galactose cultures. (**E–H**) Physiological characterization of the three evolved isolates of the *Yl-3*K strain and the *Sc-*Glycolysis reference strain on glucose urea medium in shake flasks in biological duplicates. The same data for *Sc*-Glycolysis strain IMX2696 are shown as in Fig. 3F through I as control. Growth rates, biomass yield (Y_SX_), specific glucose uptake rates (q_glu_), and specific ethanol production rates (q_etoh_) are shown. In all panels, asterisks indicate significant differences compared to the relevant reference strain (*t*-test, homoscedastic, unpaired, with *P*-value <0.05).

Despite this somewhat milder response of the *Yl-*3K strain, as compared to the strain with full *Y. lipolytica* glycolysis, to glucose medium, repeated glucose/galactose transfers also resulted in systematic mutations in *YlGLK1* (A394T, A471S, and G270S) and in decreased glucokinase activity *in vitro* (Fig. S3; Fig. 5A through D, single-cell isolates IMS1207 to IMS1209). Characterization in shake flasks of these evolved strains showed low specific growth and glucose uptake rates, as compared to the *Sc*-Glycolysis control strain, but some ethanol production was observed, unlike for the evolved *Yl*-Glycolysis strain isolates (Fig. S6). The lower specific growth and glucose uptake rates of the evolved strains, as compared to the *Sc*-Glycolysis control strain, are most likely explained by a limited capacity of the mutated glucokinases. Indeed, in these strains, the maximum glycolytic flux estimated from *in vitro* glucokinase activity was similar to the glycolytic flux estimated from the glucose uptake rate (Fig. 5E through H and Fig. S6). These results brought new insight into the genotype-to-phenotype relationship of the *Yl*-Glycolysis strain. Firstly, the absence of any phenotype during transition to glucose of the *Sc*-3K strain revealed that the relatively low activity of several of the glycolytic enzymes in the *Yl*-Glycolysis strain was not causing the deficiency in transitioning between carbon sources. However, these lower activities were probably responsible for the slower growth rate of the *Yl*-Glycolysis strain, as compared to the control strain with native *S. cerevisiae* glycolysis. Secondly, the combined expression of the three deregulated *Y. lipolytica* kinases was identified as the major cause of the growth and transition defects of the *Yl*-Glycolysis strain.

### Reducing the flux through top glycolysis compensates for the lack of metabolic regulation of the *Y. lipolytica* kinases in *S. cerevisiae*

**Fig 6:**
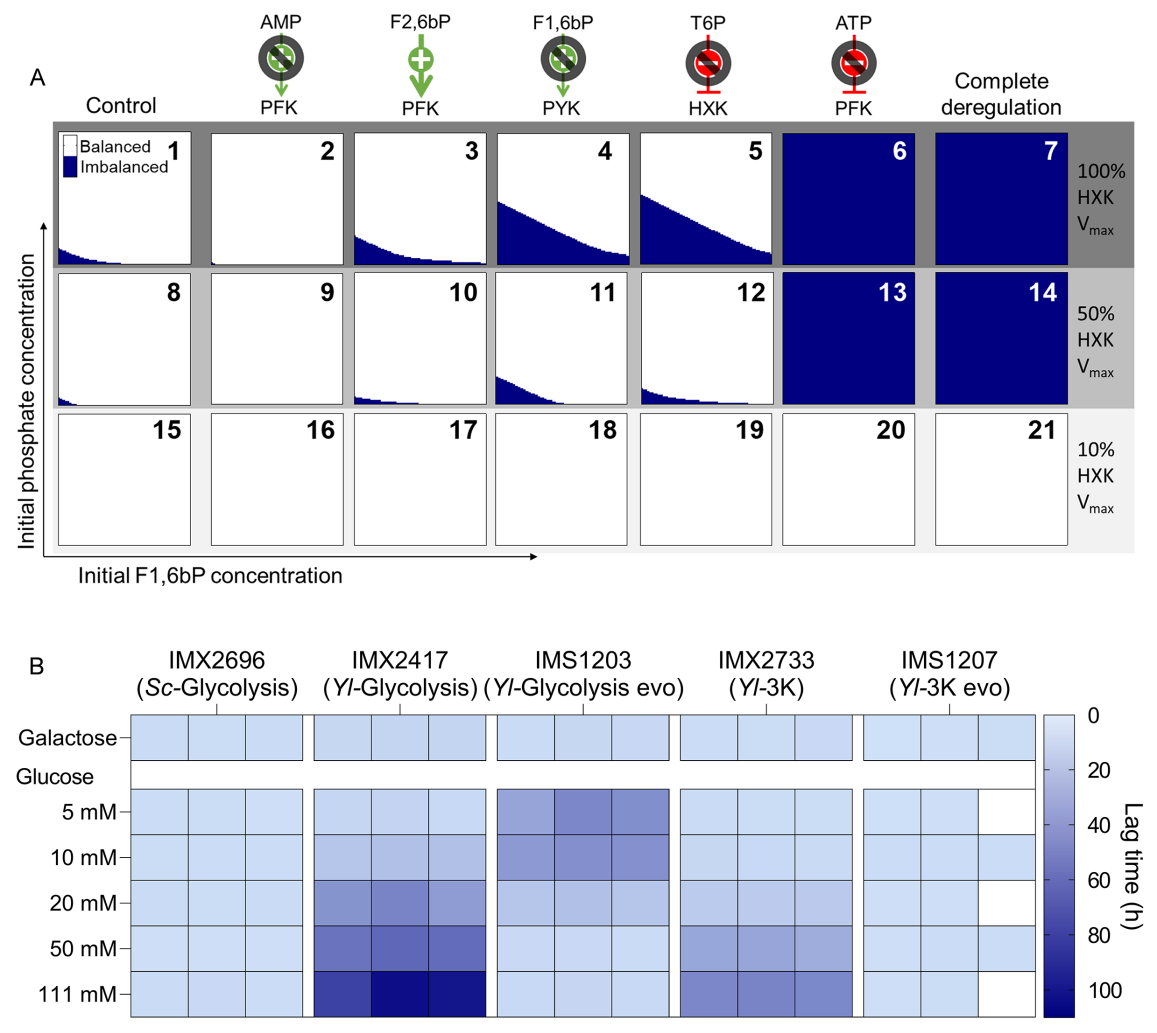
Kinetic modeling to study the impact of deregulation of glycolysis. (**A**) Results from kinetic modeling analysis of balanced and imbalanced states. For each graph, the *x*-axis indicates the initial fructose-1,6-bisphosphate concentration (between 0 and 5 mM) and the *y*-axis the initial phosphate concentration (between 0 and 20 mM), with identical conditions used for each plot. White coloring indicates the model reaches a balanced steady state with those conditions within 100 min; dark blue coloring shows imbalance. In the top left (plot 1), the situation in the unaltered control model is shown, which has a small imbalanced region at low phosphate and F1,6bP concentrations. For each of the five allosteric regulations in yeast glycolysis, the impact of their removal on the stability of the system is shown, as well as the impact of combinations of removal of all regulations simultaneously. Tested regulations were removal of AMP activation on PFK (2, 9, 16), constant F2,6bP activation of PFK (3, 10, 17), removal of F1,6bP activation on PYK (4, 11, 18), removal of T6P inhibition on HXK (5, 12, 19), removal of ATP inhibition of PFK (6, 13, 20), and combination of removal of all together (7, 14, 21). The lower rows show the impact of hexokinase activity on the stability of each system, with the second row (8–14) showing 50% hexokinase *V*_max_ and the bottom row (15–21) 10% hexokinase *V*_max_. (**B**) Dependency of the lag-phase duration of the *Yl*-Glycolysis and *Yl-*3K strains and two of their evolved isolates on the glucose concentration. 111 mM galactose is shown as the control condition; growth rates and lag-phase duration are shown in Fig. S12. Individual replicates are shown for each strain; those marked in white were not measured.

The results suggested that lowering the glycolytic flux by limiting the rate of glucose phosphorylation was the optimal cellular strategy of the *Yl-*Glycolysis and *Yl*-3K strains to balance glycolysis under glucose excess. Kinetic modeling was used to explore whether reducing hexokinase activity could indeed compensate for the absence of *S. cerevisiae*-like regulation of the three kinases and to guide further experimental design. Studying a *tps1*Δ mutant, Van Heerden et al. demonstrated that the Embden-Meyerhof-Parnas glycolysis can switch between balanced and imbalanced states depending on the genetic and metabolic context (22). Mutants with a *tps1* deletion cannot synthesize T6P, a regulator of *S. cerevisiae* hexokinases, and cannot maintain inorganic phosphate (P_i_) homeostasis, resulting in the inability to grow under high-glucose conditions. Cellular levels of F1,6bP and P_i_ are key determinants of glycolysis stability in *tps1* mutants. Considering cell-to-cell variation in F1,6bP and P_i_ concentrations upon exposure to glucose, only the fraction of cells with the appropriate concentrations can reach a balanced state and resume growth. Van Heerden and colleagues used a kinetic model of glycolysis to describe and predict the start-up of glycolysis as a function of F1,6bP and P_i_ levels. The reference *S. cerevisiae* strain with native regulation displayed a balanced glycolysis for a wide range of physiologically relevant F1,6bP and P_i_ levels (Fig. 6A, plot 1). Only at very low initial F1,6bP and P_i_ concentrations did glycolytic imbalance occur, resulting in intracellular accumulation of F1,6bP and ATP depletion (Fig. S10). Such an imbalance would consequently lead to the inability to grow on glucose of the small fraction of the cellular population that occupied this concentration range at the moment of glucose exposure. Starting from this model, the regulations of the three kinases were removed, individually as well as simultaneously, to mimic *in silico* the replacement of *S. cerevisiae* kinases by their *Y. lipolytica* variants. Removing the regulations individually (Fig. 6A plots 2 to 6) or simultaneously (plot 7) resulted in most cases in an increase in conditions leading to the imbalanced state, with the strongest effect obtained by removing ATP inhibition of PFK or combining removal of multiple regulations. Combinatorial removal of multiple regulations also resulted in a decrease of balanced model outcomes (Fig. S11), showing their overlapping effects. The strong impact of PFK regulation in the model did not match our experimental observations (Fig. 4A). PFK is an enzyme with a complex regulatory pattern, and its activity has been proven to be difficult to mathematically describe in *S. cerevisiae* (8, 27). Using an imperfect *S. cerevisiae* PFK model to reflect *Yarrowia*’s variant most likely exacerbates this problem, resulting in an *in silico* oversensitivity to phosphofructokinase regulation and activity. However, the overall modeling response agreed well with experimental data, as factors increasing the flux through HXK and PFK destabilized glycolysis, while decreasing the flux through upper glycolysis (e.g., by decreasing activation on PFK) stabilized it. Accordingly, reducing the hexokinase *V*_max_ in the model resulted in higher glycolytic stability, with all model configurations reaching a balanced state at 10% of the original hexokinase activity (Fig. 6A, plots 8–21).

To experimentally test whether high flux in upper glycolysis was the main causal factor of the growth defect on glucose of the *Yl-*Glycolysis and *Yl-*3K strains, glucose uptake rate was tuned by exposing the strains to media with varying glucose concentrations (5, 10, 20, 50, and 111 mM; Fig. 6B and Fig. S12). As expected, the lag phase of evolved strains with *YlGLK* mutations (IMS1203 and IMS1207) was mostly insensitive to glucose concentration. Conversely, for both *Yl-*Glycolysis and *Yl-*3K strains, while specific growth rate was only marginally affected by glucose concentrations, the lag-phase duration was strongly positively correlated with glucose concentration. At the lowest tested glucose concentration of 5 mM, growth resumed upon transfer to glucose medium without lag phase, demonstrating that cells could cope with slow glucose influx. These results suggested that slow glucose import rates mimicked the reduced glucokinase activity of the evolved strains, leading to a stable system. At the other extreme, high glucose concentrations (111 mM) prevented growth and, as demonstrated above in the *Yl-*Glycolysis and *Yl-*3K strains, required reduction of the glucokinase activity. At intermediate concentrations of 10 mM–50 mM, shorter lag phases of 20 to 60 hours were observed. Considering the measured growth rates, a single mutant cell present at the start of the culture would reach measurable biomass concentration after ca. 70 hours of growth for *Yl-*Glycolysis strain IMX2417, a value corresponding to the lag phase of cultures with 111 mM glucose, in which hexokinase mutations were systematically observed. The shorter lag phases measured at low glucose concentrations could therefore not be reasonably explained by the occurrence of mutations and outgrowth of the population by a mutant. Such a graded response to glucose concentration, reminiscent of *tps1* mutants (22), suggested population heterogeneity, with an increasing fraction of the population unable to grow due to glycolytic imbalance with increasing glucose concentration.

### Removal of allosteric regulation of *S. cerevisiae* glycolytic kinases causes bistability upon sugar transition

**Fig 7:**
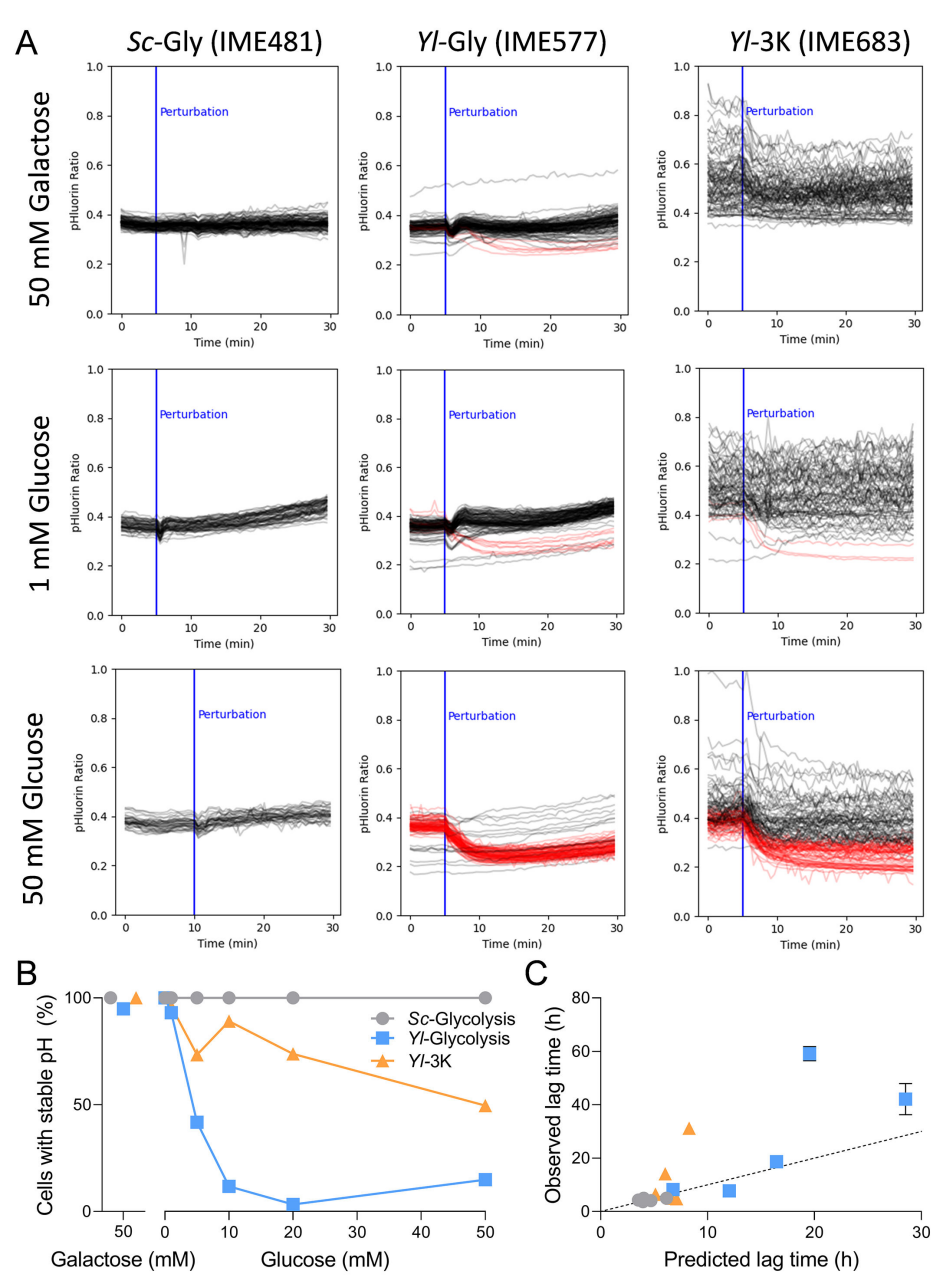
Single-cell metabolic response of *Sc*-Gly (IME481), *Yl*-Gly (IME577), and *Yl*-3K (IME683) to transition to galactose and glucose at various concentrations, as reported by pHluorin. (**A**) Single-cell profile of the pHluorin ratio in time upon addition of 50 mM galactose (top row), 1 mM (middle row), and 50 mM glucose (bottom row) to cells suspended in medium without a carbon source. Black profiles indicate cells with stable intracellular pH, while red indicates cells that display unstable intracellular pH upon sugar addition. Cells with pHluorin ratio above 0.3 at t = 0 and below 0.3 10 min after perturbation were considered unstable. (**B**) Fraction of cells with stable pH as a function of glucose concentration. On average, 98 ± 33 cells were counted for each data point. The complete data set is available in Fig. S13. (**C**) Measured lag time (data from Fig. 6B) is plotted against the lag time predicted from the fraction of stable cells shown in panel B. The average of triplicates and standard deviation are shown for the recorded lag time. The identity line is plotted as a dashed line.

To explore the possibility of bistable yeast populations triggered by sugar transition, cellular metabolic responses were monitored at single-cell levels using cell tracking by microscopy and pH_i_ as a proxy for metabolic status. The pHluorin ratio, reflective of the pH_i_ of the strains *Sc*-Gly (IME481), *Yl*-Gly (IME577), and *Yl*-3K (IME683), was monitored before and after addition of 50 mM galactose (negative control) and of glucose concentrations ranging from 1 to 50 mM, to cells in medium devoid of a carbon source. In the absence of sugar or with galactose addition, the pH_i_ remained stable (pHluorin ratio between 0.3 and 0.4) during the whole experiment (30 min) for all cells in the population of all three tested strains (Fig. 7A; Fig. S13). The three strains responded very differently, however, to glucose gradients. As expected, all cells in the population of IME481 with *S. cerevisiae* glycolysis were able to maintain their pH_i_ irrespective of the concentration of glucose (Fig. 7A; Fig. S13 to S15). Conversely, both IME577 and IME683 with full or partial *Y. lipolytica* glycolysis displayed bistability, with the fraction of cells with unstable pHi positively correlated with glucose concentration (Fig. 7B; Fig. S13 to S15). The phenotype was more pronounced for IME577 with full *Y. lipolytica* glycolysis than for the *Yl*-3K population, a result in good agreement with the difference in lag phase measured at the whole-population level for these two strains (Fig. 6). After the addition of 50 mM glucose to sugar-depleted medium, only 15% of the *Yl*-Gly (IME577) population was metabolically active and maintained its pH_i_. The *Yl*-3K (IME683) population appeared more robust to high glucose concentrations, with 50% of the population maintaining its pH_i_ with 50 mM glucose (Fig. 7A and B). Assuming that population heterogeneity is responsible for the lag phase of the *Yl*-Gly and *Yl*-3K strains, the fraction of cells maintaining metabolic activity should give a rough prediction of lag-phase duration. Accordingly, measured and predicted lag-phase durations showed similar trends for the *Yl*-Gly and *Yl*-3K strains (Fig. 7C). The discrepancy between measured and predicted lag-phase duration might indicate that the ability of cells to maintain pH_i_ is not necessarily predictive of their ability to divide. The presence or absence of regulated kinases strongly altered the cells’ ability to cope with high extracellular glucose concentrations and the resulting high intracellular glucose fluxes.

## DISCUSSION

### The limits of single complementation to study metabolic regulation

While metabolic control theory has long suggested that modification of the activity of a single enzyme (by expression or regulation) is usually not sufficient to greatly change pathway flux (28, 29), single-enzyme studies are still routinely performed. The present work illustrates that, despite technical challenges, the complexity of biology requires experimental investigation at the larger scale of pathway or function. The EMP pathway of glycolysis, widely distributed across kingdoms, is characterized by a structure in which ATP is first invested in the top of the pathway before it can be recouped further downstream. Such a configuration is prone to imbalance when cells have to transition between conditions poor and rich in hexoses (7), a problem for which various solutions have emerged. Organisms such as the parasite *Trypanosoma* control ATP use by glycolysis by sequestering glycolytic enzymes in specialized compartments called glycosomes (30, 31). In many prokaryotes, hexose uptake and phosphorylation are coupled to lower glycolysis via the phosphotransferase system (32, 33), while many eukaryotes are equipped with hexose-phosphorylating enzymes inhibited by T6P or G6P (34, 35) that apparently keep upper glycolytic flux in check during fluctuating substrate supply. However, all studies reporting the complementation of *S. cerevisiae* Hxk2 by insensitive variants suggest that Hxk2 inhibition by T6P is not by itself essential for the regulation of the glycolytic flux (10, 36). The same holds for pyruvate kinase and its activation by F1,6bP (11), and phosphofructokinase and its myriad of metabolic regulations (12, 13, 37). This insensitivity of yeast physiology to alterations in glycolytic activity and allosteric regulation has been the subject of debate for decades (38–41). Similarly, in prokaryotic model organisms, while overexpression and deregulation of phosphofructokinase and pyruvate kinase had some minor effects on flux distribution and intracellular metabolites, tight regulation of these reactions appears far from essential (42–44). The widespread nature of allosteric regulation of several key glycolytic enzymes raises the question of the function and essentiality of these mechanisms. By combining the expression of multiple unregulated enzymes in *S. cerevisiae* glycolysis, the present study explored the essentiality of allosteric regulation using novel synthetic biology methods. Remarkable differences in phenotype were observed between *S. cerevisiae* strains expressing single, triple, or the complete set of *Yarrowia* glycolytic enzymes. While it was confirmed that individual expression of insensitive enzyme variants could lead to functional complementation in *S. cerevisiae*, the combination of three insensitive kinases did not. This study, therefore, identifies for the first time the essential but redundant functions of these metabolic regulations for allowing transitioning of *S. cerevisiae* between low-sugar and high-sugar environments. The results strongly suggest that allosteric regulations in glycolysis, while not required for pathway operation *per se*, have been subject to strong evolutionary selection as they allow efficient and balanced pathway operation under dynamic conditions. The *Yl-*Glycolysis strains generated in this study have potential future applications in the study of adaptation to deregulation of essential pathways by further evolution and metabolite analysis. It can be expected that evolution would increase the glycolytic flux, but whether regulatory mechanisms will develop in short timescales to increase robustness would be interesting.

### Mutations in *Y. lipolytica* glucokinase reduce glycolytic flux and act as a suppressor for a deregulated glycolysis

Replacement of *S. cerevisiae* by *Y. lipolytica* glycolysis resulted in a phenotype strongly suggesting glycolytic bistability. Single, double, and triple kinase mutants and kinetic modeling demonstrated a strong synergistic role of hexokinase, phosphofructokinase, and pyruvate kinase in transitioning between glucose-poor and glucose-rich medium. Single-cell analysis revealed the presence of two states in the yeast population, a fraction able to cope with glucose inflow with a balanced glycolysis, and a fraction trapped in an imbalanced state. Such a stochastic effect, with a fraction of the population behaving differently from the majority, has previously been observed in yeast and other organisms during transition between carbon sources (22, 45, 46). Remarkably, the loss of these complex, synergistic regulations could be easily fixed by a single mutation of glucokinase. The six characterized mutants carried different mutations of the *YlGLK1* gene, with a common feature of a strong reduction in the *in vitro* glucose-phosphorylating activity and concomitant low glucose uptake rate. A previous study demonstrated that functional complementation with the G6P-sensitive human HK1 and HK2 in *S. cerevisiae* also required single mutations in these hexokinases for growth on glucose (16). These mutations were, however, clustered in a specific region of the protein, leading to the alleviation of G6P inhibition. *Yarrowia* glucokinase has no known effectors, and the random localization of the amino acid substitutions suggests that protein domains may not be relevant, as long as the substitutions enable the reduction of the glucose-phosphorylating activity. This “simple solution” comes at a cost, however, shown by the more than twofold reduced glucose uptake rate of the *Yl-*3K strain as compared to the reference *S. cerevisiae* strain (Fig. 5G), while the specific activity of PFK and PYK was not expected to be different between the two strains (Fig. 2G). Thus, while constantly imposing a slow flux in the top part of glycolysis allowed growth without defect under dynamic conditions, the maximal growth rate was limited. Both evolved and unevolved *Yl-*3K strains would likely be rapidly outcompeted by the reference *S. cerevisiae* strain with native regulations, which is capable of high-glucose uptake rates and efficient transition to high-glucose conditions. The evolved *Yl-*3K strains are, however, fantastic testbeds to explore the evolution of regulatory mechanisms, for instance, by long-term exposure to sugar transitions in adaptive laboratory evolution experiments. The strains constructed in this study also enable exploration of the role of the regulatory synergy between the three kinases in dynamic situations beyond sugar transitions. This could be achieved by growing the evolved and non-evolved *Yl-*3K strains and strains with single kinase complementation in a broad range of dynamic growth conditions.

### How does *Yarrowia lipolytica* cope with high hexose concentrations?

Replacement of the key regulatory enzymes in glycolysis with *Y. lipolytica* variants led to a dysfunctional pathway in *S. cerevisiae* during growth on glucose, unless the flux was reduced. The EMP pathway in *Y. lipolytica* has the same set of reactions, and therefore the same intrinsic risk of metabolic imbalance as its distant relative *S. cerevisiae*, yet *Y. lipolytica* is capable of fast growth on media with high glucose concentrations (around 0.37 h^−1^ with over 55 mM of glucose [47]). The mechanisms enabling *Y. lipolytica* glycolysis to operate in its native context are therefore intriguing and most probably find their roots in the strong difference in ecological niches and lifestyle of these two yeasts. While *S. cerevisiae* thrives in hexose-rich environments and has a very limited carbon substrate range, *Y. lipolytica* is much more versatile and can use a broad range of carbon sources, such as glycerol, fatty acids, and hydrocarbons, but is more limited in its utilization of sugars (48–50). These nutritional preferences are likely reflected in the mechanisms controlling glycolysis. Two likely strategies that would enable *Yarrowia* to control the glycolytic flux without the stringent regulations on the regulatory key-point enzymes glucokinase, phosphofructokinase, and pyruvate kinase that seem to be essential in *S. cerevisiae* are (i) imposing a low flux in the first, ATP-consuming steps of glycolysis or (ii) strictly controlling extracellular glucose influx in the cells. In line with the first idea, *in vitro* glycolytic enzyme activities in *Yarrowia lipolytica* are far lower than those found in *S. cerevisiae*. This is especially the case for upper glycolysis (Fig. S16) with glucose phosphorylation activity four- to sixfold lower in *Y. lipolytica* as compared to *S. cerevisiae* (in line with previous studies [47]) and phosphofructokinase activity three- to fourfold lower (Fig. S16). This lower rate of glucose phosphorylation and glycolysis as a whole might be sufficient for *Y. lipolytica* to cope with glucose fluctuations in its environment without imbalance in the pathway, as observed in the glucokinase mutant *Yl-*Glycolysis strains. Additionally, transport might further contribute to maintaining a slow glucose influx. Several hexose transporters have been identified in *Y. lipolytica*; however, little is known about their sugar preference and kinetic properties. Whether import plays a role in controlling the glycolytic flux in *Y. lipolytica* can be explored by hexose transporter engineering, as performed earlier with *S. cerevisiae* (51–53). Alternatively, it would be interesting to reverse engineer *Y. lipolytica* with the evolved glucokinases with lower specific activities. Finally, considering the knowledge gap on *Y. lipolytica* lifestyle and glycolytic enzyme kinetic properties and regulation, we cannot rule out the possibility that some yet unidentified metabolites outside glycolysis are also involved in the regulation of the glycolytic flux in *Y. lipolytica*.

Our results reinforce the essentiality of metabolic regulation of fluxes in central metabolism, especially for high-flux pathways such as *S. cerevisiae* glycolysis, which are susceptible to metabolic imbalance. Whether this essentiality holds more generally for other metabolic pathways and other species remains to be seen, but the importance of a small number of central metabolites in the control of metabolism is well established (54, 55). A similar essential metabolic control mechanism can therefore be expected in unrelated species and pathways as well, especially if they contain ATP-consuming steps that might lead to cell-wide metabolic imbalances.

### Glycolysis swapping beyond *Yarrowia lipolytica*

Glycolysis, one of the most conserved pathways, is functionally replacable among a broad range of organisms across the kingdom of life. This is well illustrated by the present work and the earlier successful humanization of yeast glycolysis (16). This transportability, despite certain technical limitations such as the ability to finely tune expression of heterologous genes, offers a unique opportunity to address many fundamental questions about gene and protein regulation and interactions with the intracellular environment. It would be very interesting to express more distant pathways with divergent regulation, such as the glycolysis of *Lactococcus lactis* (although expression in yeast of a functional PTS is a challenge). Considering other bacteria, it is unfortunate that *S. cerevisiae* is, to date, incapable of functionally expressing 6-phosphogluconate dehydratase, which prevents the possibility to more accurately mimic glycolysis involving the Entner-Doudoroff pathway in yeast (56). The compartmentalized glycolysis of trypanosomes is also an attractive pathway for transplantation in yeast. Beyond glycolysis, the recent minimization of *S. cerevisiae*’s entire central carbon metabolism (57) opens up pathway swapping at the scale of entire central carbon metabolism. Such extensive metabolic remodeling exercises bring new questions regarding the metabolic environment after transplantation, more particularly to what extent it remains native in the host.

## MATERIALS AND METHODS

### Strains and cultivation conditions

All *S. cerevisiae* strains used are derived from the CEN.PK lineage (58) and are listed in Table S1. *Yarrowia lipolytica* strains W29 and CJM246 (PO1a) were used as controls for enzyme activity measurements. Yeast strains were grown on either YP medium containing 10 g L^−1^ Bacto Yeast Extract and 20 g L^−1^ Bacto Peptone or synthetic medium (SM) containing 5 g L^−1^ (NH_4_)_2_SO_4_, 3 g L^−1^ KH_2_PO_4_, 0.5 g L^−1^ MgSO_4_·7H_2_O, 1 mL L^−1^ of a trace element solution, and 1 mL of a vitamin solution added after autoclaving (59). For physiological characterization of the *Sc-*Glycolysis and evolved isolates of the *Yl-*Glycolysis and *Yl-*3K strains in shake flasks (Fig. 3F through I, and 5E through H), SMD-urea was used, where the (NH_4_)_2_SO_4_ was replaced with 6.6 g L^−1^ K_2_SO_4_ as the source of sulfate and 2.3 g L^−1^ urea. Similarly, when the dominant marker *amdS* was used, (NH_4_)_2_SO_4_ was replaced by 6.6 g L^−1^ K_2_SO_4_ and 1.8 g L^−1^ acetamide. The pH of SM was set to 6 prior to autoclaving by addition of KOH. Media were supplemented with relevant carbon sources after autoclaving; final concentrations were 20 g L^−1^ glucose (YPD/SMD), 20 g L^−1^ galactose (YP-Gal/SM-Gal), 2% (vol/vol) ethanol (YPE/SME), or 1% (vol/vol) ethanol and 1% (vol/vol) glycerol (YPEG/SMEG). For solid media, 2% (wt/vol) agar was added before heat sterilization. For counterselection of the *amdS* marker, 2.3 g L^−1^ fluoroacetamide was added to SM (60), and for selection on the *KanMX* and *hphNT1* markers, 200 mg L^−1^ G418 or 200 mg L^−1^ hygromycin was added to YP medium. Yeast cultures were grown at 30°C at 200 rpm in an Innova 44 incubator shaker (New Brunswick Scientific, Edison, NJ, USA) in 50, 100, or 500 mL shake flasks containing, respectively, 10, 20, or 100 mL of medium. For plasmid propagation and maintenance, *Escherichia coli* XL1-Blue cells (Agilent Technologies, Santa Clara, CA, USA) were used, grown in lysogeny broth medium containing 10 g L^−1^ tryptone, 5.0 g L^−1^ yeast extract, and 4 g L^−1^ NaCl, supplemented with 100 mg L^−1^ ampicillin, 25 mg L^−1^ chloramphenicol, or 50 mg L^−1^ kanamycin when required. Yeast and *E. coli* strains were stored at −80°C in 1 mL aliquots of appropriate medium after addition of 30% (vol/vol) glycerol.

### Molecular biology techniques

PCR amplification for strain construction purposes was performed using Phusion High-Fidelity DNA Polymerase (Thermo Fisher Scientific, Waltham, MA), according to the manufacturer’s recommendations, except that the primer concentration was lowered to 0.2 µM. Diagnostic PCR amplification was performed using the DreamTaq PCR Master Mix (Thermo Fisher Scientific) following the supplier’s recommendations. Primers for cloning purposes were ordered PAGE-purified; all other primers were ordered desalted (Sigma-Aldrich, St. Louis, MO, USA). To obtain double-stranded gRNA and repair fragments, the designed forward and reverse oligos, ordered as PAGE-purified primers, were incubated at 95°C for 5 min and allowed to cool to room temperature. PCR products were separated by gel electrophoresis with gels containing 1% agarose (TopVision Agarose, Thermo Fisher Scientific) in 1× Tris-acetate-EDTA buffer (Thermo Fisher Scientific); 10 µL L^−1^ SERVA (SERVA Electrophoresis GmbH, Heidelberg, Germany) was added to the gel for DNA staining. As a size standard, the GeneRuler DNA Ladder Mix (Sigma-Aldrich) was used. PCR fragments used for cloning obtained from plasmids were treated with the addition of 1 µL DpnI FastDigest restriction enzyme (Thermo Fisher Scientific) for 1 hour to remove remaining template DNA. DNA was purified using the GenElute PCR Clean-Up Kit (Sigma-Aldrich) or the GeneJET PCR Purification Kit (Thermo Fisher Scientific) when no unspecific bands were present; otherwise, products were purified from gel using the Zymoclean Gel DNA Recovery kit (Zymo Research, Irvine, CA). Purity and quantity of DNA were assessed using a NanoDrop 2000 spectrophotometer (Thermo Fisher Scientific). For more precise DNA quantification, the Qubit dsDNA BR Assay Kit (Thermo Fisher Scientific) in combination with the Qubit 2.0 Fluorometer (Invitrogen, Carlsbad, CA, USA) was used.

Cloning of promoters, genes, and terminators was done using Golden Gate assembly or *in vivo* assembly in yeast. For Golden Gate assembly, per reaction volume of 10 µL, 1 µL T4 buffer (Thermo Fisher Scientific), 0.5 µL T7 DNA ligase (New England Biolabs, Ipswich, MA), and 0.5 µL BsaI (Eco31I) (Thermo Fisher Scientific) or BsmBI (NEB) were used, and DNA parts were added in equimolar amounts of 20 fmol as previously described (61). *In vivo* assembly of plasmids in yeast was performed according to reference 62, using 60 bp homologous flanks added by PCR and transformation of all fragments to *S. cerevisiae*. After transformation and colony PCR verification of correct assembly, plasmids were purified using the Zymoprep Yeast Plasmid Miniprep II Kit (Zymo Research). Gibson assembly for construction of gRNA plasmids was performed with Gibson Assembly Master Mix 2× (New England Biolabs, Ipswich, MA) according to the manufacturer’s instructions but scaled down to a final volume of 5 µL.

Plasmids were transformed into *E. coli* XL1-Blue by chemical transformation for amplification (63). Plasmids were isolated using the GenElute Plasmid Miniprep Kit (Sigma-Aldrich) or the GeneJET Plasmid Miniprep Kit (Thermo Fisher Scientific) and verified by diagnostic PCR or restriction analysis using FastDigest restriction enzymes with FastDigest Green Buffer (Thermo Fisher Scientific) according to the manufacturer’s instructions. Transformation of *S. cerevisiae* was done with the lithium acetate/PEG/ssDNA method (64), and colonies were verified by diagnostic PCR. Single-colony isolates were obtained by three consecutive restreaks on selective solid medium. Yeast DNA was isolated by either boiling in 0.02 N NaOH, the protocol described by Lõoke et al. (65), or the QIAGEN Blood & Cell Culture Kit with 100/G Genomic-tips (Qiagen, Hilden, Germany), depending on the desired DNA purity.

### Plasmid and strain construction

#### Isolation of the *Yarrowia lipolytica* glycolytic genes and plasmid construction

Gene, cDNA, and protein sequences of the *Y. lipolytica* glycolysis were obtained from the Genome Resources for Yeast Chromosomes database (https://gryc.inrae.fr/). The genes *YlPGI1, YlFBA1, YlTPI1, YlTDH1, YlGPM1*, and *YlENO1* were identified based on sequence similarity to the *S. cerevisiae* glycolytic genes (Table 1). A *Yarrowia lipolytica* cDNA library from strain W29 (66), kindly provided by C.-L. Flores, was used for the cloning of glycolytic gene sequences, except the genes for *HXK1*, *GLK1*, *PFK1,* and *PYK1*, which were obtained codon-optimized for *S. cerevisiae* by GeneArt Gene Synthesis (Thermo Fisher Scientific). *Y. lipolytica* genes were amplified from the cDNA library using the primers listed in Table S4A and subsequently assembled into part plasmids using Golden Gate assembly with the pUD565 entry vector. Subsequently, the genes were assembled into transcriptional cassettes with *S. cerevisiae* promoters and terminators using Golden Gate assembly (*YlFBA1, YlPGK1*) or *in vivo* yeast assembly (*YlENO1, YlPGI1, YlTPI1, YlTDH1,* and *YlGPM1*); used promoters and terminators are shown in Table S2. For both methods, the backbone was obtained from plasmid pGGKd017 (67) and *S. cerevisiae* promoters previously assembled onto part plasmids (Table S3, [16]). For *in vivo* assembly, the pGGKd017 backbone and required *S. cerevisiae* promoter and terminator parts were amplified, as well as the *Y. lipolytica* genes, with 60 bp homologous regions. The genes that were synthesized codon-optimized (*YlHXK1, YlGLK1, YlPFK1,* and *YlPYK1*) were assembled using Golden Gate assembly directly with plasmid pGGKd002 and promoter and terminator part plasmids to obtain plasmids that could be integrated into the *S. cerevisiae* genome directly after linearization (Table S3). CRISPR-Cas9 yeast genetic engineering and construction of gRNA plasmids were performed as described (68).

**TABLE 1:** *Yarrowia lipolytica* glycolytic gene accession numbers

Gene	Previously functionally annotated	Accession number GRYC
YlGLK1	(19)	YALI0E15488g
YlHXK1	(18)	YALI0B22308g
YlPGI1	No	YALI0F07711g
YlPFK1	(12)	YALI0D16357g
YlFBA1	No	YALI0E26004g
YlTPI1	No	YALI0F05214g
YlTDH1	(69)	YALI0C06369g
YlPGK1	(70)	YALI0D12400g
YlGPM1	No	YALI0B02728g
YlENO1	No	YALI0F16819g
YlPYK1	(71)	YALI0F09185g

Similarly, to facilitate amplification of the native *S. cerevisiae* glycolytic gene cassettes, these were amplified from CEN.PK113-7D with their native promoter and terminator sequences and assembled into glycolytic expression cassettes using Golden Gate assembly with dropout vector pGGKd017 (Table S3).

#### Construction of a full *Y. lipolytica* glycolysis expression strain and *Sc*-Glycolysis reference strain

For strain construction, Cas9-expressing background strains were used to facilitate CRISPR-Cas9 genome editing according to reference 68. An overview of the most important genetic modifications and strain pedigree is shown in Fig. S17. To facilitate the construction of strains expressing various combinations of *Y. lipolytica* and *S. cerevisiae* glycolytic genes, first, the seven glycolytic genes excluding the key kinases (*YlPGI1, YlFBA1, YlTPI1, YlTDH1, YlPGK1, YlGPM1,* and *YlENO2*) were integrated in the *CAN1* locus of platform strain IMX1822 together with the *S. cerevisiae ADH1* and *PDC1* genes for alcoholic fermentation. These genes were amplified from their respective expression cassette plasmids flanked by *S. cerevisiae* promoters and terminators, and 60 base pair homology flanks were added during PCR to enable *in vivo* assembly (primers in Table S4B). A total of 200–350 ng of each repair fragment and 1 µg of gRNA plasmid pMEL13 targeting *CAN1* were used to transform SwYG strain IMX1822. The resulting strain was verified by diagnostic PCR to contain all integrated genes (diagnostic primers in Table S4E) and named IMX2065. Subsequently, the *E. coli* GroEL and GroES chaperone genes were integrated in the X2 locus, and the *URA3* marker gene was deleted to enable selection on uracil prototrophy. To this end, gRNA plasmid pUDR591 was constructed by Gibson assembly of the pROS13 backbone and a fragment containing the 2µm origin amplified by primers containing the gRNA sequences targeting X2 and *URA3* (primers in Table S4F). pUDR591 was co-transformed with the chaperone gene cassettes amplified from pUDE232 and pUDE233 and a repair fragment for the *URA3* locus. After selection on YPD-G418, this resulted in strain IMX2151. In this strain, the *Y. lipolytica* glycolytic kinases *YlGLK1, YlPFK1,* and *YlPYK1* were integrated in the *SPR3* locus by transformation of the three expression cassettes amplified from pUDI226 to pUDI228 and the gRNA plasmid pUDR596 targeting the *SPR3* locus. pUDR596 was constructed by Gibson assembly of the pROS10 plasmid backbone with a fragment containing the 2µm origin flanked by the *SPR3* gRNA sequence (Table S4F). The gRNA plasmid was removed by non-selective growth, and the uracil-auxotrophic strain was stocked as IMX2333.

To remove the native *S. cerevisiae* glycolytic genes and the *AmdS* marker from the *sga1* locus, IMX2333 was transformed with recycle plasmid pUDE342 and a repair fragment (counter-select oligo) and selected on SM-EtOH supplemented with fluoroacetamide to select against the presence of the *AmdS* marker in the *sga1* locus. After PCR verification and removal of the gRNA plasmid, the strain, which only carries *Y. lipolytica* glycolytic genes, was stocked as IMX2363. This strain was verified by whole-genome sequencing. In this strain, the *ura3-52* locus was repaired to generate a prototrophic strain for physiological characterization by transformation of a *URA3* gene cassette amplified from plasmid pYTK074. The correct integration of *URA3* was verified by Sanger sequencing of the amplified *URA3* locus, and the resulting strain was named IMX2417.

To generate a comparable control strain with the native *S. cerevisiae* glycolytic genes, the *E. coli* GroEL/ES genes were integrated into strain IMX1821, which carries the native glycolytic genes in the *can1* locus. The chaperone genes were integrated into the X2 locus by co-transformation of gRNA plasmid pUDR547 and the GroEL/ES expression cassettes and selection on hygromycin, leading to strain IMX2696, which was similarly verified by diagnostic PCR.

### Construction of mosaic glycolysis and single complementation strains

To verify the function of the *Y. lipolytica* glycolytic genes, several strains with combinations of the *Y. lipolytica* and *S. cerevisiae* glycolysis genes were constructed. Single complementation strains expressing the *YlHXK, YlGLK, YlPFK,* and *YlPYK* were constructed by transformation of 750 ng of the plasmids pUDI225–pUDI228, linearized by NotI digestion (FastDigest, Thermo Fisher Scientific) according to the manufacturer’s protocol, to uracil-auxotrophic MG strain IMX1076 (16). The linear plasmids were integrated into the disrupted *ura3-52* locus, and strains were selected on SMD medium. Integration was verified by diagnostic PCR. In the resulting strains (IMX2047–IMX2050), the native yeast glycolytic genes were subsequently removed in a second round of transformation using CRISPR-Cas9. *HXK2* was deleted by transformation with pUDR371, *PFK1* and *PFK2* were deleted by transformation with pUDR265, and *PYK1* was deleted by transformation with the pMEL13 backbone and the *PYK1* gRNA for *in vivo* assembly. Cells were plated and restreaked on YP-ethanol supplemented with G418 to avoid selection for mutations allowing growth on glucose. This resulted in strains IMX2061 (*YlHXK* complementation), IMX2062 (*YlGLK* complementation), IMX2236 (*YlPFK* complementation), and IMX2235 (*YlPYK* complementation). Strains IMX2236 and IMX2235 were verified by whole-genome sequencing.

To construct a double *YlGLK-YlPYK* complementation strain, the *YlPYK* complementation strain IMX2235 was transformed with *HXK2* gRNA plasmid pUDR371 and *HXK2* deletion repair fragments to delete the native *HXK2* gene, generating strain IMX2812 after selection on YP-Gal supplemented with G418. Subsequently, in this strain, the *YlGLK1* gene was integrated in the X2 locus by transformation with gRNA plasmid pUDR547, targeting the X2 locus, and a repair fragment containing the *YlGLK1* gene flanked with homology flanks to the X2 locus. After selection on YP-Gal with hygromycin, verification by PCR, and restreaking, the strain was stocked as IMX2842.

To make the *Sc-*3K strain, the *S. cerevisiae* kinases *ScHXK2, ScPFK1, ScPFK2,* and *ScPYK1* and the seven *Y*. *lipolytica* genes *YlPGI1, YlPGK1, YlTDH1, YlENO1, YlGPM1, YlGPM1, YlGPM1,* and *YlTPI1* were integrated in one transformation step in the *CAN1* locus of SwYG strain IMX589. To this end, the *Yl* glycolytic gene cassettes were amplified from the expression cassette plasmids with homology flanks as before, but with some modifications to the flanks to incorporate the *S. cerevisiae* gene cassettes (primers in Table S4B and C). The fragments were co-transformed with the *URA3*-containing plasmid pMEL10, and the resulting strain was selected on SM-ethanol and named IMX1751. In this strain, the native glycolytic genes were removed from the *sga1* locus by transformation with the recycle plasmid pUDE342 and selection on SM-ethanol-fluoroacetamide, leading to strain IMX1803, which was verified by whole-genome sequencing. This strain was subsequently transformed with a *URA3* gene fragment to repair the *URA3* locus and generate a prototrophic strain as described above, leading to strain IMX2465. To make this strain directly comparable to the fully swapped glycolysis strain, the *E. coli* chaperones GroEL and GroES were integrated in the *X2* locus by co-transformation of the GroEL and GroES expression cassettes and X2 gRNA plasmid pUDR547, leading to strain IMX2697.

Similarly, a pathway with the *Yarrowia lipolytica* kinases *YlGLK*, *YlPFK,* and *YlPYK* and seven *S*. *cerevisiae* genes was designed. As a background strain, the *E. coli* chaperones GroEL and GroES were integrated first into SwYG strain IMX589, again by co-transformation of pUDR547 and the chaperone expression cassettes, leading to strain IMX2694. Integration of the mosaic glycolytic pathway in this strain was done by co-transformation of the expression cassettes with pMEL13 targeting the *CAN1* locus. Selection was performed on YP-Gal supplemented with G418, which led to strain IMX2703. Deletion of the native glycolytic genes from *sga1* in the same manner described above, with selection on SM-Gal-fluoroacetamide, led to strain IMX2718. In this strain, the *ura3* locus was repaired in the same manner described above to obtain a uracil-prototrophic strain, named IMX2733, which was also described as the *Yl-*3K strain.

Finally, a strain with the full *Yarrowia* glycolysis, except the phosphofructokinase, was constructed, starting from intermediate strain IMX2151, which carries the seven *Yarrowia* glycolytic genes excluding the three key kinases, and the GroES and GroEL chaperone genes. Similar to the construction of the full *Yarrowia* glycolysis strain, expression cassettes for the key points, *YlGLK1, ScPFK1, ScPFK2,* and *YlPYK1*, were integrated into the *SPR3* locus. Cassettes were amplified from pUDI226 (*YlGLK1*), pUDE769 (*ScPFK1*), pUDE770 (*ScPFK2*), and pUDI228 (*YlPYK1*), and flanks were adapted to allow *in vivo* assembly of these four genes in the *SPR3* locus. Transformation with gRNA plasmid pUDR596 and the expression cassettes resulted in strain IMX2164 after PCR verification and single-colony isolation. From this strain, the native glycolytic genes were removed, as described above, by co-transformation of gRNA plasmid pUDE342 and repair fragments with homology to the *SGA1* locus, resulting in strain IMX2182 after selection on SM-EtOH and counterselection of the AmdS marker on fluoroacetamide. This strain was verified by whole-genome sequencing. To generate a prototrophic strain, the *URA3* marker was repaired by transformation with the *URA3* fragment amplified from pYTK074 and selection on SM-galactose.

#### Construction *tps1* strain

To verify the function of pHluorin, we constructed a *tps1* deletion strain in the CEN.PK113-7D background. To enable deletion of *TPS1,* we constructed gRNA plasmid pUDR626 using Gibson assembly with the pMEL13 backbone and a double-stranded *TPS1* gRNA fragment (Table S4F). This plasmid was transformed into the Cas9-expressing strain IMX581 together with a double-stranded repair fragment and selected on YP-Gal-G418, and the deletion was verified by PCR. The resulting strain was stocked as IMX2243.

#### Construction of pHluorin-expressing strains

To enable estimation of the intracellular pH, the plasmid pYES2-*P_ACT1_*-pHluorin (24), which was kindly shared by Bas Teusink, was transformed into several uracil-auxotrophic strains (Table 2). Selection was performed in each case on SM-galactose or SM-ethanol medium, and presence of the plasmid was verified by observation of fluorescence. For the single complementation strains, the *URA3* marker was deleted to allow transformation of this plasmid by transformation with *URA3* gRNA plasmid pUDR107 and selection on YP-galactose-hygromycin, generating strains IMX2549–IMX2551.

**TABLE 2:** pHluorin-expressing strains^*a*^

Strain characteristic genotype	Uracil-auxotrophic host strain	pHluorin-expressing strain
Control strain	CEN.PK113-5D	IME480
*Sc*-Glycolysis	IMX589	IME481
*tps1* control strain	IMX2243	IME576
Full *Yl*-Glycolysis	IMX2363	IME577
*Sc-*3K strain	IMX1803	IME579
*Yl-*3K strain	IMX2718	IME683
*YlHXK* complementation strain	IMX2549	IME627
*YlGLK* complementation strain	IMX2550	IME628
*YlPFK* complementation strain	IMX2552	IME631
*YlPYK* complementation strain	IMX2551	IME632
*Yl-*Glycolysis *ScPFK*	IMX2182	IME609

^*a*^ Uracil-auxotrophic strains with various genotypes were transformed with plasmid pYES2-P_ACT1_-pHluorin; host and resulting strain are indicated.

### Growth rate and lag-phase determination and adaptive evolution

#### Growth profiler

For growth rate and lag-phase measurements, growth cultures were grown at 30°C and 250 rpm using a Growth Profiler 960 (EnzyScreen BV, Heemstede, The Netherlands). Strains were inoculated from glycerol freezer stocks and grown overnight in 10 mL YP-Gal medium in a shake flask. These cultures were transferred to 20 mL SM-Gal medium, which was grown until mid-exponential growth. From this culture, cells were re-suspended in SM without added carbon source and inoculated in 96-well square-well microtiter plates (EnzyScreen, type CR1496dl or CR1496dg), pre-filled with appropriate media, with final working volumes of 250 μL to a starting OD_660_ of 0.2. Microtiter plates were closed with a sandwich cover (EnzyScreen, type CR1296). Images of cultures were made at 20 min intervals. Green values for each well were corrected for the position in the plate using measurements of a culture of OD_660_ of 5 of control strain CEN.PK113-7D. Corrected green values were converted to OD values based on calibration measurements with the control strain CEN.PK113-7D, fitted with the following equation: OD-equivalent = a × GV(t) + b × GV(t)^c^ − d, in which GV(t) is the corrected green value measured in a well at time point “t.” This resulted in curves with the following values for a, b, c, and d: 0.07742; 1.662 * 10^-7^; 3.624; −1.615 for plates of the CR1496dl type, and 0.09622; 5.968 * 10^-6^; 3.254; −0.7939 for plates of the CR1496dg type. Growth rates were calculated in a time frame where the calculated OD was between 1 and 10 in which OD doubled at least twice except for the low-glucose experiments (Fig. 5), where cell densities remained low. Linear regression of the log-transformed OD data versus time was used to determine the growth rate. Lag time was defined as the time required to increase by an OD_660_ value of 0.6. This value was chosen because it was the lowest value that could be clearly distinguished from background noise for all experiments performed. Prediction and interpretation of lag times assumed exponential growth (Biomass(t) = Biomass(0) * exp(µ * t)).

#### Shake flask growth characterization

Growth rates and extracellular metabolite consumption and production were estimated from duplicate 100 mL shake flask cultures on SMD-urea. OD_660_ was measured on a JENWAY 7200 spectrophotometer (Cole-Parmer, Stone, UK). Wake-up cultures were inoculated in 10 mL YP-Gal medium and grown overnight. From there, pre-cultures were inoculated in 20 mL SMD and grown until exponential phase and transferred to SMD-urea. Samples were taken, and OD_660_ was measured, and 1 mL samples were centrifuged for 5 min at 20,000 *g* for extracellular metabolite determination. The supernatants were analyzed using an Aminex HPX-87 ion-exchange column operated at a 60°C and a flow rate of 0.6 mL/min with 5 mM H_2_SO_4_ as mobile phase (Agilent). Biomass dry weights were estimated from a correlation with dry weights measured on filters with pore size 0.45 µm as described previously (59). Growth rates were determined by linear regression on log-linear OD_660_ data over at least six consecutive points, over which the optical density doubled twice. The optimal range was chosen by maximization of the *R*^2^. Molar yields were estimated as the slope of the product concentration versus glucose concentration during the exponential phase. The specific substrate uptake rate was estimated by dividing the growth rate by the biomass yield on glucose. Specific ethanol production was estimated by multiplying the molar yield with the specific glucose uptake rate.

#### Shake flask adaptive evolution

Tests for adaptation on glucose medium of strains IMX2417, IMX2733, and IMX2062 were performed in shake flasks. Pre-cultures were inoculated in 20 mL non-selective YP-Gal medium and grown overnight. From there, cultures were transferred to 20 mL SM-Gal and grown until exponential phase. Exponential SM-Gal cultures were inoculated in triplicate 100 mL SMD cultures to a starting OD_660_ of 0.2. After growth on glucose-containing medium, cultures were transferred to 100 mL SM-Gal cultures, again to a starting OD_660_ of 0.2. After growth on SM-Gal, cultures were re-inoculated at OD_660_ 0.2 on SMD medium to verify whether a lag phase was still present. From each of these SMD cultures, single colonies were isolated by triplicate restreaking on solid SMD medium. A single isolate of each of the three shake flasks was stocked for each experiment, resulting in strains IMS1203, IMS1204, and IMS1205 from IMX2417; IMS1207, IMS1208, and IMS1209 from IMX2733; and IMS1218, IMS1219, and IMS1220 from IMX2062.

### Sequencing

High-quality genomic DNA was isolated with the QIAGEN Blood & Cell Culture Kit with 100/G Genomic-tips (Qiagen) from strains IMX2363, IMX2182, and IMX1803 and sequenced in-house using an Illumina MiSeq Sequencer (Illumina, San Diego, CA) as described previously (72, 73). For IMS1202, IMS1203, and IMS1204, DNA was obtained in the same manner but sequenced at NovoGene (NovoGene, Leiden, The Netherlands).

A *de novo* assembled reference genome was previously constructed for IMX589 (auxotrophic SwYG) using MinION and MiSeq data. Using the Burrows-Wheeler Alignment (BWA) Tool (74) (version 0.7.15), sequencing data of SwYG-derived strains (IMX2363, IMX2182, IMX1803, IMS1202, IMS1203, and IMS1204) was aligned to the IMX589 reference genome, and sequencing data of all strains was additionally aligned to a CEN.PK113-7D reference (75). The data were further processed using SAMTools (74) (version 1.3.1), and single nucleotide polymorphisms (SNPs) were determined using Pilon (with -vcf setting; version 1.18)) (76). The BWA.bam output file was visualized using the Integrative Genomics Viewer (version 2.4.0) (77), and copy numbers were estimated using Magnolya (version 0.15) (78). SNPs were compared between previously obtained sequencing data of parental SwYG strains IMX589 and IMX605 (14) and the SwYG-derived strains to verify the absence of mutations during strain construction and between the unevolved *Yl*-Glycolysis strain IMX2363 and evolved strains IMS1202, IMS1203, and IMS1204 to find mutations after growth on glucose (Fig. 3C and D). Sanger sequencing was performed at Baseclear BV (Baseclear, Leiden, The Netherlands) and Macrogen (Macrogen Europe, Amsterdam, The Netherlands). PCR-amplified fragments of the *YlGLK1* gene were sequenced from *Yl-*3K strains IMS1207, IMS1208, and IMS1209 and *YlGLK* complementation strains IMS1218, IMS1219, and IMS1220 with the primers listed in Table S4E.

### pHluorin pH_i_ determinations

pHluorin pH_i_ response was verified by measurement of the fluorescence signal in control strain IME480 after permeabilization by incubation with digitonin in Citrate-Na_2_PO_4_ buffers with known pH, as in reference 24, in a TECAN Infinite M200 Pro microtiter plate reader (TECAN, Männedorf, Switzerland) (Fig. S12). Flow cytometry for pHluorin fluorescence ratios was performed on a BD FACSCelesta (Becton Dickinson Biosciences, Breda, The Netherlands). Excitation was by a 405 nm laser (Violet) and a 488 nm laser (Blue), and emission was detected through BD Horizon Brilliant Violet 510 (525/50 nm) filter and a BD Horizon Brilliant Blue 515, FITC (530/30 nm) filter. FlowJo v.10 (BD Biosciences) was used to analyze and visualize FACS data. pHluorin-expressing strains and the CEN.PK113-7D non-fluorescent control strain were inoculated from freezer stocks in SM-Gal medium. These cultures were transferred to 15 mL SM-Gal, which was grown overnight to mid-exponential phase. Exponential cultures were harvested by centrifugation at 5,000 *g* and washed in 10 mL SM without C-source. Cultures were diluted to an OD_660_ of 0.5, and 260 µL aliquots were placed in round-bottom 96-well microtiter plates. Glucose or galactose was added to a final concentration of 20 g L^−1^. For the time-course measurements, there was approximately 1 min between addition of sugar and start of the measurement. The control strains IME480 and IME481 and the *tps1*Δ strain IME576 were first tested for their pH_i_ response (Fig. S12). At least 20,000 events were measured for each condition; fluorescent cells were gated based on fluorescence in both channels by comparing with the non-fluorescent control strain. Events on the edges (maximum detectable fluorescence) were removed to avoid skewing the ratio. Settings and voltages were kept the same for all experiments to verify reproducible ratios.

### Cell-free extract preparation and enzyme assays

*S. cerevisiae* samples for enzyme activity determinations were prepared as previously described (79), from exponentially growing cultures (approx. 62 mg dry weight per sample) from shake flask. For *Yarrowia lipolytica* (Fig. S16), a similar procedure was followed, but approximately double the amount of biomass was sampled (based on OD_660_). All determinations were performed at 30°C and 340 nm (εNAD(P)H at 340 nm/6.33 mM^−1^). Glycolytic *V*_max_ enzyme activities were determined in 1 mL reaction volume in 2 mL cuvettes, using a Hitachi model 100-60 spectrophotometer, using previously described assays (80), except for phosphofructokinase activity, which was determined according to Cruz et al. (81). *Y. lipolytica* glucokinase activity was assayed with increased glucose and ATP concentrations in strains IMX2417, IMX2733, IMS1202, IMS1203, IMS1204, IMS1207, IMS1208, and IMS1209 (Fig. S5). The reported data are based on at least two independent biological replicate samples, with at least two analytic replicates per sample per assay with different cell-free extract concentrations except the measurements at higher ATP concentrations (where no effect was seen). The protein concentration was determined using the Lowry method with bovine serum albumin as a standard (82). Enzyme activities are expressed as μmol substrate converted (mg protein)^−1^ h^−1^.

### Kinetic modeling of glycolysis

The kinetic model of yeast glycolysis of Van Heerden et al. (22) was obtained from jjj.bio.vu.nl/models/vanheerden1 and imported as a system of ordinary differential equations in Python using PySCeS (83). The system of ODEs was solved using the solve_ivp function in Python 3.6. Adaptations to the rate equations were made as follows. Removal of trehalose-6-phosphate inhibition of hexokinase was performed by removing the G6P inhibition term $\frac{\text{G6P}}{{\text{K}}_{\text{i,G6P}}}$ in the hexokinase rate equation, since T6P inhibition is modeled as G6P inhibition. ATP inhibition of phosphofructokinase was removed by changing parameter C_i_,ATP from 100 to 1. AMP activation of PFK was removed by changing parameter C_i_,AMP from 0.0845 to 1. Constant activation by fructose-2,6-bisphosphate of PFK was modeled by increasing the F2,6bP concentration from 0.02 to 0.1. Fructose-1,6-bisphosphate activation on PYK was removed by removing the F1,6bP activation term $\frac{\text{F16bP}}{{\text{K}}_{\text{m,F16bP}}}$ from the pyruvate kinase rate equation.

The various model configurations were solved with initial phosphate and FBP concentrations ranging between 0 and 20 and 0 and 5, respectively. One hundred different concentrations were run for each metabolite, resulting in 10,000 initial conditions for each model configuration. Steady state was evaluated after 100 min by checking if the FBP concentration changed more than 1% of its original initial concentration over the last five simulated time points.

### Single-cell analysis of pH_i_

pHluorin sugar pulse measurements were performed similar to reference 84. Strains IME481, IME577, and IME683 were pre-grown in SM-galactose to mid-exponential phase, washed with SM buffer without added sugar, and cell concentrations were standardized.

Prior to microscopy, cells were transferred to an Attofluor cell chamber (Thermo Fisher) containing a Concanavalin A precoated coverslip, prepared as described in reference 85, and incubated at 30°C for 30 min. Cells were imaged at 30°C using a Nikon Ti Eclipse widefield fluorescence microscope (Nikon, Tokyo, Japan) with an Andor Zyla 5.5 sCMOS camera (Andor) and a SOLA 6-LCR-SB power source (Lumencor, Beaverton, USA).

Fluorescence was measured with excitation filters at 400/40 nm as well as 480/40 nm and 505 nm long-pass dichroic and 535/50 nm emission filters (Semrock, Lake Forest, USA). Imaging was done with a Plan Apo λ 100× oil objective (N.A. 1.45) with an exposure time of 50 ms. A total of 4 × 4 hardware binning was used to acquire images in the fluorescent channels. Cell segmentation was performed for each time point on the brightfield images using a custom in-house pipeline using convolutional networks and used to track fluorescence intensity and ratio for each imaged cell. After 5 or 10 min, a glucose or galactose solution or water was added to a final concentration of 1, 5, 10, 20, or 50 mM. Data were filtered on cells for which data were available for the entire time course of 30 min and an average fluorescence ratio in a range consistent with live cells (below 0.7). Cells were classed as unstable if the fluorescence ratio dropped below a ratio of 0.3 at 10 min after perturbation.

## Data Availability

Raw Illumina sequence data from strains IMX2363, IMX2182, IMX1803, IMS1202, IMS1203, and IMS1204 are available at NCBI under Bioproject number PRJNA811750. Raw data and code used for the kinetic modeling of glycolysis and microfluidics analysis (Fig. 6 and 7) are deposited at the 4TU.ResearchData repository under DOI: 10.4121/79f009a8-f098-4135-ab03-fc3c97c7ef19.

## SUPPLEMENTAL MATERIAL

Supplemental figures and tables mbio.00219-25-s0001.pdf Fig. S1 to S17; Tables S1 to S4.

ASM does not own the copyrights to Supplemental Material that may be linked to, or accessed through, an article. The authors have granted ASM a non-exclusive, world-wide license to publish the Supplemental Material files. Please contact the corresponding author directly for reuse.
