# Supplementary material for: Synergistic regulatory mechanisms in glycolysis revealed by pathway transplantation
Source: mBio. 2025 Dec 5;17(1):e00219-25. doi: 10.1128/mbio.00219-25 (PMC12802204; doi:10.1128/mbio.00219-25)
Supplement: Supplemental figures and tables — Fig. S1 to S17; Tables S1 to S4. [file mbio.00219-25-s0001.pdf]

Supplementary data for the article

Synergistic regulatory mechanisms in glycolysis revealed by pathway transplantation

By Ewout Knibbe, Francine J. Boonekamp, Rachel Stuij, Philipp Savakis, Koen A. J. Pelsma, Liset Jansen, Carmen-Lisset Flores, Bas Teusink, Pascale Daran-Lapujade

Supplementary data

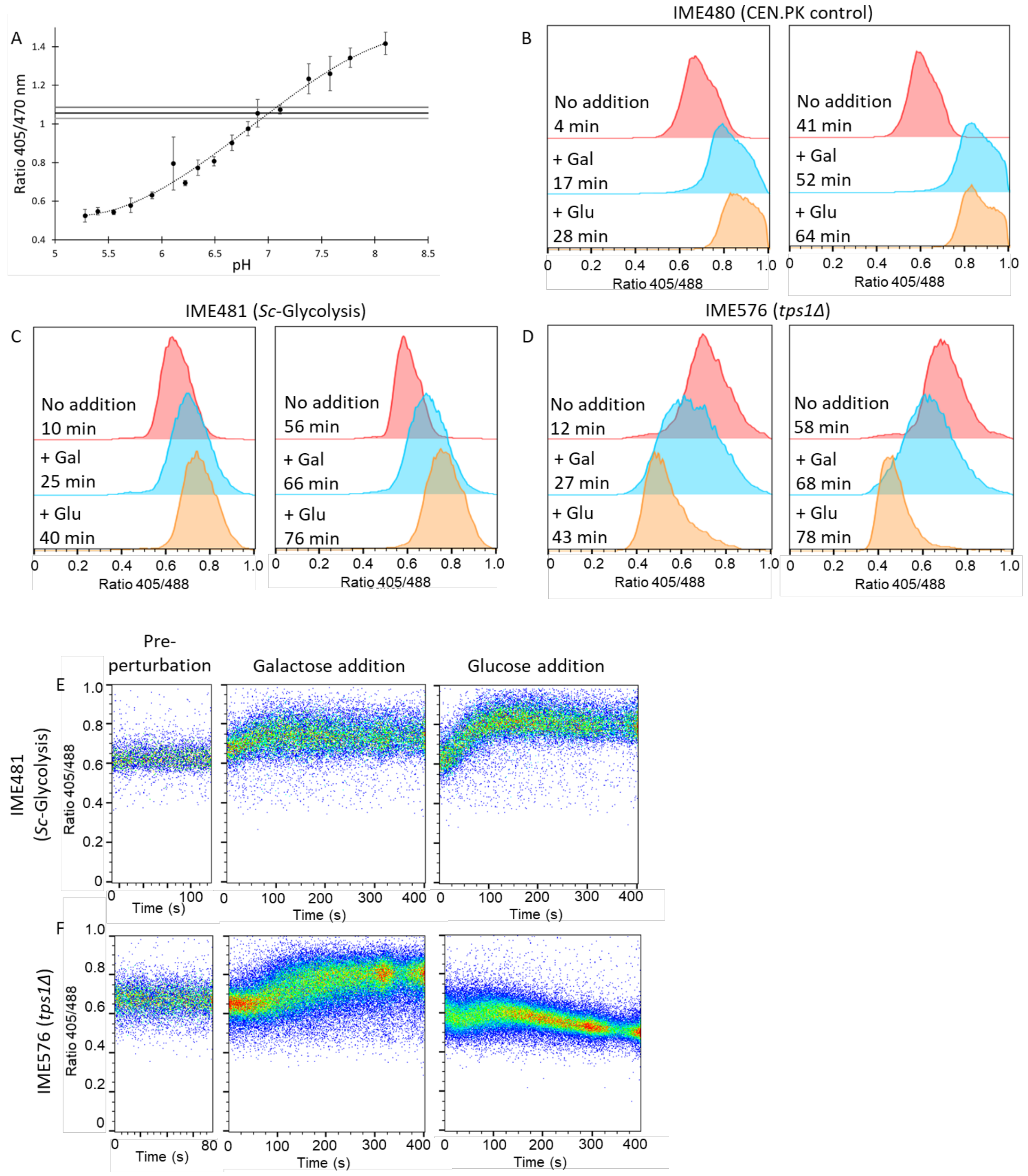

Figure S1 - Verification of pHluorin function.
Expression of pHluorin and its response to pH changes were verified. **A)** Strain IME480, a reference strain expressing pHluorin was incubated in presence of digitonin to permeate the cell membrane in Citrate-Na_2PO_4 buffers of known pH. The ratio of fluorescence intensity at 512 nm after excitation at 405 and 470 nm was determined. Triplicate wells were measured for each pH, mean and standard deviation are shown and a cubic trendline is plotted through the points. The horizontal line represents the signal measured in non-permeabilized cells in SM, grey lines indicate the standard deviation. **B)-D)** pHluorin signal in the fluorescent population of control strain IME480 (CEN.PK113-5D background), IME481 (SwYG, *Sc*-Glycolysis background) and IME576 (CEN.PK113-5D *tps1Δ* background) as measured by flow cytometry after incubation without C-source addition (red) or with galactose (blue) or glucose (orange) in duplicate experiments. The ratio between the fluorescence excitation at 510 and 515 nm

after excitation at 405 and 488 respectively is shown. Time of incubation (with or without C-source) is indicated. The control and SwYG (*Sc*-Glycolysis) strains behaved as expected, with an increase in the pH_i signal after sugar addition. The *tps1Δ* strain shows as strong decrease in the pHluorin signal upon glucose but not galactose addition, corresponding to previously published data. **E)** and **F)** Response of the pHluorin signal directly after addition of glucose or galactose in the IME481 (*Sc*-Glycolysis control) and IME576 (*tps1Δ*) strains. Again an immediate and sharp decrease in signal is seen for the *tps1Δ* strain upon glucose addition, while the addition of galactose leads to an increase in the signal.

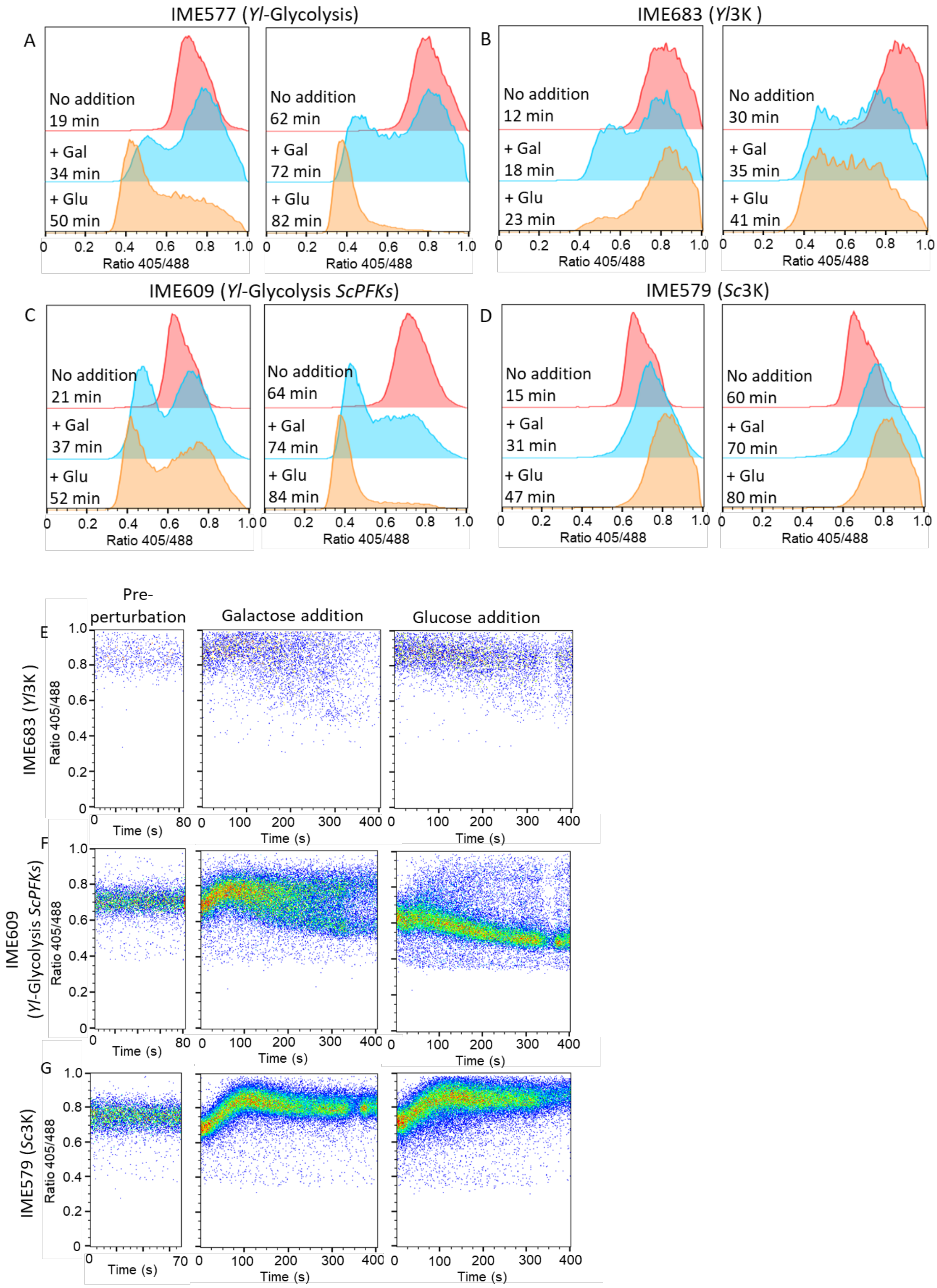

IME577 (Yl-Glycolysis)
IME683 (Yl3K)
A
B
No addition
19 min
+ Gal
34 min
+ Glu
50 min
No addition
62 min
+ Gal
72 min
+ Glu
82 min
No addition
12 min
+ Gal
18 min
+ Glu
23 min
No addition
30 min
+ Gal
35 min
+ Glu
41 min
Ratio 405/488
IME609 (Yl-Glycolysis ScPFKs)
IME579 (Sc3K)
C
D
No addition
21 min
+ Gal
37 min
+ Glu
52 min
No addition
64 min
+ Gal
74 min
+ Glu
84 min
No addition
15 min
+ Gal
31 min
+ Glu
47 min
No addition
60 min
+ Gal
70 min
+ Glu
80 min
Pre-perturbation
Galactose addition
Glucose addition
E
F
G
IME683 (Yl3K)
IME609 (Yl-Glycolysis ScPFKs)
IME579 (Sc3K)
Ratio 405/488
Time (s)

Figure S2 - pHluorin responses of the *Yl*-Glycolysis and mosaic glycolysis strains.

A)-D) pHluorin signal in the fluorescent population of strains IME577, IME683, IME609 and IME579 which express different combinations of *Yarrowia* glycolytic enzymes after incubation without C-source addition (red) or with galactose (blue) or glucose (orange) in duplicate experiments. Time of incubation (with or without C-source) is indicated. **E)-G)** Response of the pHluorin signal directly after addition of glucose or galactose in the IME683, IME609 and IME579 strains.

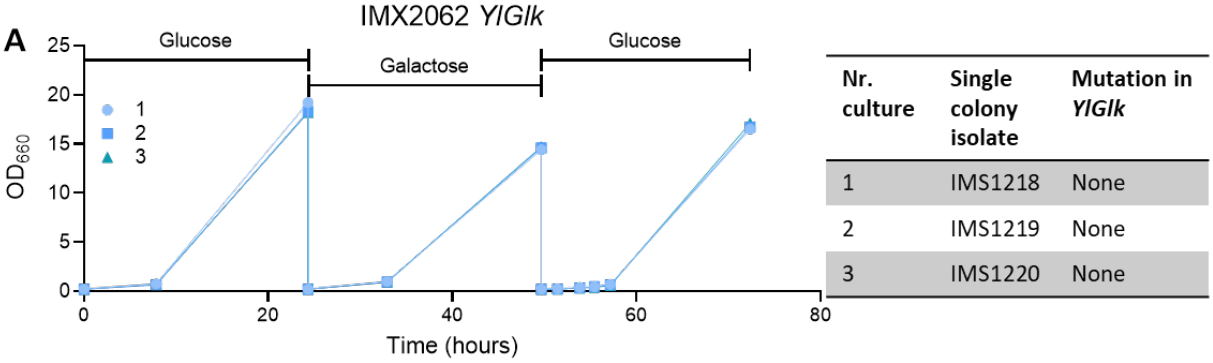

Nr. culture	Single colony isolate	Mutation in *YlGlk*
1	IMS1218	None
2	IMS1219	None
3	IMS1220	None

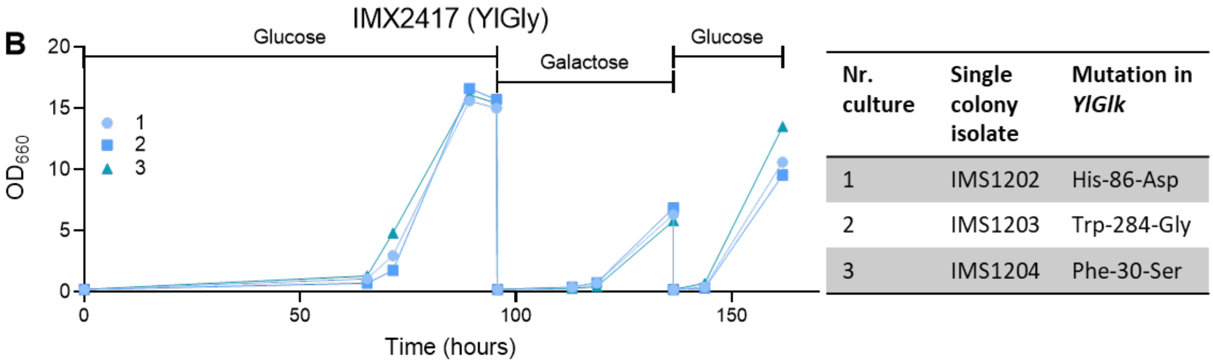

Nr. culture	Single colony isolate	Mutation in *YlGlk*
1	IMS1202	His-86-Asp
2	IMS1203	Trp-284-Gly
3	IMS1204	Phe-30-Ser

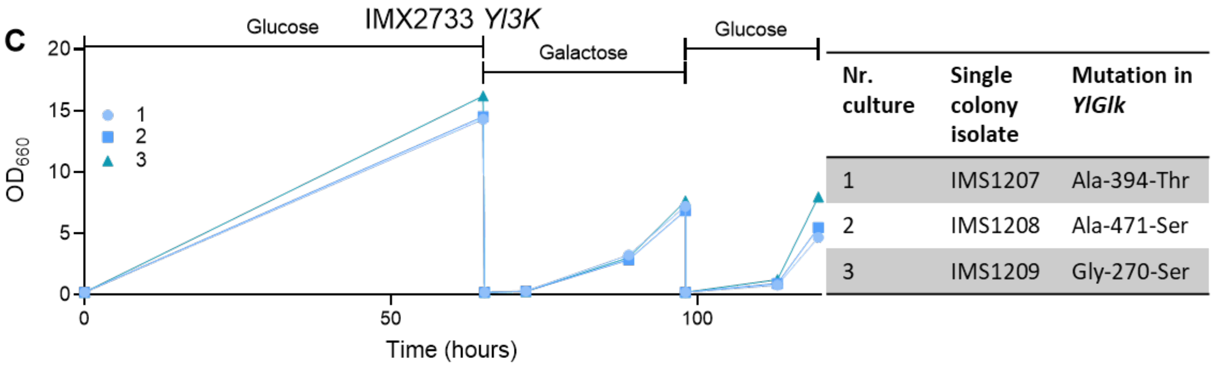

Nr. culture	Single colony isolate	Mutation in *YlGlk*
1	IMS1207	Ala-394-Thr
2	IMS1208	Ala-471-Ser
3	IMS1209	Gly-270-Ser

Figure S3 - Repeated transfer in glucose medium.
To determine whether adaptation to glucose medium was genetic or not strains were pre-grown on SM-Galactose liquid medium, then transferred to glucose in three independent cultures (indicated with 1,2,3,) at a starting OD_{660} of 0.2, after growth was observed these cultures were re-inoculated to non-selective SM-Galactose medium and after growth re-inoculated in glucose medium. After the final glucose cultures single colony isolates were checked for mutations. **A)** IMX2062 (*Yl*Glk complementation strain) showed a similar short lag phase when inoculated to a glucose culture for the second time, isolates IMS1218, IMS1219 and IMS1220 did not show mutations in the *YlGLK* gene. **B)** IMX2417 (*Yl*-Glycolysis strain) showed a lag phase on the first glucose culture of approximately 70 hours, consistent with that observed in Growth Profiler cultures. The second glucose culture appeared to start growth immediately and the *Y. lipolytica* glucokinase gene was mutated in all three resulting single colony isolates (see also Fig. 3C) **C)** IMX2733 (*Yl*-3K strain) similarly showed immediate growth in the second glucose culture and mutations the *YlGLK* gene were observed in each resulting single colony isolate.

A

	Percentage identity/similarity	
**Protein**	*Sc*Glk	*Kl*Glk
*Yl*Glk	39.9%/57.4%	40.7%/57.7%
*Sc*Glk		60.8%/74.5%

B

Strain	Mutated residue	Corresponding residue	
	*Yl*Glk	*Kl*Glk	*Sc*Glk
IMS1204 (YlGly)	F30	F31	F39
IMS1202 (YlGly)	H86	H93	H102
IMS1209 (Yl3K)	G270	G275	G291
IMS1203 (YlGly)	W284	Y289	Y305
IMS1207 (Yl3K)	A394	A392	A413
IMS1208 (Yl3K)	A471	A473	A494

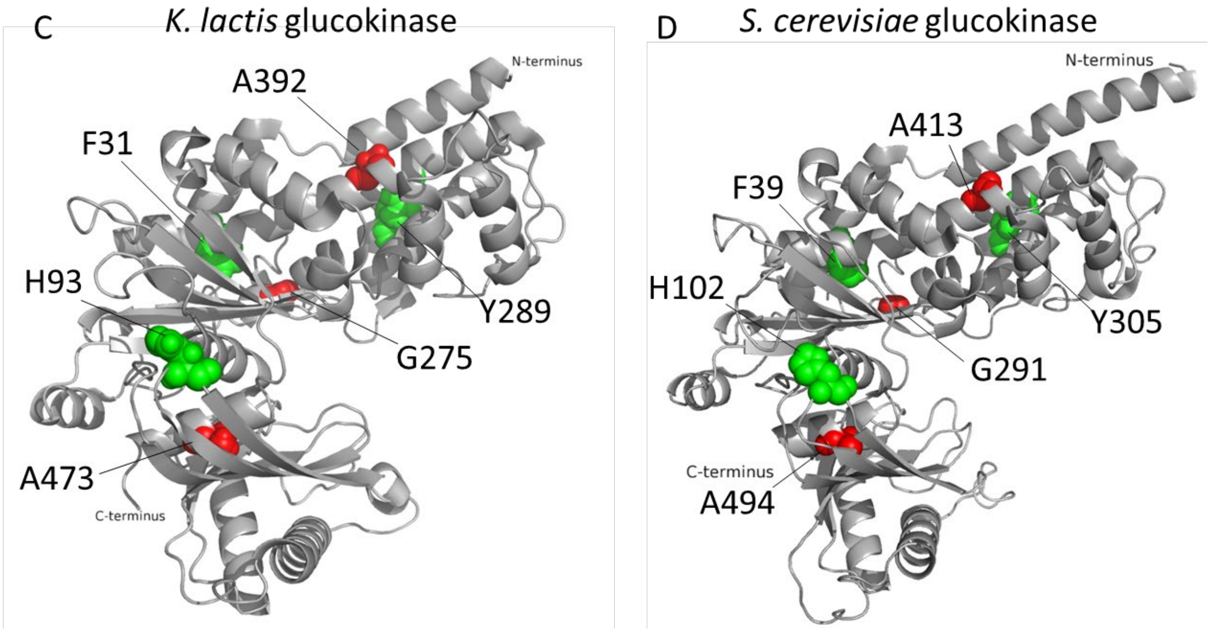

Figure S4 - Location of glucokinase mutations.
No crystal structure is currently available for the *Yarrowia lipolytica* glucokinase *(Yl*Glk*),* this enzyme does however share similarity to the *Kluyveromyces lactis* and *S. cerevisiae* glucokinases (*Kl*Glk and *Sc*Glk) for which crystal structures are available. **A)** Table showing percentages identity and similarity as determined by global pairwise alignment of the protein sequences (EMBOSS Needle). B) Comparison of the protein sequences shows the mutations found in this study occurred mostly in conserved amino acid residues which have a corresponding residue in each of the glucokinases. **C)** and **D)** Residues corresponding to those mutated in the *Yl*Glk shown in the *Kl*Glk and *Sc*Glk crystal structures. In green those found in the mutants of the *Yl*-glycolysis strain and in red those found in the mutants of the *Yl*-3K strain. Mutations are spread over the different domains and are not directly in the active site. PDB identifiers and source of structure *Kl*Glk: 6R2N from [1], *Sc*Glk: 6p4x [2].

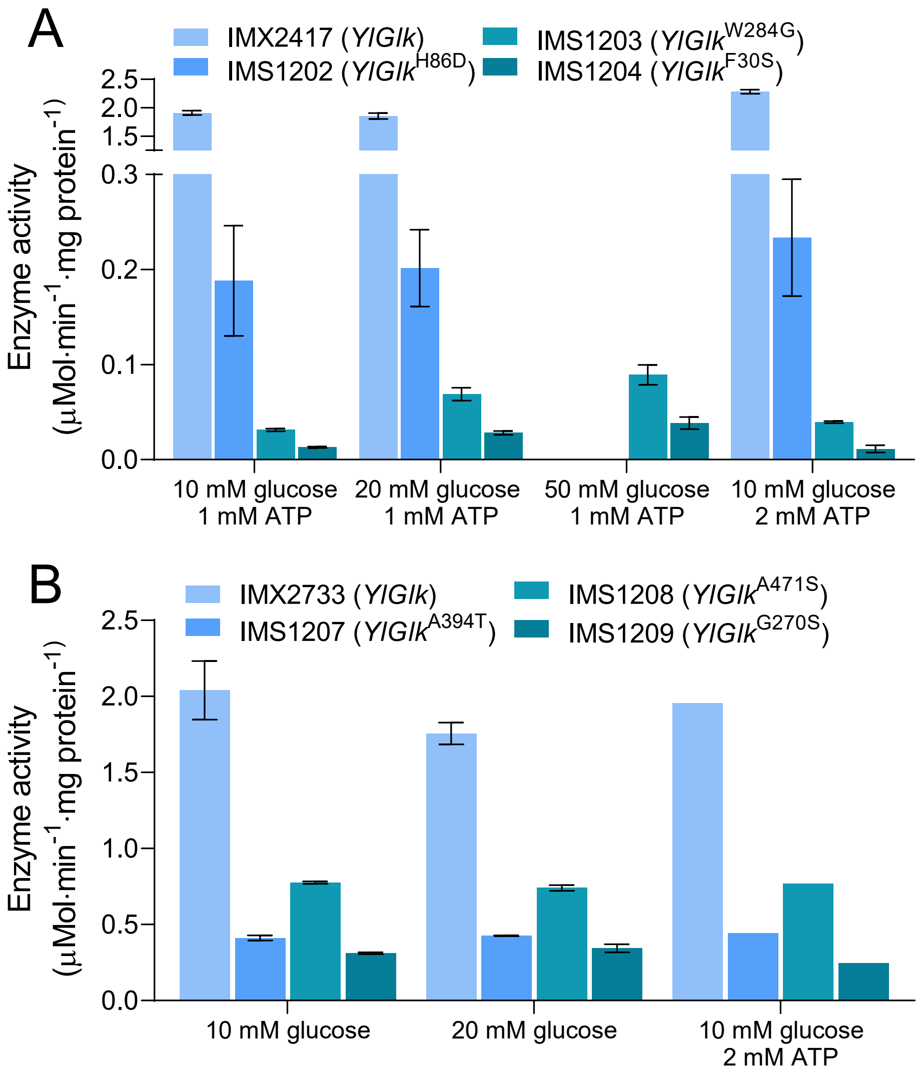

Figure S5 - Characterization of glucokinase mutants.

A) The glucokinase activity of the evolved isolates derived from *Yl*-Glycolysis strain IMX2417 (IMS1202-IMS1204) was determined at different glucose and ATP concentrations. The standard concentrations used were 10 mM glucose and 1 mM ATP. Increasing the glucose concentration to 20 and 50 mM increased activity of mutants *Yl*Glk^{W284G} and *Yl*Glk^{F30S}, but not the native enzyme or mutant *Yl*Glk^{H86D} suggesting an increased $K_{m,glucose}$ for those two mutants. **B)** Increasing the glucose or ATP concentration did not increase the activity of the *Yl*Glk mutants derived from strain IMX2733 (*Yl*-3K strain). Mean and SEM is shown for duplicate measurements except the increased ATP measurements of the IMX2733 strains which were measured in only one replicate.

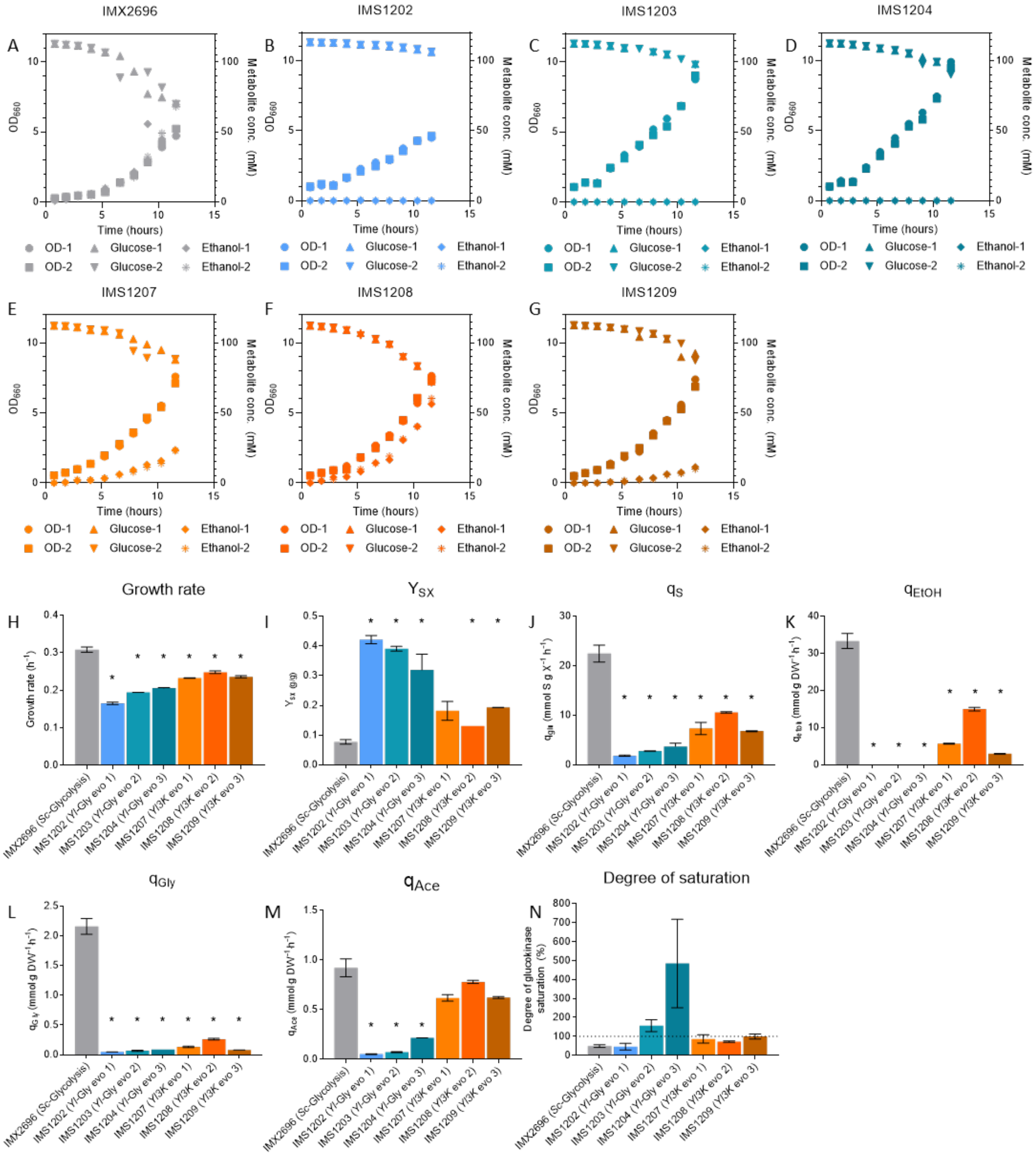

Figure S6 - Characterization of evolved isolates of the *Yl*-Glycolysis and *Yl*-3K strains.
The evolved isolates of the *Yl*-Glycolysis and *Yl*-3K strains and the control *Sc*-Glycolysis strain were grown on glucose minimal medium with urea to prevent acidification. **A)-G)** OD_{660} and metabolite profiles over time of duplicate cultures of each strain. **H)-M)** Estimations of growth rate, biomass yield, glucose uptake rate and ethanol, glycerol and acetate production rates based on the measured metabolite profiles, mean and SEM are shown significant differences to control strain IMX2696 indicated by * (T.Test, homoscedastic, unpaired P<0.05). **N)** Estimated degree of saturation of the hexokinase/glucokinase reaction based on the highest measured activities (Supplementary Fig. S5) and the glucose uptake rate (q_{glu}) estimated for each strain. Error bars indicate summed relative standard deviation for both measurements.

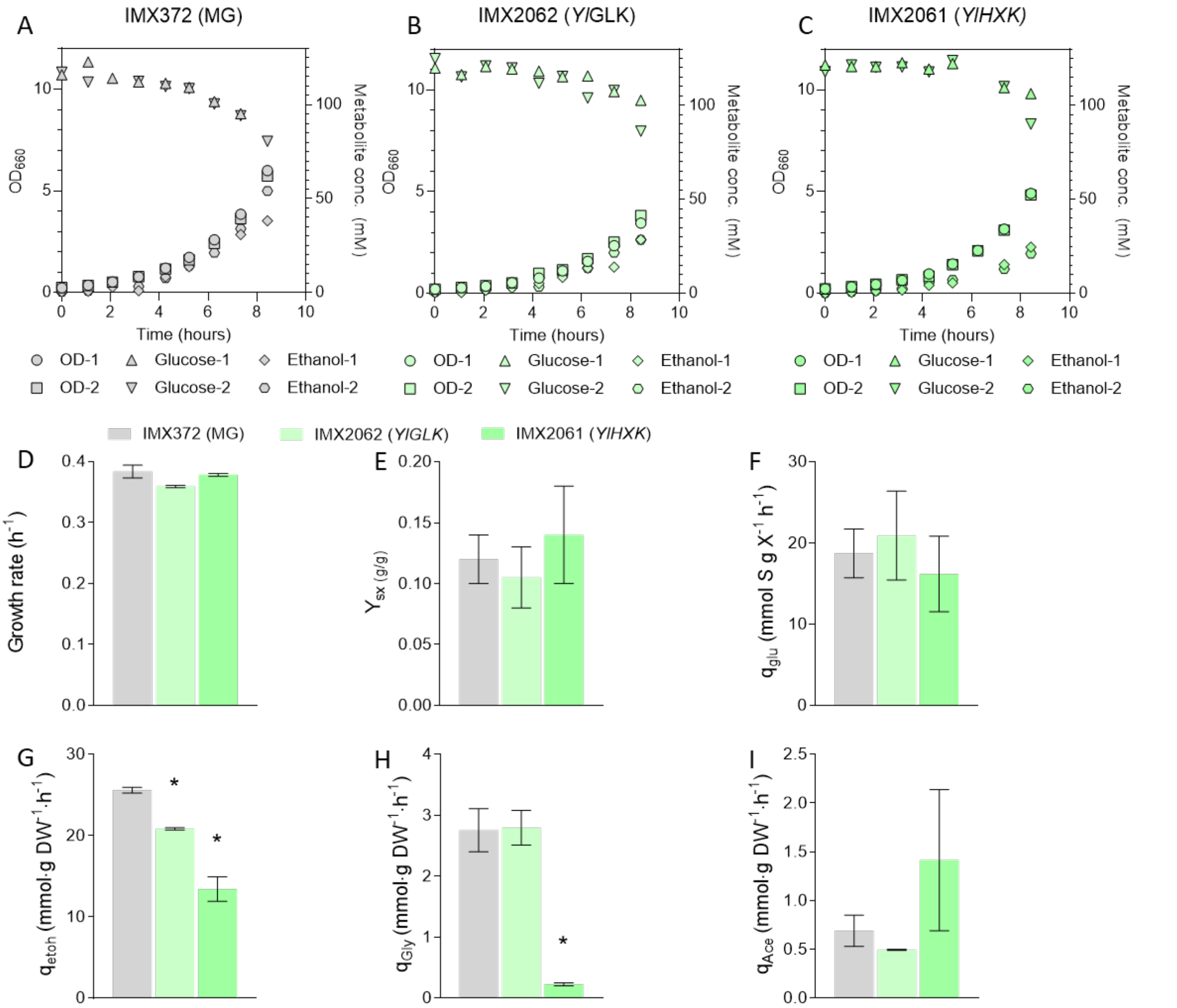

Figure S7 - Characterization of glucokinase and hexokinase complementation strains.
The complementation strains expressing the *YlGLK* and *YlGLK* genes and the control Minimal Glycolysis strain were grown on glucose minimal medium to measure growth rate, glucose uptake and ethanol production. **A)-C)** OD_{660} and metabolite profiles over time of duplicate cultures of each strain. **D)-I)** Estimations of growth rate, biomass yield, glucose uptake rate and ethanol, glycerol and acetate production rates based on the measured metabolite profiles, mean and SEM are shown, significant differences to control strain IMX372 indicated by * (T.Test, homoscedastic, unpaired P<0.05).

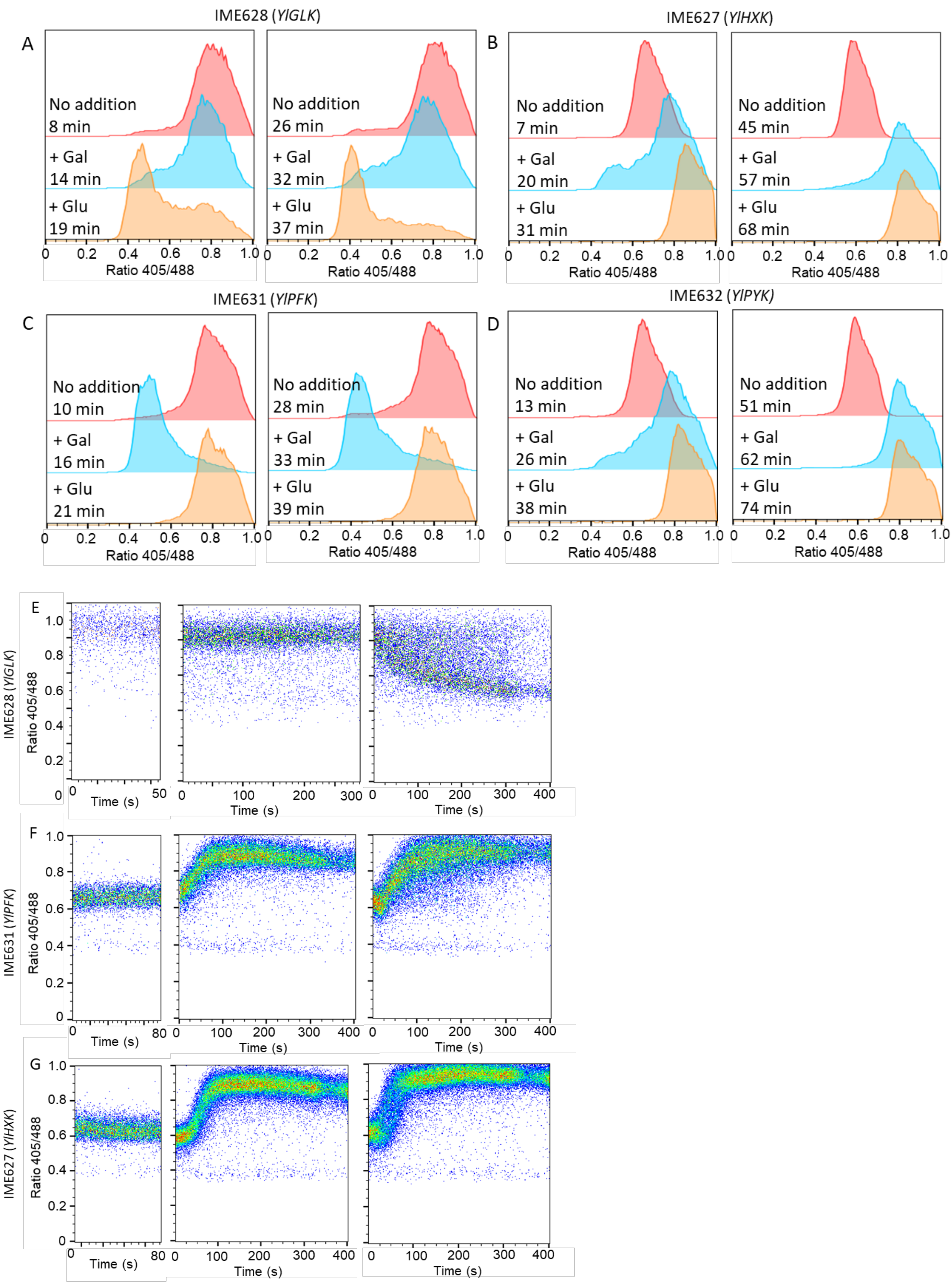

Figure S8 - pHluorin response of single complementation strains.

A)-D) pHluorin signal in the fluorescent population of strains IME628, IME627, IME631 and IME632 which express different single *Yarrowia* glycolytic enzymes after incubation without C-source addition

(red) or with galactose (blue) or glucose (orange) in duplicate experiments. Time of incubation (with or without C-source) is indicated. **E)-G)** Response of the pHluorin signal directly after addition of glucose or galactose in the IME628, IME631 and IME627 strains.

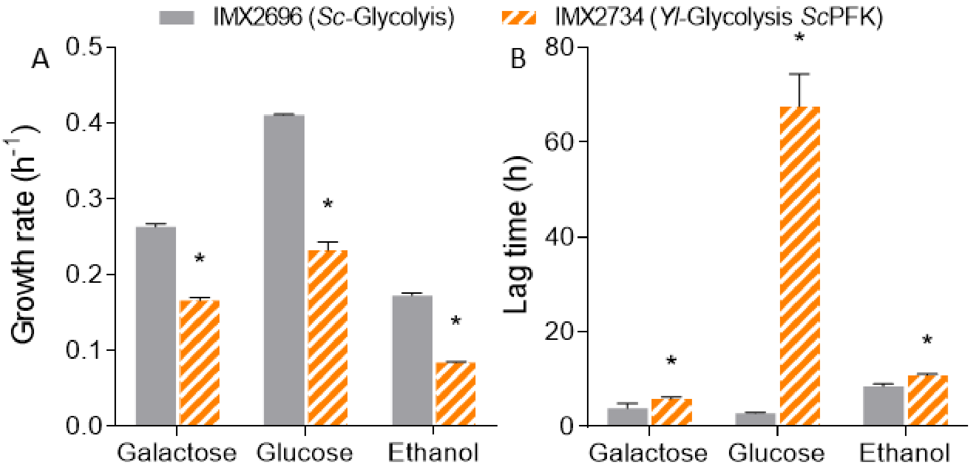

Figure S9 - Growth rate and lag phase of *Yl*-Glycolysis strain with *Sc*Pfk.
A) Growth rates measured in the growth profiler on galactose, glucose and ethanol for strain IMX2734, expressing the *Yarrowia lipolytica* glycolysis except phosphofructokinase, for which it has the *ScPFK* genes and control strain IMX2696 (*Sc*-Glycolysis, IMX2696). **B)** Growth on glucose was only observed after a lag phase of up to 75 hours similar to the *Yl*-Glycolysis strain. Mean and SEM of triplicates are shown, * indicates significant difference (T.Test, homoscedastic, unpaired, P<0.05).

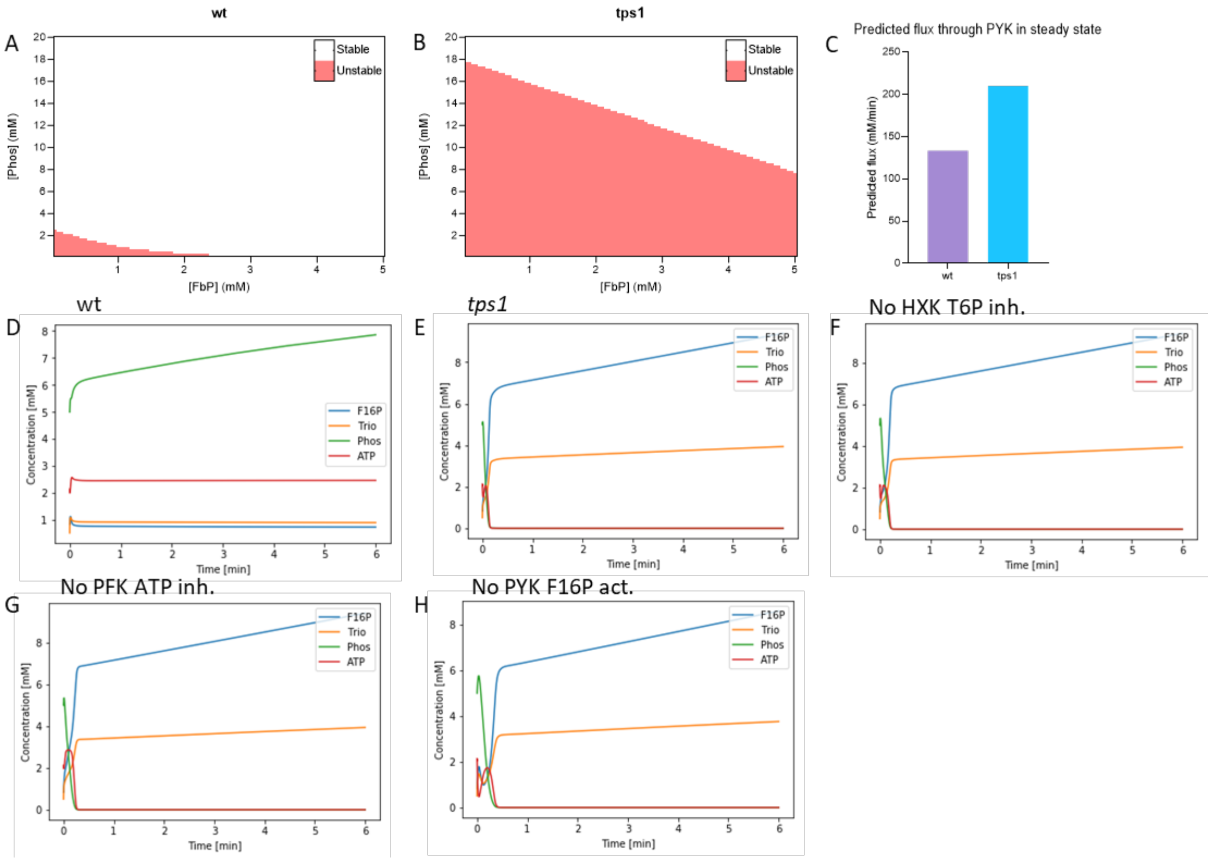

Figure S10 - Predicted metabolite time courses.

The model results were reproduced for a 'wt' and a *tps1* deletion mutant. **A)** and **B)** Division between balanced and imbalanced states in the wildtype and *tps1* models. With red showing the imbalanced state and white steady state. **C)** Predicted flux through the pyruvate kinase in the balanced state for both model types. **D)-H)** Predicted metabolite time courses for the first six minutes with various model configurations. Imbalanced starting concentrations of FBP and Phosphate were chosen (FBP_i: 0.836 mM, $Phos_i$: 5.0 mM). Time courses show similar behaviour for all imbalanced systems, with accumulation of FBP and depletion of phosphate. For the wildtype a steady state is reached with these initial conditions after ~50 minutes.

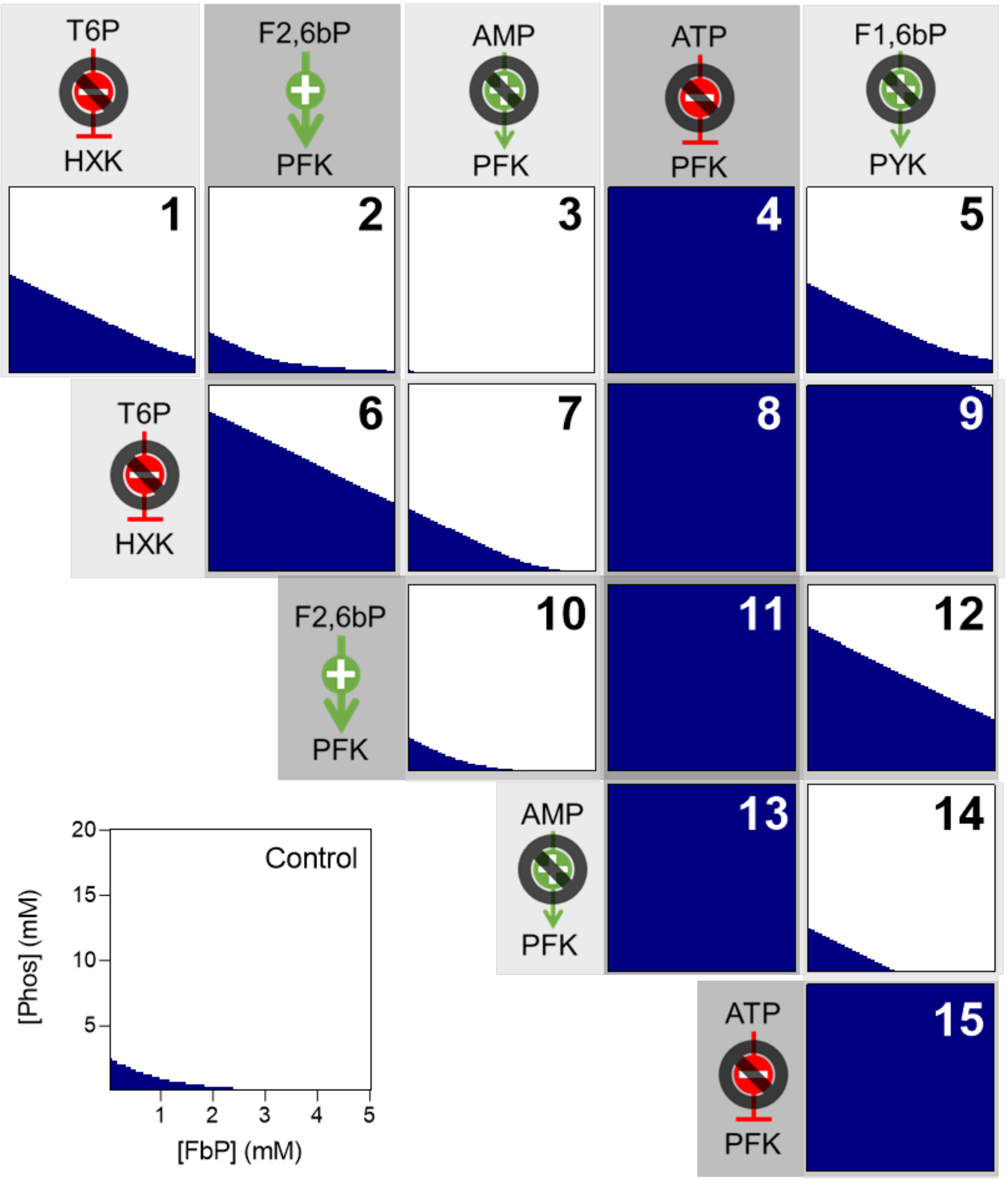

Figure S11 - Combinatorial effects of removing regulation in a mathematical model of glycolysis. The outcome of the glycolytic model is shown as a function of the initial concentrations of F1,6bP and phosphate, with dark blue indicating an imbalanced outcome and white a balanced steady state. In the bottom left the situation in the unmodified control model is shown, the same initial concentrations were tested for all model configurations. In plots 1-5 the effect on the model outcome of removal of single allosteric regulations is shown similar to Figure 6A. In the plots below combinatorial removal of two regulations is shown, with one removed in each row. Overall combinatorial removal was detrimental to stability, with the exception of removing AMP activation of PFK, which is also stabilizing on its own.

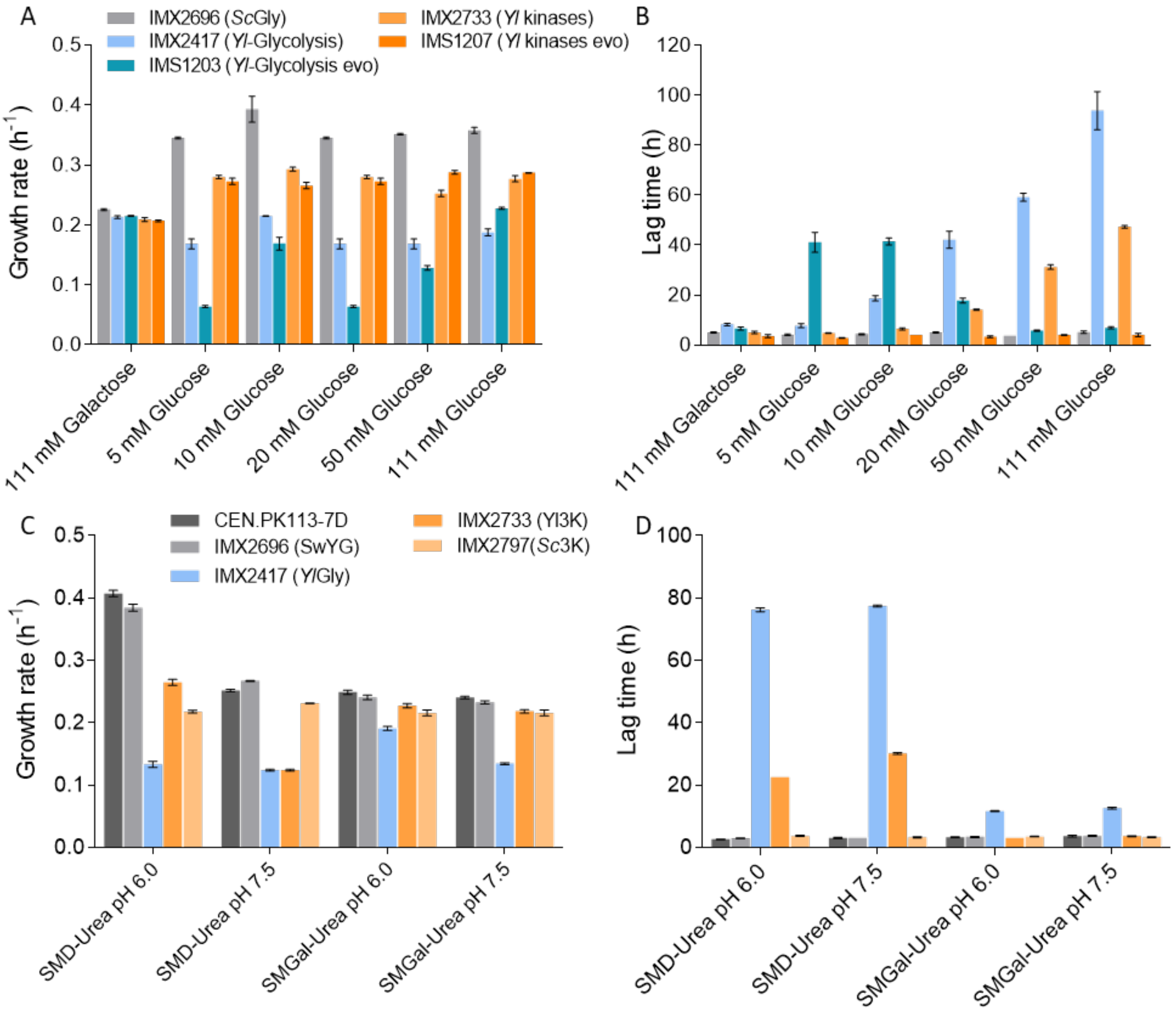

Figure S12 - Characterization of strains on different glucose concentrations and at high pH.
A) and **B)** Growth rates and lag-times determined by growth in the growth profiler of the *Yl*-glycolysis and *Yl*-3K strains and the control *Sc*-Glycolysis strain on galactose and at various glucose concentrations. **C)** and **D)** Growth rates of the *Yl*-Glycolysis and *Yl*-3K and *Sc*-3K strains in normal pH (6.0) and high pH (7.5) media to check the presence of a growth defect from dysfunction of the moonlighting function of yeast aldolase. Growth rates and lag-times were largely unaffected by the increased pH for the strains expressing *Y. lipolytica* glycolytic genes.

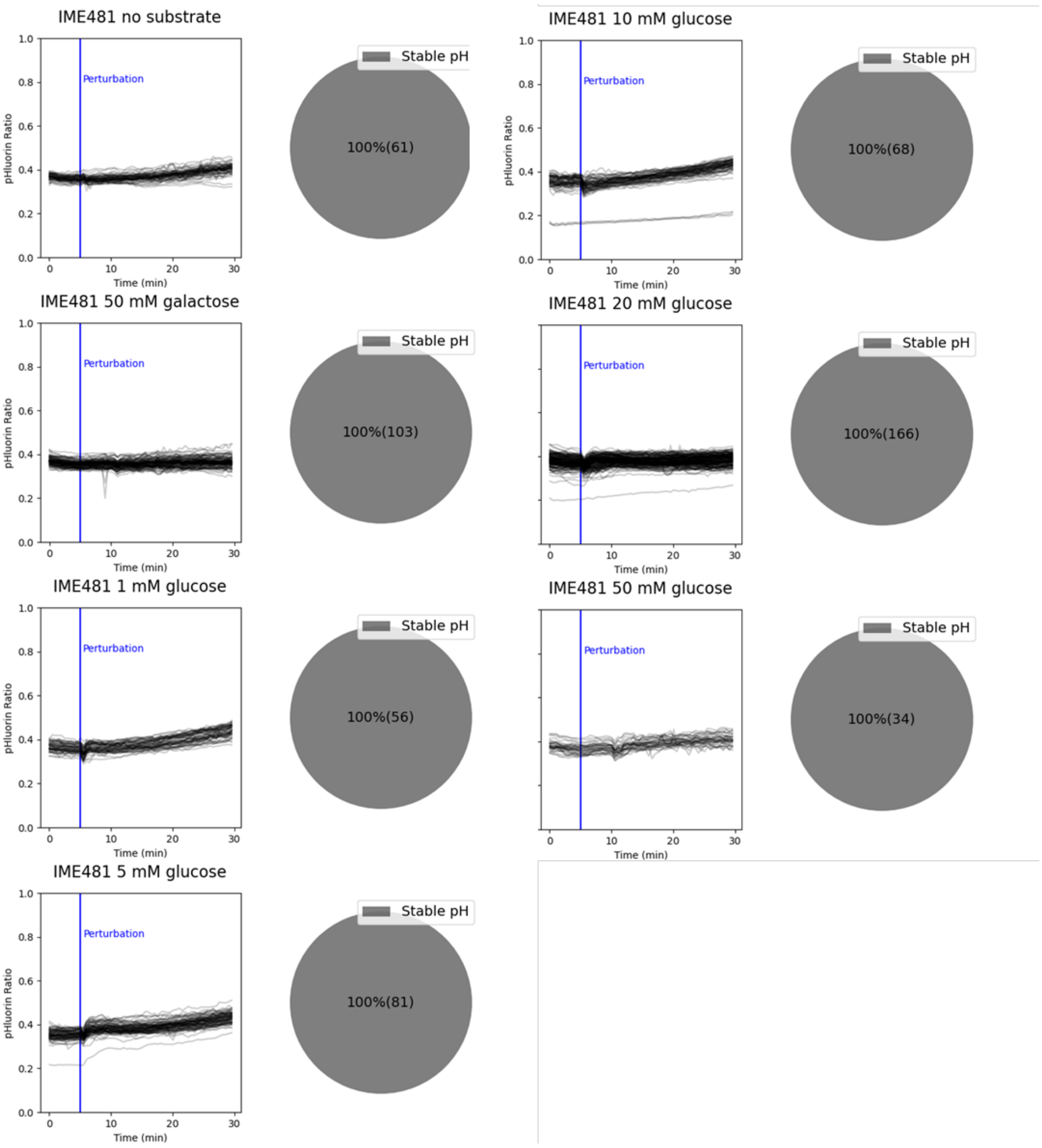

Figure S13 - Characterization of pHi heterogeneity upon sugar addition to strain *Sc*-Gly (IME481). The timecourse for each condition is shown with a line showing the trace of pH_i per tracked cell. The percentage stable cells and number of total cells is sown in the pie charts.

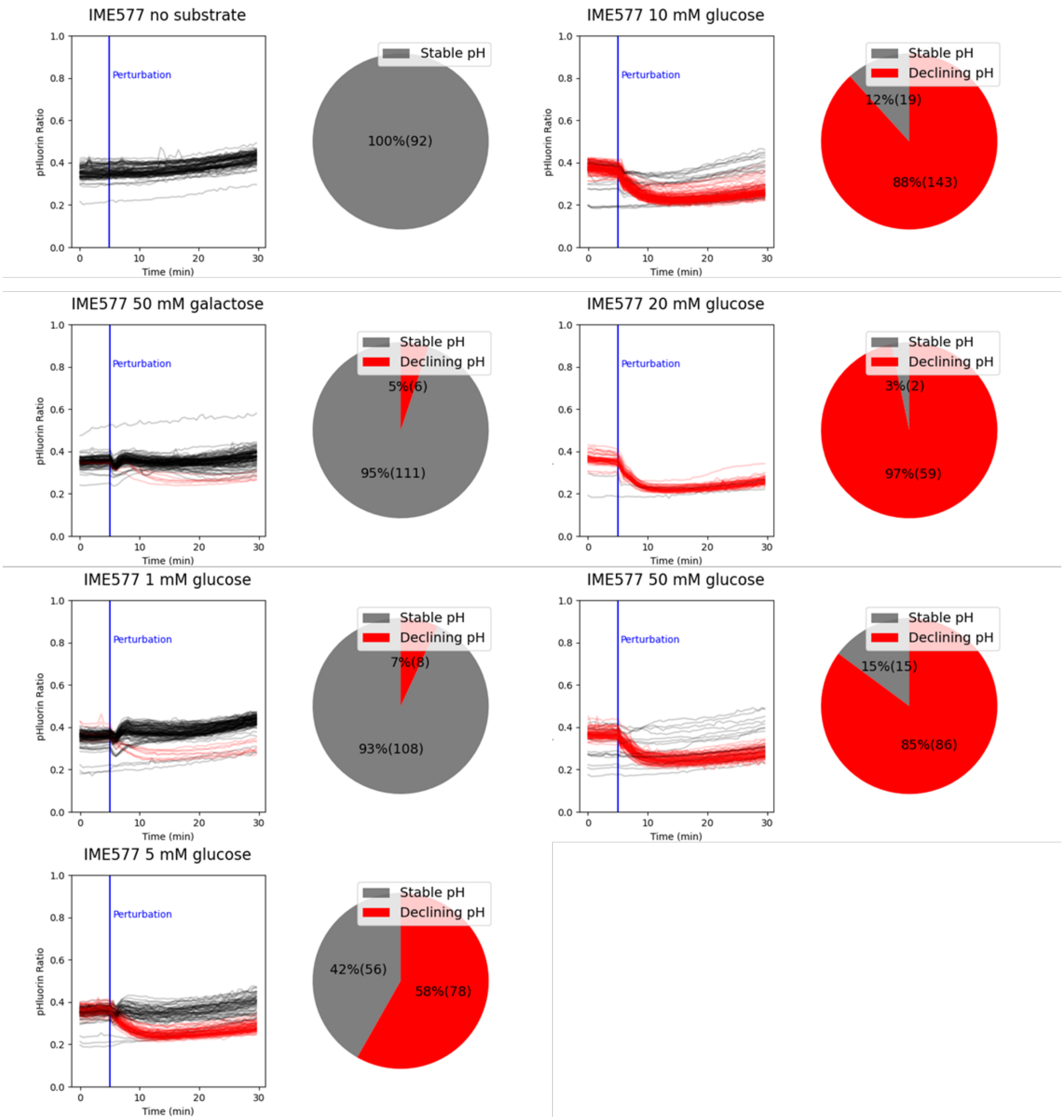

Figure S14 - Characterization of pHi heterogeneity upon sugar addition to strain *Yl*-Gly (IME577). The timecourse for each condition is shown with a line showing the trace of pH_i per tracked cell. The percentage stable cells and number of total cells is sown in the pie charts.

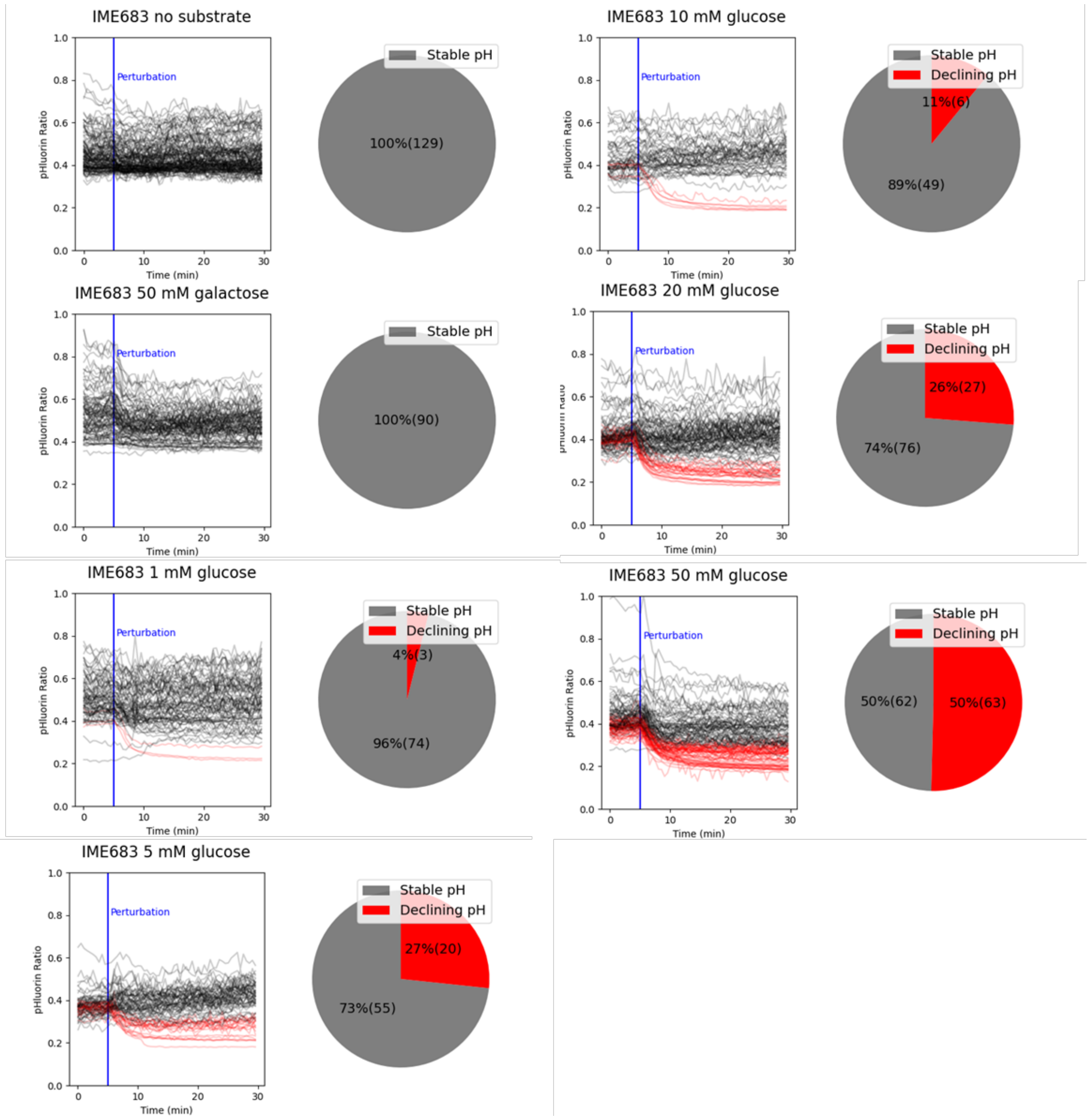

Figure S15 - Characterization of pHi heterogeneity upon sugar addition to strain *Yl*-3K (IME683). The timecourse for each condition is shown with a line showing the trace of pH_i per tracked cell. The percentage stable cells and number of total cells is sown in the pie charts.

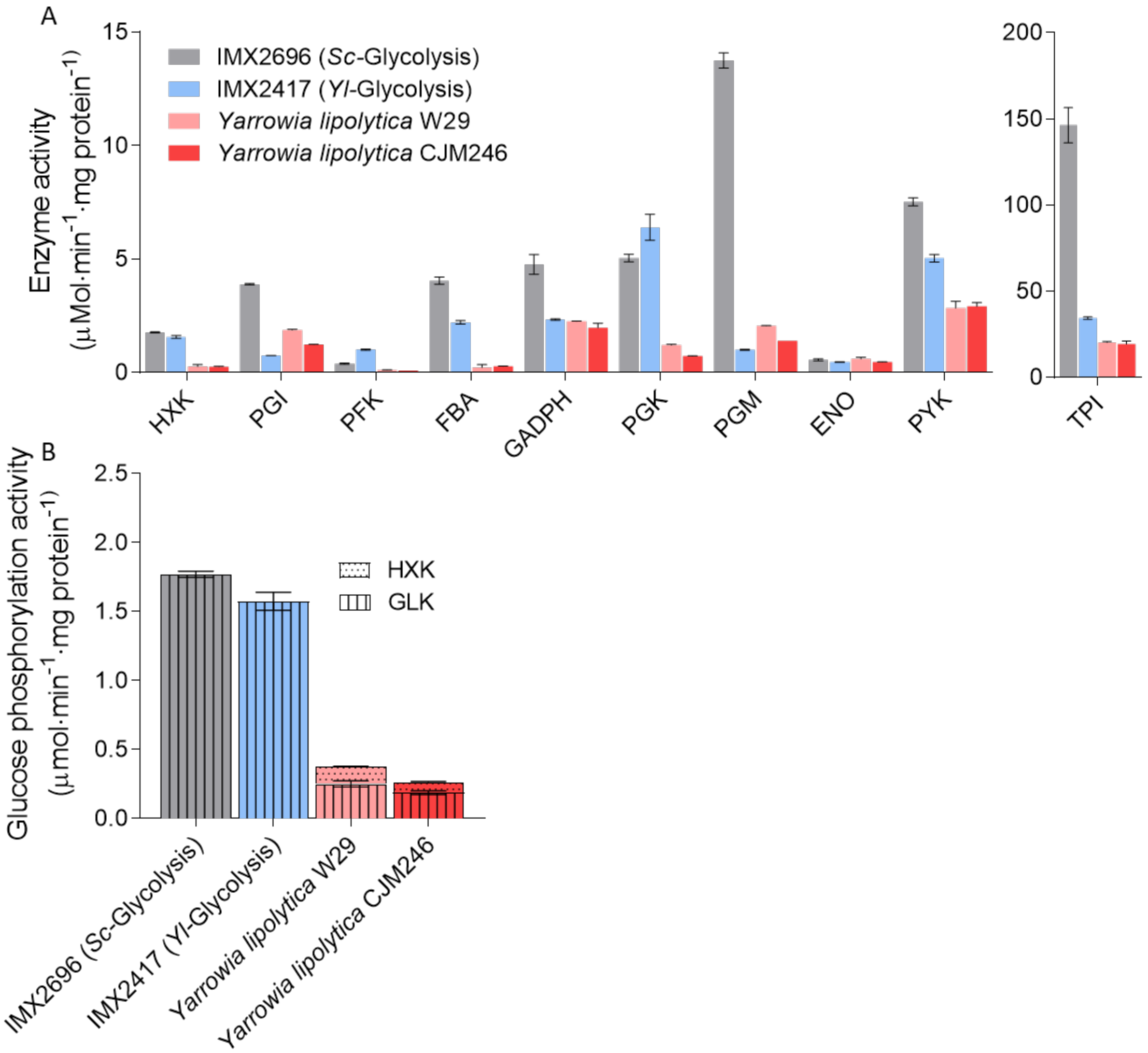

Figure S16 - Glycolytic activities in *Yarrowia lipolytica.*

Activity of glycolytic enzymes in *Yarrowia lipolytica* extracts. **A)** *In vitro* measured activities for two *Yarrowia lipolytica* strains, W29 a wildtype strain and laboratory strain CJM246 (PO1a). Activities of the *S. cerevisiae* strains IMX2696 and IMX2417 expressing the *Yarrowia* enzymes are shown for comparison. All glycolytic enzyme activities are significantly lower in *Y. lipolytica.* **B)** Separation of the glucokinase and hexokinase activities, based on measurements of the glucose and fructose phosphorylation activity, assuming a fructose/glucose phosphorylation ratio of 1.4 for hexokinase and an absence of glucokinase activity on fructose [3]. Glucokinase was the major isoenzyme in *Y. lipolytica* as expected, accounting for 66 and 71% of glucose phosphorylation activity in the W29 and CJM246 respectively.

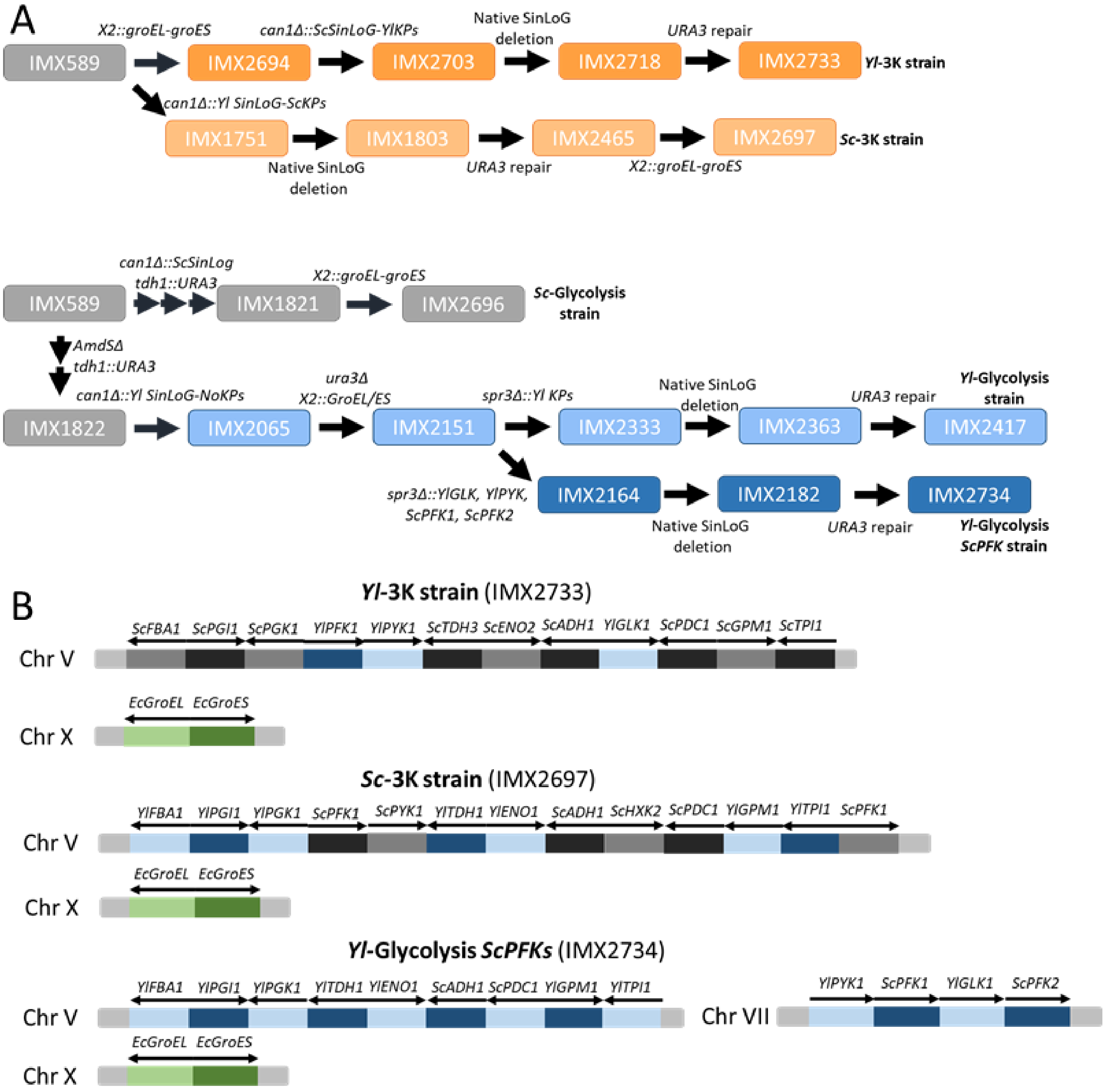

Figure S17 - Overview of strain construction.
A) Overview of strain construction leading to the most important single locus glycolysis strains. Each step signifies a round of transformation and selection and a genetic modification. The construction of IMX589, IMX1821 and IMX1822 is described elsewhere [4, 5]. **B)** Overview of the main genetic loci in the key strains, *S. cerevisiae* genes are indicated in black and grey, *Yarrowia lipolytica* genes in blue, bacterial genes in green.

Table S1 - Strains used in this study

Table S1A - Control and minimal glycolysis strains

Strain	Description	Genotype	Reference
CEN.PK113-7D	Prototrophic reference	*MATa URA3 TRP1 LEU2 HIS3 MAL2-8c SUC2*	[6]
CEN.PK113-5D	Uracil auxotrophic reference	*MATa ura3-52 TRP1 LEU2 HIS3 MAL2-8c SUC2*	[6]
W29	*Y. lipolytica* wildtype	*MATA*	
CJM246 (also known as PO1a)	*Y. lipolytica* cDNA donor strain	*MATA leu2-270 ura3-302*	Obtained from C.L.-Flores
IMX581	Cas9 expressing uracil auxotrophic CEN.PK strain	*MATa ura3-52 TRP1 LEU2 HIS3 MAL2-8c SUC2 can1Δ::cas9-natNT2*	[7]
IMX2243	*tps1* deletion strain uracil auxotrophic	*MATa ura3-52 TRP1 LEU2 HIS3 MAL2-8c SUC2 can1Δ::cas9-natNT2 tps1Δ*	This study
IMX372	Minimal glycolysis (MG) strain prototrophic	*MATa ura3-52 his3-1 leu2-3,112 MAL2-8c SUC2 glk1Δ::(pAgTEF1-SpHIS5-tAgTEF1) hxk1Δ::KlLEU2 tdh1Δ::URA3 tdh2Δ gpm2Δ gpm3Δ eno1Δ pyk2Δ pdc5Δ pdc6Δ adh2Δ adh5Δ adh4Δ*	[8]
IMX1076	Minimal glycolysis (MG) strain Auxotrophic	*MATa ura3-52 his3-1 leu2-3,112 MAL2-8c SUC2 glk1Δ::(pAgTEF1-SpHIS5-tAgTEF1) hxk1Δ::KlLEU2 tdh1Δ tdh2Δ gpm2Δ gpm3Δ eno1Δ pyk2Δ pdc5Δ pdc6Δ adh2Δ adh5Δ adh4Δ, sga1::(cas9 natNT1)*	[5]

Table S1B - *Sc*-Glycolysis strains

IMX589	SwYG strain, *sga1* auxotrophic	*MATa ura3-52 his3-1 leu2-3,112 MAL2-8c SUC2 glk1Δ::(pAgTEF1-SpHIS5-tAgTEF1) hxk1Δ::KlLEU2 tdh1Δ tdh2Δ gpm2Δ gpm3Δ eno1Δ pyk2Δ pdc5Δ pdc6Δ adh2Δ adh5Δ adh4Δ sga1Δ::(FBA1* $_{H}$ *TPI1* $_{P}$ *PGK1* $_{Q}$ *ADH1* $_{N}$ *PYK1* $_{O}$ *TDH3* $_{A}$ *ENO2* $_{B}$ *HXK2* $_{C}$ *PGI1* $_{D}$ *PFK1* $_{J}$ *PFK2* $_{K}$ *AmdSYM* $_{L}$ *GPM1* $_{M}$ *PDC1 SYN* $_{F}$*) pyk1Δ pgi1Δ tpi1Δ tdh3Δ pfk2Δ::(Spcas9 natNT1) pgk1Δ gpm1Δ fba1Δ hxk2Δ pfk1Δ adh1Δ pdc1Δ eno2Δ*	[4]
IMX1821	SwYG strain, *can1* prototrophic	*MATa ura3-52 his3-1 leu2-3,112 MAL2-8c SUC2 glk1Δ::(pAgTEF1-SpHIS5-tAgTEF1) hxk1Δ::KlLEU2 tdh1Δ::URA3 tdh2Δ gpm2Δ gpm3Δ eno1Δ pyk2Δ pdc5Δ pdc6Δ adh2Δ adh5Δ adh4Δ sga1Δ pyk1Δ pgi1Δ tpi1Δ tdh3Δ pfk2Δ::(pTEF1-Spcas9-tCYC1 natNT1) pgk1Δ gpm1Δ fba1Δ hxk2Δ pfk1Δ adh1Δ pdc1Δ eno2Δ can1::(FBA1* $_{H}$ *TPI1* $_{P}$ *PGK1* $_{Q}$ *ADH1* $_{N}$ *PYK1* $_{O}$ *TDH3* $_{A}$ *ENO2* $_{B}$ *HXK2* $_{C}$ *PGI1* $_{D}$ *PFK1* $_{J}$ *PFK2* $_{K}$ *KanMX* $_{L}$ *GPM1* $_{M}$ *PDC1* $_{F}$*)*	[5]
IMX1822	SwYG strain, *sga1* prototrophic	*MATa ura3-52 his3-1 leu2-3,112 MAL2-8c SUC2 glk1Δ::(pAgTEF1-SpHIS5-tAgTEF1) hxk1Δ::KlLEU2 tdh1Δ::(pURA3-URA3-tURA3) tdh2Δ gpm2Δ gpm3Δ eno1Δ pyk2Δ pdc5Δ pdc6Δ adh2Δ adh5Δ adh4Δ sga1Δ::(FBA1* $_{H}$ *TPI1* $_{P}$ *PGK1* $_{Q}$ *ADH1* $_{N}$ *PYK1* $_{O}$ *TDH3* $_{A}$ *ENO2* $_{B}$ *HXK2* $_{C}$ *PGI1* $_{D}$ *PFK1* $_{J}$ *PFK2* $_{K}$ $_{L}$ *GPM1* $_{M}$ *PDC1* $_{F}$ *) pyk1Δ pgi1Δ tpi1Δ tdh3Δ pfk2Δ::(pTEF1-Spcas9-tCYC1 natNT1) pgk1Δ gpm1Δ fba1Δ hxk2Δ pfk1Δ adh1Δ pdc1Δ eno2Δ*	[5]
IMX2694	SwYG strain, *sga1* auxotrophic,	*MATa ura3-52 his3-1 leu2-3,112 MAL2-8c SUC2 glk1Δ::(pAgTEF1-SpHIS5-tAgTEF1) hxk1Δ::KlLEU2 tdh1Δ tdh2Δ gpm2Δ gpm3Δ eno1Δ pyk2Δ pdc5Δ pdc6Δ adh2Δ adh5Δ*	This study

	GroEL/ES integrated	*adh4Δ sga1Δ::(FBA1 $_{H}$ TPI1 $_{P}$ PGK1 $_{Q}$ ADH1 $_{N}$ PYK1 $_{O}$ TDH3 $_{A}$ ENO2 $_{B}$ HXK2 $_{C}$ PGI1 $_{D}$ PFK1 $_{J}$ PFK2 $_{K}$ AmdSYM $_{L}$ GPM1 $_{M}$ PDC1 SYN $_{F}$) pyk1Δ pgi1Δ tpi1Δ tdh3Δ pfk2Δ::(Spcas9 natNT1) pgk1Δ gpm1Δ fba1Δ hxk2Δ pfk1Δ adh1Δ pdc1Δ eno2Δ X2::GroEL$_{AA}$GroES*	
IMX2696	*Sc*-Glycolysis control SwYG strain, GroEL/ES integrated	*MATa ura3-52 his3-1 leu2-3,112 MAL2-8c SUC2 glk1Δ::(pAgTEF1-SpHIS5-tAgTEF1) hxk1Δ::KlLEU2 tdh1Δ::URA3 tdh2Δ gpm2Δ gpm3Δ eno1Δ pyk2Δ pdc5Δ pdc6Δ adh2Δ adh5Δ adh4Δ sga1Δ pyk1Δ pgi1Δ tpi1Δ tdh3Δ pfk2Δ::(pTEF1-Spcas9-tCYC1 natNT1) pgk1Δ gpm1Δ fba1Δ hxk2Δ pfk1Δ adh1Δ pdc1Δ eno2Δ can1::(FBA1 $_{H}$ TPI1 $_{P}$ PGK1 $_{Q}$ ADH1 $_{N}$ PYK1 $_{O}$ TDH3 $_{A}$ ENO2 $_{B}$ HXK2 $_{C}$ PGI1 $_{D}$ PFK1 $_{J}$ PFK2 $_{K}$ KanMX $_{L}$ GPM1 $_{M}$ PDC1 $_{F}$) X2::GroEL$_{AA}$GroES*	This study

Table S1C - *Yl*-Glycolysis strains

Strain	Description of modification	Genotype	Reference
IMX2065	*Yl* glycolysis genes integrated in *can1*	*MATa ura3-52 his3-1 leu2-3,112 MAL2-8c SUC2 glk1Δ::(pAgTEF1-SpHIS5-tAgTEF1) hxk1Δ::KlLEU2 tdh1Δ::(pURA3-URA3-tURA3) tdh2Δ gpm2Δ gpm3Δ eno1Δ pyk2Δ pdc5Δ pdc6Δ adh2Δ adh5Δ adh4Δ sga1Δ::($_{G}$ tFBA1-FBA1-pFBA1 $_{H}$ pTPI1-TPI1-tTPI1 $_{P}$ tPGK1-PGK1-pPGK1 $_{Q}$ tADH1-ADH1-pADH1 $_{N}$ pPYK1-PYK1-tPYK1 $_{O}$ tTDH3-TDH3-pTDH3 $_{A}$ pENO2-ENO2-tENO2 $_{B}$ pHXK2-HXK2-tHXK2 $_{C}$ pPGI-PGI1-tPGI1 $_{D}$ pPFK1-PFK1-tPFK1 $_{J}$ tPFK2-PFK2-pPFK2 $_{K}$ $_{L}$ tGPM1-GPM1-pGPM1 $_{M}$ pPDC1-PDC1-tPDC1-SYN $_{F}$) pyk1Δ pgi1Δ tpi1Δ tdh3Δ pfk2Δ::(pTEF1-Spcas9-tCYC1 natNT1) pgk1Δ gpm1Δ fba1Δ hxk2Δ pfk1Δ adh1Δ pdc1Δ eno2Δ can1::(YlFBA$_{BA}$YlPGI$_{BI}$YlPGK$_{BJ}$YlTDH1$_{BE}$YlENO$_{BF}$ScADH1$_{BC}$ ScPDC1$_{BD}$YlGPM$_{BK}$YlTPI)*	This study
IMX2151	*Yl* glycolysis genes integrated in *can1*, GroEL/ES integrated, *URA3* deleted	*MATa ura3-52 his3-1 leu2-3,112 MAL2-8c SUC2 glk1Δ::(pAgTEF1-SpHIS5-tAgTEF1) hxk1Δ::KlLEU2 tdh1Δ tdh2Δ gpm2Δ gpm3Δ eno1Δ pyk2Δ pdc5Δ pdc6Δ adh2Δ adh5Δ adh4Δ sga1Δ::($_{G}$ tFBA1-FBA1-pFBA1 $_{H}$ pTPI1-TPI1-tTPI1 $_{P}$ tPGK1-PGK1-pPGK1 $_{Q}$ tADH1-ADH1-pADH1 $_{N}$ pPYK1-PYK1-tPYK1 $_{O}$ tTDH3-TDH3-pTDH3 $_{A}$ pENO2-ENO2-tENO2 $_{B}$ pHXK2-HXK2-tHXK2 $_{C}$ pPGI-PGI1-tPGI1 $_{D}$ pPFK1-PFK1-tPFK1 $_{J}$ tPFK2-PFK2-pPFK2 $_{K}$ $_{L}$ tGPM1-GPM1-pGPM1 $_{M}$ pPDC1-PDC1-tPDC1-SYN $_{F}$) pyk1Δ pgi1Δ tpi1Δ tdh3Δ pfk2Δ::(pTEF1-Spcas9-tCYC1 natNT1) pgk1Δ gpm1Δ fba1Δ hxk2Δ pfk1Δ adh1Δ pdc1Δ eno2Δ can1::(YlFBA$_{BA}$YlPGI$_{BI}$YlPGK$_{BJ}$YlTDH1$_{BE}$YlENO$_{BF}$ScADH1$_{BC}$ ScPDC1$_{BD}$YlGPM$_{BK}$YlTPI) X2::GroEL$_{AA}$GroES*	This study
IMX2333	*Yl* kinases integrated in *SPR3*	*MATa ura3-52 his3-1 leu2-3,112 MAL2-8c SUC2 glk1Δ::(pAgTEF1-SpHIS5-tAgTEF1) hxk1Δ::KlLEU2 tdh1Δ tdh2Δ gpm2Δ gpm3Δ eno1Δ pyk2Δ pdc5Δ pdc6Δ adh2Δ adh5Δ adh4Δ sga1Δ::($_{G}$ tFBA1-FBA1-pFBA1 $_{H}$ pTPI1-TPI1-tTPI1 $_{P}$ tPGK1-PGK1-pPGK1 $_{Q}$ tADH1-ADH1-pADH1 $_{N}$ pPYK1-PYK1-tPYK1 $_{O}$ tTDH3-TDH3-pTDH3 $_{A}$ pENO2-ENO2-tENO2 $_{B}$ pHXK2-HXK2-tHXK2 $_{C}$ pPGI-PGI1-tPGI1 $_{D}$ pPFK1-PFK1-tPFK1 $_{J}$ tPFK2-PFK2-pPFK2 $_{K}$ $_{L}$ tGPM1-GPM1-pGPM1 $_{M}$ pPDC1-PDC1-tPDC1-SYN $_{F}$) pyk1Δ pgi1Δ tpi1Δ tdh3Δ pfk2Δ::(pTEF1-Spcas9-tCYC1 natNT1) pgk1Δ gpm1Δ fba1Δ hxk2Δ pfk1Δ adh1Δ pdc1Δ eno2Δ can1::(YlFBA$_{BA}$YlPGI$_{BI}$YlPGK$_{BJ}$YlTDH1$_{BE}$YlENO$_{BF}$ScADH1$_{BC}$ ScPDC1$_{BD}$YlGPM$_{BK}$YlTPI) X2::GroEL$_{AA}$GroES spr3::(YlPYK$_{BG}$YlPFK$_{BH}$YlGLK)*	This study

IMX2363	Deletion SinLoG *sga1*	*MATa ura3-52 his3-1 leu2-3,112 MAL2-8c SUC2 glk1Δ:: (pAgTEF1-SpHIS5-tAgTEF1) hxk1Δ::KlLEU2 tdh1Δ tdh2Δ gpm2Δ gpm3Δ eno1Δ pyk2Δ pdc5Δ pdc6Δ adh2Δ adh5Δ adh4Δ sga1Δ pyk1Δ pgi1Δ tpi1Δ tdh3Δ pfk2Δ::(pTEF1-Spcas9-tCYC1 natNT1) pgk1Δ gpm1Δ fba1Δ hxk2Δ pfk1Δ adh1Δ pdc1Δ eno2Δ can1::(YlFBA$_{BA}$YlPGI$_{BI}$YlPGK$_{BJ}$YlTDH1$_{BE}$YlENO$_{BF}$ScADH1$_{BC}$ ScPDC1$_{BD}$YlGPM$_{BK}$YlTPI) X2::GroEL$_{AA}$GroES spr3::(YlPYK$_{BG}$YlPFK$_{BH}$YlGLK)*	This study
IMX2417	*URA3* repair	*MATa URA3 his3-1 leu2-3,112 MAL2-8c SUC2 glk1Δ:: (pAgTEF1-SpHIS5-tAgTEF1) hxk1Δ::KlLEU2 tdh1Δ tdh2Δ gpm2Δ gpm3Δ eno1Δ pyk2Δ pdc5Δ pdc6Δ adh2Δ adh5Δ adh4Δ sga1Δ pyk1Δ pgi1Δ tpi1Δ tdh3Δ pfk2Δ::(pTEF1-Spcas9-tCYC1 natNT1) pgk1Δ gpm1Δ fba1Δ hxk2Δ pfk1Δ adh1Δ pdc1Δ eno2Δ can1::(YlFBA$_{BA}$YlPGI$_{BI}$YlPGK$_{BJ}$YlTDH1$_{BE}$YlENO$_{BF}$ScADH1$_{BC}$ ScPDC1$_{BD}$YlGPM$_{BK}$YlTPI) X2::GroEL$_{AA}$GroES spr3::(YlPYK$_{BG}$YlPFK$_{BH}$YlGLK)*	This study

Table S1D - *Yarrowia* single and double gene complementation strains

**Strain**	**Description of modification**	**Genotype**	**Reference**
IMX2047	*YlHXK1* integration	*MATa his3-1 leu2-3,112 MAL2-8c SUC2 glk1Δ:: (pAgTEF1-SpHIS5-tAgTEF1) hxk1Δ::KlLEU2 tdh1Δ tdh2Δ gpm2Δ gpm3Δ eno1Δ pyk2Δ pdc5Δ pdc6Δ adh2Δ adh5Δ adh4Δ, sga1::(cas9 natNT1) ura3 ::(YlHXK1 URA3)*	This study
IMX2048	*YlGLK1* integration	*MATa his3-1 leu2-3,112 MAL2-8c SUC2 glk1Δ:: (pAgTEF1-SpHIS5-tAgTEF1) hxk1Δ::KlLEU2 tdh1Δ tdh2Δ gpm2Δ gpm3Δ eno1Δ pyk2Δ pdc5Δ pdc6Δ adh2Δ adh5Δ adh4Δ, sga1::(cas9 natNT1) ura3 ::(YlGLK1 URA3)*	This study
IMX2049	*YlPYK1* integration	*MATa his3-1 leu2-3,112 MAL2-8c SUC2 glk1Δ:: (pAgTEF1-SpHIS5-tAgTEF1) hxk1Δ::KlLEU2 tdh1Δ tdh2Δ gpm2Δ gpm3Δ eno1Δ pyk2Δ pdc5Δ pdc6Δ adh2Δ adh5Δ adh4Δ, sga1::(cas9 natNT1) ura3 ::(YlPYK1 URA3)*	This study
IMX2050	*YlPFK1* integration	*MATa his3-1 leu2-3,112 MAL2-8c SUC2 glk1Δ:: (pAgTEF1-SpHIS5-tAgTEF1) hxk1Δ::KlLEU2 tdh1Δ tdh2Δ gpm2Δ gpm3Δ eno1Δ pyk2Δ pdc5Δ pdc6Δ adh2Δ adh5Δ adh4Δ, sga1::(cas9 natNT1) ura3 ::(YlPFK1 URA3)*	This study
IMX2061	*ScHXK2* deletion	*MATa his3-1 leu2-3,112 MAL2-8c SUC2 glk1Δ:: (pAgTEF1-SpHIS5-tAgTEF1) hxk1Δ::KlLEU2 tdh1Δ tdh2Δ gpm2Δ gpm3Δ eno1Δ pyk2Δ pdc5Δ pdc6Δ adh2Δ adh5Δ adh4Δ, sga1::(cas9 natNT1) ura3 ::(YlHXK1 URA3) hxk2Δ*	This study
IMX2062	*ScHXK2* deletion	*MATa his3-1 leu2-3,112 MAL2-8c SUC2 glk1Δ:: (pAgTEF1-SpHIS5-tAgTEF1) hxk1Δ::KlLEU2 tdh1Δ tdh2Δ gpm2Δ gpm3Δ eno1Δ pyk2Δ pdc5Δ pdc6Δ adh2Δ adh5Δ adh4Δ, sga1::(cas9 natNT1) ura3 ::(YlGLK1 URA3) hxk2Δ*	This study
IMX2235	*ScPFK1* and *ScPFK2* deletion	*MATa his3-1 leu2-3,112 MAL2-8c SUC2 glk1Δ:: (pAgTEF1-SpHIS5-tAgTEF1) hxk1Δ::KlLEU2 tdh1Δ tdh2Δ gpm2Δ gpm3Δ eno1Δ pyk2Δ pdc5Δ pdc6Δ adh2Δ adh5Δ adh4Δ, sga1::(cas9 natNT1) ura3 ::(YlPYK1 URA3) pyk1Δ*	This study
IMX2236	*ScPYK1* deletion	*MATa his3-1 leu2-3,112 MAL2-8c SUC2 glk1Δ:: (pAgTEF1-SpHIS5-tAgTEF1) hxk1Δ::KlLEU2 tdh1Δ tdh2Δ gpm2Δ gpm3Δ eno1Δ pyk2Δ pdc5Δ pdc6Δ adh2Δ adh5Δ adh4Δ, sga1::(cas9 natNT1) ura3 ::(YlPFK1 URA3) pfk1Δ pfk2Δ*	This study
IMX2549	*URA3* deletion	*MATa his3-1 leu2-3,112 MAL2-8c SUC2 glk1Δ:: (pAgTEF1-SpHIS5-tAgTEF1) hxk1Δ::KlLEU2 tdh1Δ tdh2Δ gpm2Δ gpm3Δ*	This study

		eno1Δ pyk2Δ pdc5Δ pdc6Δ adh2Δ adh5Δ adh4Δ sga1::(cas9 natNT1) ura3 ::(YlHXK1 ura3) hxk2Δ	
IMX2550	*URA3* deletion	MATa his3-1 leu2-3,112 MAL2-8c SUC2 glk1Δ:: (pAgTEF1-SpHIS5-tAgTEF1) hxk1Δ::KlLEU2 tdh1Δ tdh2Δ gpm2Δ gpm3Δ eno1Δ pyk2Δ pdc5Δ pdc6Δ adh2Δ adh5Δ adh4Δ sga1::(cas9 natNT1) ura3 ::(YlGLK1 ura3) hxk2Δ	This study
IMX2551	*URA3* deletion	MATa his3-1 leu2-3,112 MAL2-8c SUC2 glk1Δ:: (pAgTEF1-SpHIS5-tAgTEF1) hxk1Δ::KlLEU2 tdh1Δ tdh2Δ gpm2Δ gpm3Δ eno1Δ pyk2Δ pdc5Δ pdc6Δ adh2Δ adh5Δ adh4Δ sga1::(cas9 natNT1) ura3 ::(YlPYK1 ura3) pyk1Δ	This study
IMX2552	*URA3* deletion	MATa his3-1 leu2-3,112 MAL2-8c SUC2 glk1Δ:: (pAgTEF1-SpHIS5-tAgTEF1) hxk1Δ::KlLEU2 tdh1Δ tdh2Δ gpm2Δ gpm3Δ eno1Δ pyk2Δ pdc5Δ pdc6Δ adh2Δ adh5Δ adh4Δ sga1::(cas9 natNT1) ura3 ::(YlPFK1 ura3) pfk1Δ pfk2Δ	This study
IMX2812	*ScHXK2* deletion	MATa his3-1 leu2-3,112 MAL2-8c SUC2 glk1Δ:: (pAgTEF1-SpHIS5-tAgTEF1) hxk1Δ::KlLEU2 tdh1Δ tdh2Δ gpm2Δ gpm3Δ eno1Δ pyk2Δ pdc5Δ pdc6Δ adh2Δ adh5Δ adh4Δ sga1::(cas9 natNT1) ura3 ::(YlPYK1 URA3) pyk1Δ hxk2Δ	This study
IMX2842	*YlGLK1* integration	MATa his3-1 leu2-3,112 MAL2-8c SUC2 glk1Δ:: (pAgTEF1-SpHIS5-tAgTEF1) hxk1Δ::KlLEU2 tdh1Δ tdh2Δ gpm2Δ gpm3Δ eno1Δ pyk2Δ pdc5Δ pdc6Δ adh2Δ adh5Δ adh4Δ sga1::(cas9 natNT1) ura3 ::(YlPYK1 URA3) pyk1Δ hxk2Δ X2::YlGLK1	This study

Table S1E - Mosaic *Yarrowia* and *Saccharomyces* glycolysis strains

Strain	Description of modification	Genotype	Reference
IMX1751	Mosaic SinLoG with *Sc* kinases integrated	MATa ura3-52 his3-1 leu2-3,112 MAL2-8c SUC2 glk1Δ:: (pAgTEF1-SpHIS5-tAgTEF1) hxk1Δ::KlLEU2 tdh1Δ tdh2Δ gpm2Δ gpm3Δ eno1Δ pyk2Δ pdc5Δ pdc6Δ adh2Δ adh5Δ adh4Δ sga1Δ::($_{G}$ tFBA1-FBA1-pFBA1 $_{H}$ pTPI1-TPI1-tTPI1 $_{P}$ tPGK1-PGK1-pPGK1 $_{Q}$ tADH1-ADH1-pADH1 $_{N}$ pPYK1-PYK1-tPYK1 $_{O}$ tTDH3-TDH3-pTDH3 $_{A}$ pENO2-ENO2-tENO2 $_{B}$ pHXK2-HXK2-tHXK2 $_{C}$ pPGI-PGI1-tPGI1 $_{D}$ pPFK1-PFK1-tPFK1 $_{J}$ tPFK2-PFK2-pPFK2 $_{K}$ pAgTEF1-AmdSYM-tAgTEF1 $_{L}$ tGPM1-GPM1-pGPM1 $_{M}$ pPDC1-PDC1-tPDC1-SYN $_{F}$) pyk1Δ pgi1Δ tpi1Δ tdh3Δ pfk2Δ::(pTEF1-Spcas9-tCYC1 natNT1) pgk1Δ gpm1Δ fba1Δ hxk2Δ pfk1Δ adh1Δ pdc1Δ eno2Δ can1::(YlFBA$_{BA}$YlPGI$_{BI}$YlPGK$_{BG}$ScPFK1$_{BH}$ScPYK1$_{BJ}$YlTDH1$_{BE}$ YlENO$_{BF}$ScADH1$_{BB}$ScHXK2$_{BC}$ScPDC1$_{BD}$YlGPM$_{BK}$YlTPI$_{BL}$ScPFK2)	This study
IMX1803	SinLoG native genes deleted	MATa ura3-52 his3-1 leu2-3,112 MAL2-8c SUC2 glk1Δ:: (pAgTEF1-SpHIS5-tAgTEF1) hxk1Δ::KlLEU2 tdh1Δ tdh2Δ gpm2Δ gpm3Δ eno1Δ pyk2Δ pdc5Δ pdc6Δ adh2Δ adh5Δ adh4Δ sga1Δ pyk1Δ pgi1Δ tpi1Δ tdh3Δ pfk2Δ::(pTEF1-Spcas9-tCYC1 natNT1) pgk1Δ gpm1Δ fba1Δ hxk2Δ pfk1Δ adh1Δ pdc1Δ eno2Δ can1::(YlFBA$_{BA}$YlPGI$_{BI}$YlPGK$_{BG}$ScPFK1$_{BH}$ScPYK1$_{BJ}$YlTDH1$_{BE}$ YlENO$_{BF}$ScADH1$_{BB}$ScHXK2$_{BC}$ScPDC1$_{BD}$YlGPM$_{BK}$YlTPI$_{BL}$ScPFK2)	This study
IMX2465	*URA3* repaired	MATa URA3 his3-1 leu2-3,112 MAL2-8c SUC2 glk1Δ:: (pAgTEF1-SpHIS5-tAgTEF1) hxk1Δ::KlLEU2 tdh1Δ tdh2Δ gpm2Δ gpm3Δ eno1Δ pyk2Δ pdc5Δ pdc6Δ adh2Δ adh5Δ adh4Δ sga1Δ pyk1Δ pgi1Δ tpi1Δ tdh3Δ pfk2Δ::(pTEF1-Spcas9-tCYC1 natNT1) pgk1Δ gpm1Δ fba1Δ hxk2Δ pfk1Δ	This study

		adh1Δ pdc1Δ eno2Δ can1::(YlFBA$_{BA}$YlPGI$_{BI}$YlPGK$_{BG}$ScPFK1$_{BH}$ScPYK1$_{BJ}$YlTDH1$_{BE}$ YlENO$_{BF}$ScADH1$_{BB}$ScHXK2$_{BC}$ScPDC1$_{BD}$YlGPM$_{BK}$YlTPI$_{BL}$ScPFK2)	
IMX2697	GroEL/ES integrated	MATa URA3 his3-1 leu2-3,112 MAL2-8c SUC2 glk1Δ:: (pAgTEF1-SpHIS5-tAgTEF1) hxk1Δ::KlLEU2 tdh1Δ tdh2Δ gpm2Δ gpm3Δ eno1Δ pyk2Δ pdc5Δ pdc6Δ adh2Δ adh5Δ adh4Δ sga1Δ pyk1Δ pgi1Δ tpi1Δ tdh3Δ pfk2Δ::(pTEF1-Spcas9-tCYC1 natNT1) pgk1Δ gpm1Δ fba1Δ hxk2Δ pfk1Δ adh1Δ pdc1Δ eno2Δ can1::(YlFBA$_{BA}$YlPGI$_{BI}$YlPGK$_{BG}$ScPFK1$_{BH}$ScPYK1$_{BJ}$YlTDH1$_{BE}$ YlENO$_{BF}$ScADH1$_{BB}$ScHXK2$_{BC}$ScPDC1$_{BD}$YlGPM$_{BK}$YlTPI$_{BL}$ScPFK2) X2::GroEL$_{AA}$GroES	This study
IMX2703	SinLoG with *Yl* kinases integrated	MATa ura3-52 his3-1 leu2-3,112 MAL2-8c SUC2 glk1Δ:: (pAgTEF1-SpHIS5-tAgTEF1) hxk1Δ::KlLEU2 tdh1Δ tdh2Δ gpm2Δ gpm3Δ eno1Δ pyk2Δ pdc5Δ pdc6Δ adh2Δ adh5Δ adh4Δ sga1Δ::(FBA1 $_{H}$ TPI1 $_{P}$ PGK1 $_{Q}$ ADH1 $_{N}$ PYK1 $_{O}$ TDH3 $_{A}$ ENO2 $_{B}$ HXK2 $_{C}$ PGI1 $_{D}$ PFK1 $_{J}$ PFK2 $_{K}$ AmdSYM $_{L}$ GPM1 $_{M}$ PDC1 SYN $_{F}$) pyk1Δ pgi1Δ tpi1Δ tdh3Δ pfk2Δ::(Spcas9 natNT1) pgk1Δ gpm1Δ fba1Δ hxk2Δ pfk1Δ adh1Δ pdc1Δ eno2Δ X2::GroEL$_{AA}$GroES can1 Δ::(ScFBA1$_{BA}$ ScPGI1 $_{BI}$ ScPGK1 $_{BG}$ YlPFK1 $_{BH}$ YlPYK1 $_{BJ}$ ScTDH3 $_{BE}$ ScENO2 $_{BF}$ ScADH1 $_{BB}$ YlGLK1 $_{BC}$ ScPDC1 $_{BD}$ ScGPM1 $_{BK}$ ScTPI1)	This study
IMX2718	*Yl*-3K strain deletion native SinLoG	MATa ura3-52 his3-1 leu2-3,112 MAL2-8c SUC2 glk1Δ:: (pAgTEF1-SpHIS5-tAgTEF1) hxk1Δ::KlLEU2 tdh1Δ tdh2Δ gpm2Δ gpm3Δ eno1Δ pyk2Δ pdc5Δ pdc6Δ adh2Δ adh5Δ adh4Δ sga1Δ pyk1Δ pgi1Δ tpi1Δ tdh3Δ pfk2Δ::(Spcas9 natNT1) pgk1Δ gpm1Δ fba1Δ hxk2Δ pfk1Δ adh1Δ pdc1Δ eno2Δ X2::GroEL$_{AA}$GroES can1 Δ::(ScFBA1$_{BA}$ ScPGI1 $_{BI}$ ScPGK1 $_{BG}$ YlPFK1 $_{BH}$ YlPYK1 $_{BJ}$ ScTDH3 $_{BE}$ ScENO2 $_{BF}$ ScADH1 $_{BB}$ YlGLK1 $_{BC}$ ScPDC1 $_{BD}$ ScGPM1 $_{BK}$ ScTPI1)	This study
IMX2733	*Yl*-3K strain *URA3* repair	MATa URA3 his3-1 leu2-3,112 MAL2-8c SUC2 glk1Δ:: (pAgTEF1-SpHIS5-tAgTEF1) hxk1Δ::KlLEU2 tdh1Δ tdh2Δ gpm2Δ gpm3Δ eno1Δ pyk2Δ pdc5Δ pdc6Δ adh2Δ adh5Δ adh4Δ sga1Δ pyk1Δ pgi1Δ tpi1Δ tdh3Δ pfk2Δ::(Spcas9 natNT1) pgk1Δ gpm1Δ fba1Δ hxk2Δ pfk1Δ adh1Δ pdc1Δ eno2Δ X2::GroEL$_{AA}$GroES can1 Δ::(ScFBA1$_{BA}$ ScPGI1 $_{BI}$ ScPGK1 $_{BG}$ YlPFK1 $_{BH}$ YlPYK1 $_{BJ}$ ScTDH3 $_{BE}$ ScENO2 $_{BF}$ ScADH1 $_{BB}$ YlGLK1 $_{BC}$ ScPDC1 $_{BD}$ ScGPM1 $_{BK}$ ScTPI1)	This study
IMX2164	Integration of key-point genes in *SPR3*	MATa ura3-52 his3-1 leu2-3,112 MAL2-8c SUC2 glk1Δ:: (pAgTEF1-SpHIS5-tAgTEF1) hxk1Δ::KlLEU2 tdh1Δ tdh2Δ gpm2Δ gpm3Δ eno1Δ pyk2Δ pdc5Δ pdc6Δ adh2Δ adh5Δ adh4Δ sga1Δ::($_{G}$ tFBA1-FBA1-pFBA1 $_{H}$ pTPI1-TPI1-tTPI1 $_{P}$ tPGK1-PGK1-pPGK1 $_{Q}$ tADH1-ADH1-pADH1 $_{N}$ pPYK1-PYK1-tPYK1 $_{O}$ tTDH3-TDH3-pTDH3 $_{A}$ pENO2-ENO2-tENO2 $_{B}$ pHXK2-HXK2-tHXK2 $_{C}$ pPGI-PGI1-tPGI1 $_{D}$ pPFK1-PFK1-tPFK1 $_{J}$ tPFK2-PFK2-pPFK2 $_{K}$ $_{L}$ tGPM1-GPM1-pGPM1 $_{M}$ pPDC1-PDC1-tPDC1-SYN $_{F}$) pyk1Δ pgi1Δ tpi1Δ tdh3Δ pfk2Δ::(pTEF1-Spcas9-tCYC1 natNT1) pgk1Δ gpm1Δ fba1Δ hxk2Δ pfk1Δ adh1Δ pdc1Δ eno2Δ can1::(YlFBA$_{BA}$YlPGI$_{BI}$ YlPGK$_{BJ}$YlTDH1$_{BE}$YlENO$_{BF}$ScADH1$_{BC}$ ScPDC1$_{BD}$YlGPM$_{BK}$YlTPI) X2::GroEL$_{AA}$GroES spr3::(YlPYK$_{BG}$ScPFK1$_{BH}$YlGLK$_{BL}$ScPFK2)	This study
IMX2182	Deletion native SinLoG	MATa ura3-52 his3-1 leu2-3,112 MAL2-8c SUC2 glk1Δ:: (pAgTEF1-SpHIS5-tAgTEF1) hxk1Δ::KlLEU2 tdh1Δ tdh2Δ gpm2Δ gpm3Δ eno1Δ pyk2Δ pdc5Δ pdc6Δ adh2Δ adh5Δ adh4Δ pyk1Δ pgi1Δ tpi1Δ tdh3Δ pfk2Δ::(pTEF1-Spcas9-tCYC1 natNT1) pgk1Δ gpm1Δ fba1Δ hxk2Δ pfk1Δ adh1Δ pdc1Δ eno2Δ can1::(YlFBA$_{BA}$YlPGI$_{BI}$YlPGK$_{BJ}$YlTDH1$_{BE}$	This study

		YlENO$_{BF}$ScADH1$_{BC}$ ScPDC1$_{BD}$YlGPM$_{BK}$YlTPI) X2::GroEL$_{AA}$GroES spr3::(YlPYK$_{BG}$ScPFK1$_{BH}$YlGLK$_{BL}$ScPFK2)	
IMX2734	*URA3* repair	MATa URA3 his3-1 leu2-3,112 MAL2-8c SUC2 glk1Δ:: (pAgTEF1-SpHIS5-tAgTEF1) hxk1Δ::KlLEU2 tdh1Δ tdh2Δ gpm2Δ gpm3Δ eno1Δ pyk2Δ pdc5Δ pdc6Δ adh2Δ adh5Δ adh4Δ pyk1Δ pgi1Δ tpi1Δ tdh3Δ pfk2Δ::(pTEF1-Spcas9-tCYC1 natNT1) pgk1Δ gpm1Δ fba1Δ hxk2Δ pfk1Δ adh1Δ pdc1Δ eno2Δ can1::(YlFBA$_{BA}$YlPGI$_{BI}$YlPGK$_{BJ}$YlTDH1$_{BE}$ YlENO$_{BF}$ScADH1$_{BC}$ ScPDC1$_{BD}$YlGPM$_{BK}$YlTPI) X2::GroEL$_{AA}$GroES spr3::(YlPYK$_{BG}$ScPFK1$_{BH}$YlGLK$_{BL}$ScPFK2)	This study

Table S1F - pHluorin expressing strains

**Strain**	**Description of strain**	**Genotype**	**Reference**
IME480	CEN.PK control	MATa URA3 TRP1 LEU2 HIS3 MAL2-8c SUC2	This study
IME481	SwYG control	MATa ura3-52 his3-1 leu2-3,112 MAL2-8c SUC2 glk1Δ:: (pAgTEF1-SpHIS5-tAgTEF1) hxk1Δ::KlLEU2 tdh1Δ tdh2Δ gpm2Δ gpm3Δ eno1Δ pyk2Δ pdc5Δ pdc6Δ adh2Δ adh5Δ adh4Δ sga1Δ::(FBA1 $_{H}$ TPI1 $_{P}$ PGK1 $_{Q}$ ADH1 $_{N}$ PYK1 $_{O}$ TDH3 $_{A}$ ENO2 $_{B}$ HXK2 $_{C}$ PGI1 $_{D}$ PFK1 $_{J}$ PFK2 $_{K}$ AmdSYM $_{L}$ GPM1 $_{M}$ PDC1 SYN $_{F}$) pyk1Δ pgi1Δ tpi1Δ tdh3Δ pfk2Δ::(Spcas9 natNT1) pgk1Δ gpm1Δ fba1Δ hxk2Δ pfk1Δ adh1Δ pdc1Δ eno2Δ pYES2-P$_{ACT1}$-pHluorin	This study
IME576	*tps1* strain	MATa ura3-52 TRP1 LEU2 HIS3 MAL2-8c SUC2 can1Δ::cas9-natNT2 tps1Δ	This study
IME577	*Yl*-Glycolysis strain	MATa ura3-52 his3-1 leu2-3,112 MAL2-8c SUC2 glk1Δ:: (pAgTEF1-SpHIS5-tAgTEF1) hxk1Δ::KlLEU2 tdh1Δ tdh2Δ gpm2Δ gpm3Δ eno1Δ pyk2Δ pdc5Δ pdc6Δ adh2Δ adh5Δ adh4Δ sga1Δ pyk1Δ pgi1Δ tpi1Δ tdh3Δ pfk2Δ::(pTEF1-Spcas9-tCYC1 natNT1) pgk1Δ gpm1Δ fba1Δ hxk2Δ pfk1Δ adh1Δ pdc1Δ eno2Δ can1::(YlFBA$_{BA}$YlPGI$_{BI}$YlPGK$_{BJ}$YlTDH1$_{BE}$YlENO$_{BF}$ScADH1$_{BC}$ ScPDC1$_{BD}$YlGPM$_{BK}$YlTPI) X2::GroEL$_{AA}$GroES spr3::(YlPYK$_{BG}$YlPFK$_{BH}$YlGLK) pYES2-P$_{ACT1}$-pHluorin	This study
IME579	*Sc*-3K strain	MATa ura3-52 his3-1 leu2-3,112 MAL2-8c SUC2 glk1Δ:: (pAgTEF1-SpHIS5-tAgTEF1) hxk1Δ::KlLEU2 tdh1Δ tdh2Δ gpm2Δ gpm3Δ eno1Δ pyk2Δ pdc5Δ pdc6Δ adh2Δ adh5Δ adh4Δ sga1Δ pyk1Δ pgi1Δ tpi1Δ tdh3Δ pfk2Δ::(pTEF1-Spcas9-tCYC1 natNT1) pgk1Δ gpm1Δ fba1Δ hxk2Δ pfk1Δ adh1Δ pdc1Δ eno2Δ can1::(YlFBA$_{BA}$YlPGI$_{BI}$YlPGK$_{BG}$ScPFK1$_{BH}$ScPYK1$_{BJ}$YlTDH1$_{BE}$ YlENO$_{BF}$ScADH1$_{BB}$ScHXK2$_{BC}$ScPDC1$_{BD}$YlGPM$_{BK}$YlTPI$_{BL}$ScPFK2) pYES2-P$_{ACT1}$-pHluorin	This study
IME683	*Yl*-3K strain	MATa ura3-52 his3-1 leu2-3,112 MAL2-8c SUC2 glk1Δ:: (pAgTEF1-SpHIS5-tAgTEF1) hxk1Δ::KlLEU2 tdh1Δ tdh2Δ gpm2Δ gpm3Δ eno1Δ pyk2Δ pdc5Δ pdc6Δ adh2Δ adh5Δ adh4Δ sga1Δ pyk1Δ pgi1Δ tpi1Δ tdh3Δ pfk2Δ::(Spcas9 natNT1) pgk1Δ gpm1Δ fba1Δ hxk2Δ pfk1Δ adh1Δ pdc1Δ eno2Δ X2::GroEL$_{AA}$GroES can1 Δ::(ScFBA1$_{BA}$ ScPGI1 $_{BI}$ ScPGK1 $_{BG}$ YlPFK1 $_{BH}$ YlPYK1 $_{BJ}$ ScTDH3 $_{BE}$ ScENO2 $_{BF}$ ScADH1 $_{BB}$ YlGLK1 $_{BC}$ ScPDC1 $_{BD}$ ScGPM1 $_{BK}$ ScTPI1) pYES2-P$_{ACT1}$-pHluorin	This study
IME609	*Yl*-Glycolysis, *ScPFKs*	MATa ura3-52 his3-1 leu2-3,112 MAL2-8c SUC2 glk1Δ:: (pAgTEF1-SpHIS5-tAgTEF1) hxk1Δ::KlLEU2 tdh1Δ tdh2Δ gpm2Δ gpm3Δ eno1Δ pyk2Δ pdc5Δ pdc6Δ adh2Δ adh5Δ	

		adh4Δ pyk1Δ pgi1Δ tpi1Δ tdh3Δ pfk2Δ::(pTEF1-Spcas9-tCYC1 natNT1) pgk1Δ gpm1Δ fba1Δ hxk2Δ pfk1Δ adh1Δ pdc1Δ eno2Δ can1::(YlFBA$_{BA}$YlPGI$_{BI}$YlPGK$_{BJ}$YlTDH1$_{BE}$ YlENO$_{BF}$ScADH1$_{BC}$ ScPDC1$_{BD}$YlGPM$_{BK}$YlTPI) X2::GroEL$_{AA}$GroES spr3::(YlPYK$_{BG}$ScPFK1$_{BH}$YlGLK$_{BL}$ScPFK2) pYES2-P$_{ACT1}$-pHluorin	
IME627	*YlHXK* complementation	*MATa his3-1 leu2-3,112 MAL2-8c SUC2 glk1Δ:: (pAgTEF1-SpHIS5-tAgTEF1) hxk1Δ::KlLEU2 tdh1Δ tdh2Δ gpm2Δ gpm3Δ eno1Δ pyk2Δ pdc5Δ pdc6Δ adh2Δ adh5Δ adh4Δ, sga1::(cas9 natNT1) ura3 ::(YlHXK1 ura3) hxk2Δ pYES2-P$_{ACT1}$-pHluorin*	This study
IME628	*YlGLK* complementation	*MATa his3-1 leu2-3,112 MAL2-8c SUC2 glk1Δ:: (pAgTEF1-SpHIS5-tAgTEF1) hxk1Δ::KlLEU2 tdh1Δ tdh2Δ gpm2Δ gpm3Δ eno1Δ pyk2Δ pdc5Δ pdc6Δ adh2Δ adh5Δ adh4Δ, sga1::(cas9 natNT1) ura3 ::(YlGLK1 ura3) hxk2Δ pYES2-P$_{ACT1}$-pHluorin*	This study
IME631	*YlPFK* complementation	*MATa his3-1 leu2-3,112 MAL2-8c SUC2 glk1Δ:: (pAgTEF1-SpHIS5-tAgTEF1) hxk1Δ::KlLEU2 tdh1Δ tdh2Δ gpm2Δ gpm3Δ eno1Δ pyk2Δ pdc5Δ pdc6Δ adh2Δ adh5Δ adh4Δ, sga1::(cas9 natNT1) ura3 ::(YlPFK1 ura3) pfk1Δ pfk2Δ pYES2-P$_{ACT1}$-pHluorin*	This study
IME632	*YlPYK* complementation	*MATa his3-1 leu2-3,112 MAL2-8c SUC2 glk1Δ:: (pAgTEF1-SpHIS5-tAgTEF1) hxk1Δ::KlLEU2 tdh1Δ tdh2Δ gpm2Δ gpm3Δ eno1Δ pyk2Δ pdc5Δ pdc6Δ adh2Δ adh5Δ adh4Δ, sga1::(cas9 natNT1) ura3 ::(YlPYK1 ura3) pyk1Δ pYES2-P$_{ACT1}$-pHluorin*	This study

Table S1G - Evolved strains

Strain	Description of strain	Genotype	Reference
IMS1203	Single colony isolates after growth on glucose of *Yl*-Glycolysis strain IMX2417	*MATa URA3 his3-1 leu2-3,112 MAL2-8c SUC2 glk1Δ:: (pAgTEF1-SpHIS5-tAgTEF1) hxk1Δ::KlLEU2 tdh1Δ tdh2Δ gpm2Δ gpm3Δ eno1Δ pyk2Δ pdc5Δ pdc6Δ adh2Δ adh5Δ adh4Δ sga1Δ pyk1Δ pgi1Δ tpi1Δ tdh3Δ pfk2Δ::(pTEF1-Spcas9-tCYC1 natNT1) pgk1Δ gpm1Δ fba1Δ hxk2Δ pfk1Δ adh1Δ pdc1Δ eno2Δ can1::(YlFBA$_{BA}$YlPGI$_{BI}$YlPGK$_{BJ}$YlTDH1$_{BE}$YlENO$_{BF}$ScADH1$_{BC}$ ScPDC1$_{BD}$YlGPM$_{BK}$YlTPI) X2::GroEL$_{AA}$GroES spr3::(YlPYK$_{BG}$YlPFK$_{BH}$YlGLK)*	This study
IMS1204			
IMS1205			
IMS1207	Single colony isolates after growth on glucose of *Yl*-3K strain IMX2733	*MATa URA3 his3-1 leu2-3,112 MAL2-8c SUC2 glk1Δ:: (pAgTEF1-SpHIS5-tAgTEF1) hxk1Δ::KlLEU2 tdh1Δ tdh2Δ gpm2Δ gpm3Δ eno1Δ pyk2Δ pdc5Δ pdc6Δ adh2Δ adh5Δ adh4Δ sga1Δ pyk1Δ pgi1Δ tpi1Δ tdh3Δ pfk2Δ::(Spcas9 natNT1) pgk1Δ gpm1Δ fba1Δ hxk2Δ pfk1Δ adh1Δ pdc1Δ eno2Δ X2::GroEL$_{AA}$GroES can1 Δ::(ScFBA1$_{BA}$ ScPGI1 $_{BI}$ ScPGK1 $_{BG}$ YlPFK1 $_{BH}$ YlPYK1 $_{BJ}$ ScTDH3 $_{BE}$ ScENO2 $_{BF}$ ScADH1 $_{BB}$ YlGLK1 $_{BC}$ ScPDC2 $_{BD}$ ScGPM1 $_{BK}$ ScTPI1)*	This study
IMS1208			
IMS1209			
IMS1218	Single colony isolates after growth on glucose of *YlGLK* complementation IMX2062	*MATa his3-1 leu2-3,112 MAL2-8c SUC2 glk1Δ:: (pAgTEF1-SpHIS5-tAgTEF1) hxk1Δ::KlLEU2 tdh1Δ tdh2Δ gpm2Δ gpm3Δ eno1Δ pyk2Δ pdc5Δ pdc6Δ adh2Δ adh5Δ adh4Δ, sga1::(cas9 natNT1) ura3 ::(YlGLK1 URA3) hxk2Δ*	This study
IMS1219			
IMS1220			

Table S2 - Genetic composition expression cassettes

Y. lipolytica gene	*S. cerevisiae* promotor	*S. cerevisiae* terminator
HXK1	*HXK2*	*HXK2*
GLK1	*ACT1*	*ENO1*
PFK1	*TEF1*	*TEF1*
PYK1	*PYK1*	*PYK1*
PGI1	*PGI1*	*PGI1*
FBA1	*FBA1*	*FBA1*
TPI1	*TPI1*	*TPI1*
TDH1	*TDH3*	*TDH3*
PGK1	*PGK1*	*PGK1*
GPM1	*GPM1*	*GPM1*
ENO1	*ENO2*	*ENO2*

Table S3 - Plasmids used in this study

Table S3A - Plasmids for Golden Gate assembly

Name	Construct	Source
pUD565	Entry vector, CamR	GeneArt
pGGKd002	GFP dropout integration plasmid	[5]
pGGKp152	*YlPGK1*	This study
pGGKp153	*YlTPI1*	This study
pGGKp155	*YlGPM1*	This study
pGGKp156	*YlFBA1*	This study
pGGKp157	*YlPGI1*	This study
pGGKp159	*YlTDH1*	This study
pGGKp160	*YlENO1*	This study
pGGKp215	*YlGLK1*	GeneArt
pGGKp216	*YlHXK1*	GeneArt
pGGKp217	*YlPFK1*	GeneArt
pGGKp218	*YlPYK1*	GeneArt
pYTK051	*ScENO1t*	[9]
pYTK056	*ScTDH1t*	[9]
pYTK074	*URA3*	[9]
pGGKp026	ScGPM1p	[5]
pGGKp027	ScFBA1p	[5]
pGGKp028	ScENO2p	[5]
pGGKp030	ScTPI1p	[5]
pGGKp032	*ScTEF1p*	[5]
pGGKp033	ScPGI1p	[5]
pGGKp034	*ScPYK1p*	[5]
pGGKp035	ScTDH3p	[5]
pGGKp036	ScPGK1p	[5]
pGGKp039	*ScTEF1t*	[5]
pGGKp040	*ScPYK1t*	[5]
pGGKp041	ScTDH3t	[5]
pGGKp042	ScTPI1t	[5]
pGGKp043	ScPGK1t	[5]
pGGKp044	ScPGI1t	[5]
pGGKp046	ScFBA1t	[5]
pGGKp047	*ScACT1p*	[5]
pGGKp048	*ScGPM1t*	[5]
pGGKp096	*ScHXK2p*	[5]
pGGKp097	*ScHXK2t*	[5]

Table S3B - Expression cassette plasmids

Name	Construct	Source
pUDE739	*ScFBA1p-YlFBA1-ScFBA1t*	This study
pUDE742	*ScPGK1p-YlPGK1-ScPGK1t*	This study
pUDE744	*ScENO2p-YlENO1-ScENO2t*	This study
pUDE745	*ScPGI1p-YlPGI1-ScPGI1t*	This study
pUDE746	*ScTDH3p-YlTDH1-ScTDH3t*	This study
pUDE747	*ScTPI1p-YlTPI1-ScTPI1t*	This study
pUDE748	*ScGPM1p-YlGPM1-ScGPM1t*	This study
pUDI225	*ScHXK2p-YlHXK1-ScHXK2t*	This study
pUDI226	*ScACTp-YlGLK1-ScENO1t*	This study
pUDI227	*ScTEF1p-YlPFK-ScTEF1t*	This study
pUDI228	*ScPYK1p-YlPYK1-ScPYK1t*	This study
pUDE767	*ScHXK*	This study
pUDE768	ScPGI	This study
pUDE769	ScPFK1	This study
pUDE770	ScPFK2	This study
pUDE771	ScFBA1	This study
pUDE772	ScTPI1	This study
pUDE773	ScTDH3	This study
pUDE774	ScPGK1	This study
pUDE775	ScGPM1	This study
pUDE776	ScENO2	This study
pUDE777	ScPYK1	This study
pUDE778	ScPDC1	This study
pUDE779	ScADH1	This study

Table S3C - gRNA plasmids

Name	Relevant characteristics	Source
pMEL13	2µ, ampR, *KanMX*, gRNA-*CAN1*	[7]
pUDR591	2µ, ampR, *KanMX*, gRNA-*URA3* and gRNA-X2	This study
pUDR596	2µ, ampR, *URA3*, gRNA-*SPR3*	This study
pUDE342	2µ, ampR, *URA3, gRNA-CAN1* flanks	[4]
pUDR265	2µ, ampR, *KanMX*, gRNA-*PFK1* and gRNA-*PFK2*	[5]
pUDR371	2µ, ampR, *KanMX*, gRNA-*HXK2*	[5]
pUDR107	2µ, ampR, *hphNT1*, gRNA-*URA3*	[10]
pUDR547	2µ, ampR, *hphNT1*, gRNA-X2	[11]
pUDR626	2µ, ampR, *KanMX*, gRNA-*TPS1*	This study

Table S3D - Other plasmids

Name	Relevant characteristics	Source
pUDE232	*pTEF1-EcgroEL-tACT1*	[12]
pUDE233	*pTPI1-EcgroES-tPGI1*	[12]
pYES2-P_{ACT1}-pHluorin	pHluorin expression cassette *pACT1*-pHluorin	[13]

Table S4 - Primers

Table S4A - Primers used to amplify *Y. lipolytica* genes for part plasmid assembly

Fragment	Primer name	Sequence
YlPGI	12339 YTK_YlPGI_FW	GCATCGTCTCATCGGTCTCATATGGCTCAGTCCTTCACGAC
	12340 YTK_YlPGI_REV	ATGCCGTCTCAGGTCTCAGGATTCAAGCGGCCCAAGCC
YlFBA	12343 YTK_YlFBA_FW	GCATCGTCTCATCGGTCTCATATGCCTGTTACTGACGTCCTTAAG
	12344 YTK_YlFBA_REV	ATGCCGTCTCAGGTCTCAGGATTTACAAGGTGTTCTTGGCGTTG
YlTPI	12345 YTK_YlTPI_FW	GCATCGTCTCATCGGTCTCATATGTCTCGAACCTTTTTTGTTGGCGG
	12346 YTK_YlTPI_REV	ATGCCGTCTCAGGTCTCAGGATTTAAAGTCGAGAGTTGATGATG
YlTDH	12347 YTK_YlTDH3_FW	GCATCGTCTCATCGGTCTCATATGGCCATCAAAGTCGGTATTAAC
	12348 YTK_YlTDH3_REV	ATGCCGTCTCAGGTCTCAGGATCTAAGCGGAAGCATCCTTCTTG
YlPGK	12349 YTK_YlPGK_FW	GCATCGTCTCATCGGTCTCATATGTCTCTTACCAACAAGCTCTC
	12350 YTK_YlPGK_REV	ATGCCGTCTCAGGTCTCAGGATTTACTTCTTCTCGGAGAGAGC
YlGPM	12351 YTK_YlGPM_FW	GCATCGTCTCATCGGTCTCATATGCCTAAACTGATTCTGCTGC
	12352 YTK_YlGPM_REV	ATGCCGTCTCAGGTCTCAGGATTTACTTCTTACCCTGGTTGGCAAC
YlENO	12353 YTK_YlENO_FW	GCATCGTCTCATCGGTCTCATATGCCTGTTGAGAAGCTCCAC
	12354 YTK_YlENO_REV	ATGCCGTCTCAGGTCTCAGGATTTAGATGGCTCGAGAAAGGTG

Table S4B - Primers used to contruct *Y. lipolytica* glycolytic expression cassettes

Fragment	Primer name	Sequence
pGGKd017 backbone	12377 Backbone pGGKd017 FW	AAATCTGCTCGTCAGTGGTG
	12378 Backbone pGGKd017 REV	ATTGCGACGAATTGCCACG
ScENO2p	12379 pENO2 FW	GCGATCACAGACATTAACCCACAGTACAGACACTGCGACAAC GTGGCAATTCGTCGCAATAACGGGATGATGAAAACACTAAAC
	6340 ENO2_P_RV	TATTATTGTATGTTATAGTATTAGTTGCTTGGTGTTATG
YlENO1	12380 YlENO FW	TTTTCTTTTCTTAGTTTCTTTCATAACACCAAGCAACTAATACT ATAACATACAATAATAATGCCTGTTGAGAAGCTCCAC
	12381 YlENO REV	TATGATGAAAAAATAAGCAGAAAAGACTAATAATTCTTAGTT AAAAGCACTCTCGAGTTATTAGATGGCTCGAGAAAGGTGG
ScENO2t	12382 tENO2 FW	ATCCTAACTCGAGAGTGCTTTTAAC
	12383 tENO2 REV	CAGTCATCGGTATGATCTGTACATGATTCGTCAGTGTGAGCA CCACTGACGAGCAGATTTCAGCATTTTTCAAACTGCAAATTC
ScPGI1p	12384 pPGI1 FW	GCGATCACAGACATTAACCCACAGTACAGACACTGCGACAA CGTGGCAATTCGTCGCAATAACGTATTCTTAGTGGATAAC
	5925 Primer_pPGI1_rv	TTTTAGGCTGGTATCTTGATTCTAAATCG
YlPGI1	12385 YlPGI FW	TTTAATACATATTCCTCTAGTCTTGCAAAATCGATTTAGAATC AAGATACCAGCCTAAAAATGGCTCAGTCCTTCACGACC
	12386 YlPGI REV	GTATCTTTGCTTATAATATAGCTTTAATGTTCTTTAGGTATAT ATTTAAGAGCGATTTGTTCAAGCGGCCCAAGCCTTGTAC
ScPGI1t	4671 PGI1t-fw	ACAAATCGCTCTTAAATATATACCTAAAGAAC
	12387 tPGI1 REV	CAGTCATCGGTATGATCTGTACATGATTCGTCAGTGTGAGCA CCACTGACGAGCAGATTTCAGCGAAATAGGACCTGATATC
ScTPI1p	12388 pTPI1 FW	GCGATCACAGACATTAACCCACAGTACAGACACTGCGACAAC GTGGCAATTCGTCGCAATAACGACCCAGAGATGTTGTTGTC
	11183 pTPI_RV	TTTTAGTTTATGTATGTGTTTTTTGTAG
YlTPI1	12389 YlTPI FW	TGTATTCTTTTCTTGCTTAAATCTATAACTACAAAAAACACATA CATAAACTAAAATATGTCTCGAACCTTTTTTGTTGG
	12390 YlTPI REV	TTTTACATAACACTAGATATAAAGAAAAGAAGATAATATTTTT ATATAATTATATTAATCTTAAAGTCGAGAGTTGATGATGTC
ScTPI1t	4490 tTPI1 fw	GATTAATATAATTATATAAAAATATTATCTTCTTTTCTTTATATC TAGTGTTATG
	12391 tTPI1 REV	CAGTCATCGGTATGATCTGTACATGATTCGTCAGTGTGAGCAC CACTGACGAGCAGATTTCAGCCGGTACACTTCTGAGTAAC
ScTDH3p	12392 pTDH3 FW	GCGATCACAGACATTAACCCACAGTACAGACACTGCGACAAC GTGGCAATTCGTCGCAATCGAATATATACTAGCGTTGAATG
	3627 pTDH3 rv	TTTGTTTGTTTATGTGTGTTTATTCGAAAC
YlTDH1	12393 YlTDH3 FW	TTTTTTTAGTTTTAAAACACCAAGAACTTAGTTTCGAATAAAC ACACATAAACAAACAAAATGGCCATCAAAGTCGGTATTAAC
	12394 YlTDH3 REV	CTAAGTCATAAAGCTATAAAAAGAAAATTTATTTAAATGCAA GATTTAAAGTAAATTCACCTAAGCGGAAGCATCCTTCTTGG
ScTDH3t	12395 tTDH3 FW	GTGAATTTACTTTAAATCTTGC
	12396 tTDH3 REV	CAGTCATCGGTATGATCTGTACATGATTCGTCAGTGTGAGCA CCACTGACGAGCAGATTTCAGCGTAACTTCAGAATCGTTATC

ScGPM1p	12631 pGPM1 FW	GCGATCACAGACATTAACCCACAGTACAGACACTGCGACAAC GTGGCAATTCGTCGCAATGTGATACTTTGACAGGAGCTATATC
	6344 GPM1_P_FW	TATTGTAATATGTGTGTTTGTTTGGATTATTAAG
YlGPM1	12632 YlGPM FW	TTGTAATTTTTTTTGTAATTATTCTTCTTAATAATCCAAACAAA CACACATATTACAATAATGCCTAAACTGATTCTGCTGC
	12633 YlGPM REV	ATATATTCAGTAAGAAAAATGGAGGGAAAAAGAAATCATCAA ATCATTCATTCTTCAGACTTACTTCTTACCCTGGTTGGCAAC
ScGPM1t	6505 Sc_GPM1_T_RV	GTCTGAAGAATGAATGATTTGATGATTTCTTTT
	12634 tGPM1 REV	CAGTCATCGGTATGATCTGTACATGATTCGTCAGTGTGAGCAC CACTGACGAGCAGATTTCATTAAACTACGATGTAAACATCAAG

Table S4C - Primers used to construct S. cerevisiae glycolytic expression vectors

ScPGK1 cassette	9421 PGK1 sc prom fw Ytoolkit	AAGCATCGTCTCATCGGTCTCAAACGTATTTTAGATTCCTGA CTTCAACTC
	10764 PGK1 sc term rv Ytoolkit	TTATGCCGTCTCAGGTCTCACAGCCGAAATAATATCCTTCTC GAAAG
ScGPM1 cassette	9757 pGPM1 sc fw Ytookit	AAGCATCGTCTCATCGGTCTCAAACGGTGATACTTTGACAGG AGC
	10760 GPM1 sc term rv Ytoolkit	TTATGCCGTCTCAGGTCTCACAGCCATTAAACTACGATGTAA ACATC
ScTDH3 cassette	10753 TDH3 sc prom fw Ytoolkit	AAGCATCGTCTCATCGGTCTCAAACGCGAATATATACTAGCG TTGAATGTTAG
	10762 TDH3 sc term rv Ytoolkit	TTATGCCGTCTCAGGTCTCACAGCGTAACTTCAGAATCGTTA TCCTGG
ScPYK1 cassette	10608 PYK1 sc prom fw Ytoolkit	AAGCATCGTCTCATCGGTCTCAAACGCCCTGGTCAAACTTCA GAAC
	10887 PYK1 sc term rev Ytoolkit	TTATGCCGTCTCAGGTCTCACAGCGTATCCTTTCGCCATCCTG
ScTPI1 cassette	9423 TPI1 sc prom fw Ytoolkit	AAGCATCGTCTCATCGGTCTCAAACGACCCAGAGATGTTGTT GTCC
	10766 TPI1 sc term rv Ytoolkit	TTATGCCGTCTCAGGTCTCACAGCCGGTACACTTCTGAGTAA C
ScPGI1 cassette	9630 PGI1 sc prom fw Ytoolkit	AAGCATCGTCTCATCGGTCTCAAACGTATTCTTAGTGGATAA CATGCG
	10772 PGI1 sc term rv Ytoolkit	TTATGCCGTCTCAGGTCTCACAGCGAAATAGGACCTGATATC CTCC
ScFBA1 cassette	9419 FBA1 sc prom fw Ytoolkit	AAGCATCGTCTCATCGGTCTCAAACGCAATACCAGCCTTCCA ACTTC
	10758 FBA1 sc term rv Ytoolkit	TTATGCCGTCTCAGGTCTCACAGCCGCGAACTCCAAAATGAG C
ScPDC1 cassette	9755 PDC1 sc prom fw Ytoolkit	AAGCATCGTCTCATCGGTCTCAAACGCATGCGACTGGGTGA GCATATG
	10774 PDC1 rv term rv Ytoolkit	TTATGCCGTCTCAGGTCTCACAGCCAGTGTTCCTTAATCAAG GATACC
ScADH1 cassette	9733 ADH1 sc prom fw Ytoolkit	AAGCATCGTCTCATCGGTCTCAAACGAAGTCCAATGCTAGTA GAGAAG
	10770 ADH1 sc term rv Ytoolkit	TTATGCCGTCTCAGGTCTCACAGCCAACAGGTGTTGTCCTCT G
ScHXK2 cassette	9417 HXK2 sc prom fw Ytoolkit	AAGCATCGTCTCATCGGTCTCAAACGCTGGTAAAGTACAGCT ACATTC
	12927 YTK HXK2 REV	ATGCCGTCTCAGGTCTCACAGCACGCTACAAAAGAAAGTAC GCAAG
ScENO2 cassette	9739 ENO2 sc prom fw Ytoolkit	AAGCATCGTCTCATCGGTCTCAAACGGGATGATGAAAACAC TAAACGAAG
	12930 YTK ENO2 REV	ATGCCGTCTCAGGTCTCACAGCAGGTATCATCTCCATCTCCC
ScPFK1 cassette	9634 PFK1 sc prom fw Ytoolkit	AAGCATCGTCTCATCGGTCTCAAACGCGGCTAGTAAAAAAG AAAATTAATATCTCATTAAC

	12928 YTK PFK1 REV	ATGCCGTCTCAGGTCTCACAGCCACATTCAGAGCAATTTGTAGTAC
ScPFK2 cassette	10614 PFK2 sc prom fw Ytoolkit	AAGCATCGTCTCATCGGTCTCAAACGCCATTCTCTGCTGCTTTGTTG
	12929 YTK PFK2 REV	ATGCCGTCTCAGGTCTCACAGCATAAGAGAACAAAGTATTTAACGC

Table S4D - Primers used to amplify expression cassettes

Fragment	Primer name	Sequence
FBA1 cassette	12952 tFBA1 + can1	GTTTTTAATCTGTCGTCAATCGAAAGTTTATTTCAGAGTTCTT CAGACTTCTTAACTCCTGTGCATGACAAAAGATGAGCTAGG
	12446 pFBA+ BA	TAAGTCTCTTGACATCTCGGAACATATCCACTCAGCGGTGT ATCATTCTGTGGTCGGCGCCATGCCTCCAACGGCTACTATC
PGI1 cassette	12449 pPGI1 + BA	GCGCCGACCACAGAATGATACACCGCTGAGTGGATATGTT CCGAGATGTCAAGAGACTTATCTTAGTGGATAACATGCGGC
	12450 tPGI1 + BI	TCTGTCAGTTGGTTAAGCGCCGCTACGATTACTACACATGCC ACAGACTGATCTACAATGTATCCTCCTTTTAAACAGTTGATG
PGK1 cassette	12474 tPGK1 + BI	CATTGTAGATCAGTCTGTGGCATGTGTAGTAATCGTAGCGGC GCTTAACCAACTGACAGATGGCAGCCGAAATAATATCCTTC
	15008 pPGK1 + BJ	GAGGCTTCACAGTGCTTTATTAGTATGATTGCCTAGCTGGTAT ATGTGTTCCTGGAGCGCTTCCTGACTTCAACTCAAGACGC
TDH1 cassette	12457 tTDH3 + BJ	TAGAGAGGATCACACCCAGCTATGTTGCCGCATCTCCGAT CATAATATACCATGTGCGCCCAGAATCGTTATCCTGGCGG
	12458 pTDH3 + BE	TCAATCATTCGTTCTCGCAGATCTACAATCGTCCTGAGCTCT GTGAGTGATGTACGCTCCTACTAGCGTTGAATGTTAGCGTC
ENO1 cassette	12459 pENO2 + BE	GGAGCGTACATCACTCACAGAGCTCAGGACGATTGTAGATC TGCGAGAACGAATGATTGATGATGAAAACACTAAACGAAGG
	12460 tENO2 + BF	GCGCGACGTGTCTCGTATATTAGTGAAGTTGGATCTGTCCA TGAATCCTCGGCTCTGGTGTATTTTTCAAACTGCAAATTCAAG
ScADH1 cassette	12461 tADH1 + BF	CACCAGAGCCGAGGATTCATGGACAGATCCAACTTCACTA ATATACGAGACACGTCGCGCATGCCGGTAGAGGTGTGGTC
	14487 pADH1 + BC	CTAGGCTCTGCTGCATGTCAGTGATTTCTATTAGGCAGCGCT TACCCATGATTAGCGCAGAGTCCAATGCTAGTAGAGAAGGG
ScPDC1 cassette	12465 tPDC1 + BC	CTGCGCTAATCATGGGTAAGCGCTGCCTAATAGAAATCACT GACATGCAGCAGAGCCTAGTGTTCCTTAATCAAGGATACCTC
	12466 pPDC1 + BD	AGTCACGCTGAGTCCATGCTGACCATGATTCACACTCAGT GCCGATAATTCCATAGTCTGCGACTGGGTGAGCATATGTTC
GPM cassette	12467 pGPM1 + BD	CAGACTATGGAATTATCGGCACTGAGTGTGAATCATGGTCA GCATGGACTCAGCGTGACTGATACTTTGACAGGAGCTATATC
	12468 tGPM1 + BK	GAGCATACTGTCCTATCATGTCGACTCTTGTCACATCTGAC GCCTCTCTGCGATAGGATTTGCTATAACATGTCATGTCACC
TPI1 cassette	12469 tTPI1 + BK	AATCCTATCGCAGAGAGGCGTCAGATGTGACAAGAGTCGAC ATGATAGGACAGTATGCTCTGAGTAACCCATATAGAGATCG

	12470 pTPI + can1	GTGTATGACTTATGAGGGTGAGAATGCGAAATGGCGTGGA AATGTGATCAAAGGTAATAACCAGAGATGTTGTTGTCCTAG
GroEL cassette	10807 GroEL in X2 fw	GCTGAAGATTTATCATACTATTCCTCCGCTCGTTTCTTTTTT CAGTGAGGTGTGTCGTGAGATATCATCACTCTTACCAGGCTAGG
	10808 GroEL + AA rev	ATAGCATAGGTGCAAGGCTCTCGCCGCTTGTCGAGCTATTGG CATGGATGTGCTCCCTAACAGGATATCCTGGACCTTAATCG
GroES cassette	10809 GroES + AA fw	TTAGGGAGCACATCCATGCCAATAGCTCGACAAGCGGCGAGAG CCTTGCACCTATGCTATATCTACGTATGGTCATTTCTTCTTCAG
	10810 GroES + X2 flnk rev	ATTCTCGCCAAGGCATTACCATCCCATGTAAGAACGGAATAAA ACAGCATTCGAAGGTTATTCGCGACACAATAAAGTCTTC
PYK1 cassette	16005 pPYK1 + SPR3	AGAAATAAATAAATAAATAATAAAAACCTAAAATTCCTTT TGCGTCATTGAATTTTTATTCGAAAGTTTTTCCGGCAAGC
	15977 tPYK1 + BG	GAGGCTTCACAGTGCTTTATTAGTATGATTGCCTAGCTGG TATATGTGTTCCTGGAGCGCGCGTATCCTTTCGCCATCC
PFK cassette	16053 pTEF1 + BG	CGCTCCAGGAACACATATACCAGCTAGGCAATCATACTAA TAAAGCACTGTGAAGCCTCCGCGAATCCTTACATCACAC
	16054 tTEF1 + BH	AGGATCGCTCGCGTACTCATGCATTCTCCCACATATTGAG GCCCTGATTCCATGCAATGTGTCATCCGAGCGTGTATTGC
GLK cassette in *Yl*-Glycolysis strain	15732 pACT1 + BH	ACATTGCATGGAATCAGGGCCTCAATATGTGGGAGAATGCATG AGTACGCGAGCGATCCTGCCATGGCTAGACAAATCAAGGAAAG
	16006 tENO1 + SPR3	CAGCAAGTGCGTAGAGATCAGCATTATCTGACTGTGGATGA TCCTACATCGTCATCAGAGATACATGGGTGACCAAAAGAGC
ScPFK1 cassette in *Yl*-Glycolysis *ScPFK* strain	15731 pPFK1+BG fw	GCGCTCCAGGAACACATATACCAGCTAGGCAATCATACTAATAAAG CACTGTGAAGCCTCGCGGCTAGTAAAAAAGAAAATTAATATCTCATTAAC
	12452 tPFK1+BH rv	AGGATCGCTCGCGTACTCATGCATTCTCCCACATATTGAGGCCCTGATT CCATGCAATGTACTTGAATAATGCAAATTCCATAGC
ScPFK2 cassette in *Yl*-Glycolysis *ScPFK* strain	12472 pPFK2 + BL fw	CTCTGATGACGATGTAGGATCATCCACAGTCAGATAATGCTGATCTCTA CGCACTTGCTGATTCTCTGCTGCTTTGTTG
	16007 tPFK2 + SPR3	TTTTTATTATGTAGAGCAAAGCTTGCGCGAAATTATTGGCTTTTTTTTTT TTTAATTAATTTAAATCGTCTATATCACATATTCCAG
YlGLK cassette in *Yl*-Glycolysis *ScPFK* strain	15732 pACT1 + BH	ACATTGCATGGAATCAGGGCCTCAATATGTGGGAGAATGCATG AGTACGCGAGCGATCCTGCCATGGCTAGACAAATCAAGGAAAG
	15733	

URA3	9337	TCGGTCTCATACACGGTTTCC
	9338	TGGTCTGGTCTCAACTCGG
GLK cassette for X2 integration	13596 X2 flank fw	GCTGAAGATTTATCATACTATTCCTCCGCTCGTTTCTTTTTTCA GTGAGGTGTGTCGTGATGAACTGGCCGATAATTGCAGA
	13597 X2 flank rv	ATTCTCGCCAAGGCATTACCATCCCATGTAAGAACGGAATAAAAC AGCATTCGAAGGTTATGATGACCCCGTCGTCTCATT

Primers for amplification of expression cassettes *Sc-3K* strains		
PGK1 cassette	tPGK1 + BI 12474	CATTGTAGATCAGTCTGTGGCATGTGTAGTAATCGTAGCGG CGCTTAACCAACTGACAGATGGCAGCCGAAATAATATCCTTC
	pPGK1 + BG 12475	GAGGCTTCACAGTGCTTTATTAGTATGATTGCCTAGCTGGTA TATGTGTTCCTGGAGCGCTTCCTGACTTCAACTCAAGACGC
PFK1 cassette	pPFK1 + BG 12451	GCGCTCCAGGAACACATATACCAGCTAGGCAATCATACTAAT AAAGCACTGTGAAGCCTCGGGATAGCGGCTAGTAAAAAG
	tPFK1 + BH 12452	AGGATCGCTCGCGTACTCATGCATTCTCCCACATATTGAGGC CCTGATTCCATGCAATGTACTTGAATAATGCAAATTCCATAGC
PYK1 cassette	pPYK1 + BH 12455	ACATTGCATGGAATCAGGGCCTCAATATGTGGGAGAATGCAT GAGTACGCGAGCGATCCTCCTGGTCAAACTTCAGAACTAAG
	tPYK1 + BJ 12456	GGCGCACATGGTATATTATGATCGGAGATGCGGCAACATAG CTGGGTGTGATCCTCTCTACGTATCCTTTCGCCATCCTG
ADH1 cassette	tADH1 + BF 12461	CACCAGAGCCGAGGATTCATGGACAGATCCAACTTCACTAA TATACGAGACACGTCGCGCATGCCGGTAGAGGTGTGGTC
	pADH1 + BB 12462	GCAACGCATTCCATACATGATGCGTTGCTTGGTGTCCACAGC CGTACTTGAGAAGCTCTGAGTCCAATGCTAGTAGAGAAGGG
HXK2 cassette	pHXK2 + BB 12463	CAGAGCTTCTCAAGTACGGCTGTGGACACCAAGCAACGCAT CATGTATGGAATGCGTTGCGCTGGTAAAGTACAGCTACATTC
	tHXK2 + BC 12464	CTAGGCTCTGCTGCATGTCAGTGATTTCTATTAGGCAGCGCT TACCCATGATTAGCGCAGACTTGAACAATAAATACGAAATCC
TPI1 cassette	tTPI1 + BK 12469	AATCCTATCGCAGAGAGGCGTCAGATGTGACAAGAGTCGAC ATGATAGGACAGTATGCTCTGAGTAACCCATATAGAGATCG
	pTPI + can1 12470	GTGTATGACTTATGAGGGTGAGAATGCGAAATGGCGTGGA AATGTGATCAAAGGTAATAACCAGAGATGTTGTTGTCCTAG
PFK2 cassette	pPFK2 + BL 12472	CTCTGATGACGATGTAGGATCATCCACAGTCAGATAATGC TGATCTCTACGCACTTGCTGATTCTCTGCTGCTTTGTTG
	tPFK2 + CAN1 12473	GTGTATGACTTATGAGGGTGAGAATGCGAAATGGCGTGGAA ATGTGATCAAAGGTAATAAAATCGTCTATATCACATATTCCAG

Table S4E - Diagnostic primers

Fragment	Primer name	Sequence
Synthetic glycolytic loci diagnostic primers		
CAN1 – FBA1	3491 CAN1 fw	ATCACTTACTGGCAAGTGCG
	5389 FBA1 rv	GTTCTTCCTTGCGTTATTCTTCTG
FBA1 – PGI1	2373 FBA1 fw	GTTACGTGCTCAGTTGTTAGATATG
	5925 PGI1 rv	TTTTAGGCTGGTATCTTGATTCTAAATCG
PGI1 – PGK1	4671 PGI1 fw	ACAAATCGCTCTTAAATATATACCTAAAGAAC
	6488 PGK1 rv	ATTGAATTGAATTGAAATCGATAGATCAATTTTTTTC
PGK1 – TDH1	5647 PGK1 fw	CGTCGCTAGGACCTTGTTG
	5756 TDH1 rv	CGCCACATGTAATATCTGTAGTAGATACC
TDH1 – ENO1	5030 TDH1 fw	GGCAGTATTGATAATGATAAACTCGAAC
	3364 ENO1 rv	TATGCTGACTTGGTATCACACTTC
ENO1 – ADH1	12382 ENO1 fw	ATCCTAACTCGAGAGTGCTTTTAAC
	7494 ADH1 rv	GTAGCCCTAGACTTGATAGCC
ADH1 – PDC1	7496 ADH1 fw	CAGCTCTGGAACAACGACATCTG
	757 PDC1 rv	GCTTTCGTCACCCCAATGG
PDC1 – GPM1	2851 PDC1 fw	TTGCGTGAGGTTATGAGTAG
	5036 GPM1 rv	GGTTACTTAGACATCACTATGGC
GPM1 – TPI1	13743 GPM1 fw	TTTTCAGCCTGTCGTGGTAGC
	3515 TPI1 rv	CTGACAGGTGGTTTGTTACG
TPI1 – CAN1	2909 TPI1 fw	CCCGCTCACACTAACGTAGG
	12241 CAN1 rv	GGTTCTAGGTTCGGGTGACG
GroEL/ES integration diagnostic primers		
X2- GroEL	13662	TCCTCGGGCAGAGAAACTCG
	11033	TCCATTGGGTTCATACCAGC
GroEL-GroES	2647	TCCGGGCAACGGTATTTC
	4663	CTCTTCGTATGTCCATCTAAACC
GroES – X2	2676	CGACGGTTACGGTGTTAAG
	13663	GTGAGCCTCTTACCTGTTTG
URA3 integration, key-point integration in *spr3*, SinLoG removal diagnostic primers, *URA3* deletion check		
URA3 in tdh1	1989	CCACGTGCAGAACAACATAG
	1990	ATAGTCACATATTGTGGGTATGTG
SPR3-PYK1	3832	TTGCCATTTGCTGCATCC
	8743	GGAAAGGAAATCACTTGGAAGA
PYK1-TEF1p	13735	TCCAATTGTCGTCATAACGATGAGG
	8410	CGACGAAGAAAAAGAAACGAGG
TEF1t-ACT1p	10216	GGAGATTGATAAGACTTTTCTAGTTG
	13078	AGAGAGAGAGGCGAGTTTGG
ENO1t-SPR3	11904	GATTAAGCCTTCTAGTCCAAAAAACACG
	92	ATGATGTCGCGCATTTGATGCCTTAAATAC
URA3 deletion check complementation strains	10326	AATACACGCTCGGATGACTG
	2644	AATCATTACGACCGAGATTC
Confirmation removal SinLoG cassette *sga1*	11898_SeqFW_SGA	CGCGGAAACGGGTATTAGGG
	11899_SeqRV_SGA	CTAGATCCGGTAAGCGACAG
Confirmation removal SinLoG cassette *sga1*	4226	ACTCGTACAAGGTGCTTTTAACTTG
	4457	TTGGGCTGGACGTTCCGACATAG
URA3	2891	CATGGAGGGCACAGTTAAGC

Sanger seq	1522	CGAGATTCCCGGGTAATAACTG
TPS1 deletion check	4263	TGGTGGAGACGCTTGATTTG
	4264	TCGTTATGCGGTGTGAACAG

Verification plasmids and plasmid integration complementation strains		
pHXK2-tHXK2	3481	GCCTAGCGTCTGGGATTTATTC
	10325	AGTCATCCGAGCGTGTATTG
pPYK1-tPYK1	1152	TGGCGTGTGATGTCTGTATCTG
	4667	CCTTGAGGGAAGATTATCTTGCG
pTEF1-tTEF1	6717	CTCATTAGAAAGAAAGCATAGCAATC
	14416	GAAATGATATTTTAGAATAACCAGAC
pACT1-tENO1	14484	CACGCTTACTGCTTTTTTCTTCCC
	2306	ACATGGGTGACCAAAAGAGC
YlPFK-YlPFK	15259	CCCATATTCTTCCGCTATGC
	15260	ATGGCATCAATGGCTTCAAC
pPYK1-YlPYK1	11915	GAGTGAGTGCTTTGTTCAATGG
	16056	GTCTCGACCTTCAAAGTTTCGCC
pUDI-*URA3*	9441	AGAGCACTTGAATCCACTGC
	4728	CCAGCCCATATCCAACTTCC
URA3-pUDI	7653	ATTCCAACTAATGAGATGGAATCG
	9442	GTAATGTTATCCATGTGGGC
URA3 - pPYK1	7653	ATTCCAACTAATGAGATGGAATCG
	7428	TGTGATGATGTTTTATTTGTTTTGATTGG
URA3-pPFK1	7653	ATTCCAACTAATGAGATGGAATCG
	8410	CGACGAAGAAAAAGAAACGAGG
Sanger sequencing verification *YlGLK1*		
tENO1	2306	ACATGGGTGACCAAAAGAGC
YlGLK1 seq primer 1	18645	AACCAAGAAGAAAAAGAAAAGG
YlGLK1 seq primer 2	18646	TGGCTCAACAAGTTAAGGAC
YlGLK1 seq primer 3	18647	CCAAAGCAACTATCTTAACG
YlGLK1 seq primer 4	18648	CACCGAATGGGGTTCTTACG
YlGLK1 seq primer 5	18649	GAGGTATCCATACCAAAACC
YlGLK1 seq primer 6	18650	GTTGCAATCTACTAAGTTGG
YlGLK1 seq primer 7	18651	TAACCAATGGCTTCAAAGCA
Verfication integration *YlGLK1* in 18648X2		
X2-X2	13662	TCCTCGGGCAGAGAAACTCG
	13078	GTGAGCCTCTTACCTGTTTG
Deletion native *HXK2/PYK1/PFK1/PFK2* diagnostic primers		
HXK2 deletion check	3481	GCCTAGCGTCTGGGATTTATTC
	3070	AGTGCTTCCGTTCGTTCCAG
PFK1 deletion check	4925	AATTTTTACCCTGATCTAACTAACTTTGG
	4924	GTAGACCGATGACAATACGACTAC
PFK2 deletion check	4777	CGTGAGCCTTAACCAATGAG
	4776	CTCCGTTCTTCGTGATAAGTTC
PYK1 deletion check	1152	TGGCGTGTGATGTCTGTATCTG
	4667	CCTTGAGGGAAGATTATCTTGCG
Check integration mosaic SinLoG *Sc*-3K strain		

PGK1 – PFK	2684	AAGGATTCGCGCCCAAATCG
	2368	AATCATGTTGATGACGACAATGG
PFK – PYK	6501	ATGATTGCAATGAAAAGTTTAAGTTAAGCAAAAG
	8743	GGAAAGGAAATCACTTGGAAGA
PYK - TDH	2914	GTCGTCATAACGATGAGGTGTTGC
	6493	GTGAATTTACTTTAAATCTTGCATTTAAATAAATT
ADH - HXK	7496	CAGCTCTGGAACAACGACATCTG
	5001	CCAATGTGCGAGGAGGTTCAG
HXK - PDC	2429	TCACGGGATTTATTCGTGACG
	2852	GCCAACTTTCGGTGCTAAGGAC
TPI1 – PFK2	2374	GCAGAAGTGTCTGAATGTATTAAGG
	2433	GACGCCATTTGGAACGAAAAAAAG
PFK2 – CAN1	2370	AAACTGAAGTTTCCATGAGAATGC
	3492	ATCAGTTGTGCCTGGAAAAG

Check integration mosaic SinLoG *Yl*-3K strain		
can1-tFBA1	12240	TTCTGTGTGGTTTCCGGGTG
	6483	GTTAATTCAAATTAATTGATATAGTTTTTTAATGAGTATTGAATC
pFBA1-pPGI	5026	CGTATTACGATAATCCTGCTGTC
	11923	CCACCCAGATCGTGATTTTT
pPGI1-tPGK1	2430	GCGTCCAAGTAACTACATTATGTG
	7084	ATTGAATTGAATTGAAATCGATAG
pPGK1-pTEF1	2684	AAGGATTCGCGCCCAAATCG
	3223	GACACCCTAGAGGAAGAAAG
tTEF1-pPYK1	10216	GGAGATTGATAAGACTTTTCTAGTTG
	8743	GGAAAGGAAATCACTTGGAAGA
tPYK1-tTDH3	2914	GTCGTCATAACGATGAGGTGTTGC
	6493	GTGAATTTACTTTAAATCTTGCATTTAAATAAATT
-pTDH3-pENO2	4369	TGGGCATGTACGGGTTACAG
	6340	TATTATTGTATGTTATAGTATTAGTTGCTTGGTGTTATG
tENO2-ADH1	3365	CAAAGACTCGTGCTGTCTATTGC
	5295	GGAATACAAAGATATTCCAGTTCCAAAGCC
ADH1-pACT1	7496	CAGCTCTGGAACAACGACATCTG
	13078	AGAGAGAGAGGCGAGTTTGG
tENO1-PDC1	11904	TTGTGGTGACGCGTGTATCC
	2852	GCCAACTTTCGGTGCTAAGGAC
PDC1-pGPM1	6351	TTTGATTGATTTGACTGTGTTATTTTGCG
	3367	ACGGAAAGTGGAATCCCATTTAG
tGPM1-tTPI1	5757	CGTCAGGGACAGTATGTTGGAATG
	3514	CTGACAGGTGGTTTGTTACG
	2531	TCCCGTTAGGAACATTGG

pTPI1-can1	3492	ATCAGTTGTGCCTGGAAAAG

Table S4F - gRNA oligo's and repair fragments

Target	Primer name	Sequence
URA3 deletion SwYG strains	8553 URA3_repair oligo fw	TGCCCAGTATTCTTAACCCAACTGCACAGAACAAAAACCTG CAGGAAACGAAGATAAATCAAAACTGTATTATAAGTAAATG CATGTATACTAAACTCACAAATTAGAGCTTCAATTTAA
	8554 URA3_repair oligo rv	TTAAATTGAAGCTCTAATTTGTGAGTTTAGTATACATGCATT TACTTATAATACAGTTTTGATTTATCTTCGTTTCCTGCAGGT TTTTGTTCTGTGCAGTTGGGTTAAGAATACTGGGCA
URA3 deletion complementation strains	13807 URA3_repair_fw	CGGTTTCCTTGAAATTTTTTTGATTCGGTAATCTCCGAACAGA AGGAAGAACGAAGGAAGGGAATCTCGGTCGTAATGATTTCT ATAATGACGAAAAAAAAAAAAATTGGAAAGAAAAAGC
	13808 URA3_repair_rv	GCTTTTTCTTTCCAATTTTTTTTTTTTTCGTCATTATAGAAATCAT TACGACCGAGATTCCCTTCCTTCGTTCTTCCTTCTGTTCGGAG ATTACCGAATCAAAAAAATTTCAAGGAAACCG
Deletion glycolytic genes *sga1*	6075 COUNTER SELECT oligo fw	TTTTTCTCATCTCTTGGCTCTGGATCCGTTATCTGTTCTGTTA CACAAGAAATCGTACATACTAGAGCAAGATTTCAAATAAGT AACAGCAGCCATACGTTGAAACTACGGCAAAGGATT
	6076 COUNTER SELECT oligo rv	AATCCTTTGCCGTAGTTTCAACGTATGGCTGCTGTTACTTATT TGAAATCTTGCTCTAGTATGTACGATTTCTTGTGTAACAGAA CAGATAACGGATCCAGAGCCAAGAGATGAGAAAAA
gRNA *PYK1*	10974	TGCGCATGTTTCGGCGTTCGAAACTTCTCCGCAGTGAAAGAT AAATGATCTATCAACTTCGGTATTGAAAGTTTTAGAGCTAGA AATAGCAAGTTAAAATAAGGCTAGTCCGTTATCAAC
	10975	GTTGATAACGGACTAGCCTTATTTTAACTTGCTATTTCTAGCT CTAAAACTTTCAATACCGAAGTTGATAGATCATTTATCTTTCA CTGCGGAGAAGTTTCGAACGCCGAAACATGCGCA
Repair *HXK2* deletion	5888	TTTCTAATGCCTTTTCCATCATGTTACTACGAGTTTTCTGAACC TCCTCGCACATTGGTAGCTTAATTTTAAATTTTTTTGGTAGTAA

		AAGATGCTTATATAAGGATTTCGTATTTATTG
	5889	CAATAAATACGAAATCCTTATATAAGCATCTTTTACTACCAAA AAAATTTAAAATTAAGCTACCAATGTGCGAGGAGGTTCAGA AAACTCGTAGTAACATGATGGAAAAGGCATTAGAAA
Repair *PFK1* deletion	10211	AATTAATATCTCATTAACAAAGTTATTGTACATAATCCGGTAC AATATTCTTCAATGTACGTTTTAGGGTGTGCTTAATCTGCGTT GACAATGGTTCACGAAGACGACATCGGCAACTTT
	10212	AAAGTTGCCGATGTCGTCTTCGTGAACCATTGTCAACGCAGAT TAAGCACACCCTAAAACGTACATTGAAGAATATTGTACCGGAT TATGTACAATAACTTTGTTAATGAGATATTAATT
Repair *PFK2* deletion	10209	CCAGTCCCGCATACCCCCTTTGCAACGTTAACGTTACCGCTAG CGTTTACCATCTCCACGACTTATGTATACTGGAATATGTGATA TAGACGATTTAAAAGATAATTCCAATAAACGTCC
	10210	GGACGTTTATTGGAATTATCTTTTAAATCGTCTATATCACATAT TCCAGTATACATAAGTCGTGGAGATGGTAAACGCTAGCGGTA ACGTTAACGTTGCAAAGGGGGTATGCGGGACTGG
Repair *PYK1* deletion	10982	ATTATTCTCTCTTGTTTCTATTTACAAGACACCAATCAAAACAA ATAAAACATCATCACAAAAAAGAATCATGATTGAATGAAGAT ATTATTTTTTTGAATTATATTTTTTAAATTTTAT
	10983	ATAAAATTTAAAAAATATAATTCAAAAAAATAATATCTTCATT CAATCATGATTCTTTTTTGTGATGATGTTTTATTTGTTTTGATT GGTGTCTTGTAAATAGAAACAAGAGAGAATAAT
gRNA *TPS1*	16082	TGCGCATGTTTCGGCGTTCGAAACTTCTCCGCAGTGAAAGAT AAATGATCTACAATAATAGCACCATTCAGTTTTAGAGCTAGA AATAGCAAGTTAAAATAAGGCTAGTCCGTTATCAAC
	16083	GTTGATAACGGACTAGCCTTATTTTAACTTGCTATTTCTAGCTC TAAAACTGAATGGTGCTATTATTGTAGATCATTTATCTTTCACT GCGGAGAAGTTTCGAACGCCGAAACATGCGCA
Repair *TPS1* deletion	16084	AGCAACAAAGCAGGCTAACAAACTAGGTACTCACATACAGA CTTATTAAGACATAGAACTTGAACCCGATGCAAATGAGACG ATCGTCTATTCCTGGTCCGGTTTTCTCTGCCCTCTCTT
	16085	AAGAGAGGGCAGAGAAAACCGGACCAGGAATAGACGATCGT CTCATTTGCATCGGGTTCAAGTTCTATGTCTTAATAAGTCTGT ATGTGAGTACCTAGTTTGTTAGCCTGCTTTGTTGCT
gRNA's X2 and *URA3* for pUDR591	8313 *URA3* gRNA	TGCGCATGTTTCGGCGTTCGAAACTTCTCCGCAGTGAAAGATA AATGATCAACAAACTTGTGTGCTTCATGTTTTAGAGCTAGAAA TAGCAAGTTAAAATAAG
	10866 X2 gRNA	TGCGCATGTTTCGGCGTTCGAAACTTCTCCGCAGTGAAAGATA AATGATCGGCGACTAGGAAGAGAGTAGGTTTTAGAGCTAGAA ATAGCAAGTTAAAATAAG
gRNA SPR3 in pUDR596	12034	TGCGCATGTTTCGGCGTTCGAAACTTCTCCGCAGTGAAAGATAA ATGATCATGCTTTTATAACGAATAATGTTTTAGAGCTAGAAATA GCAAGTTAAAATAAGGCTAGTCCGTTATCAAC

References

1. Zak, K.M., et al., *Crystal Structure of Kluyveromyces lactis Glucokinase (KlGlk1).* Int J Mol Sci, 2019. **20**(19): p. 4821.
2. Stoddard, P.R., et al., *Polymerization in the actin ATPase clan regulates hexokinase activity in yeast.* Science, 2020. **367**(6481): p. 1039-1042.
3. Flores, C.-L., C. Gancedo, and T. Petit, *Disruption of Yarrowia lipolytica TPS1 gene encoding trehalose-6-P synthase does not affect growth in glucose but impairs growth at high temperature.* PloS one, 2011. **6**(9): p. e23695.
4. Kuijpers, N.G., et al., *Pathway swapping: Toward modular engineering of essential cellular processes.* Proceedings of the National Academy of Sciences, USA, 2016. **113**(52): p. 15060-15065.
5. Boonekamp, F.J., et al., *Full humanization of the glycolytic pathway in Saccharomyces cerevisiae.* Cell reports, 2022. **39**(13).
6. Entian, K.-D. and P. Kötter, *25 Yeast Genetic Strain and Plasmid Collections*, in *Yeast Gene Analysis - Second Edition*, I. Stansfield and M.J.R. Stark, Editors. 2007, Academic Press. p. 629-666.
7. Mans, R., et al., *CRISPR/Cas9: a molecular Swiss army knife for simultaneous introduction of multiple genetic modifications in Saccharomyces cerevisiae.* FEMS Yeast Research, 2015. **15**(2).
8. Solis-Escalante, D., et al., *A minimal set of glycolytic genes reveals strong redundancies in Saccharomyces cerevisiae central metabolism.* Eukaryotic Cell, 2015. **14**(8): p. 804-816.
9. Lee, M.E., et al., *A highly characterized yeast toolkit for modular, multipart assembly.* ACS Synthetic Biology, 2015. **4**(9): p. 975-986.
10. Gorter de Vries, A.R., et al., *CRISPR-Cas9 mediated gene deletions in lager yeast Saccharomyces pastorianus.* Microb Cell Fact, 2017. **16**(1): p. 222.
11. Postma, E.D., et al., *A supernumerary designer chromosome for modular in vivo pathway assembly in Saccharomyces cerevisiae.* Nucleic Acids Res, 2021. **49**(3): p. 1769-1783.
12. Guadalupe-Medina, V., et al., *Carbon dioxide fixation by Calvin-Cycle enzymes improves ethanol yield in yeast.* Biotechnology for biofuels, 2013. **6**(1): p. 125.
13. Orij, R., et al., *In vivo measurement of cytosolic and mitochondrial pH using a pH-sensitive GFP derivative in Saccharomyces cerevisiae reveals a relation between intracellular pH and growth.* Microbiology, 2009. **155**(1): p. 268-278.
